# miRNA expression profile of bone marrow resident cells from children with neuroblastoma is not significantly different from that of healthy children

**DOI:** 10.18632/oncotarget.24874

**Published:** 2018-04-10

**Authors:** Sara Stigliani, Fabio Morandi, Luca Persico, Corrado Lagazio, Giovanni Erminio, Paola Scaruffi, Maria Valeria Corrias

**Affiliations:** ^1^ Physiopathology of Human Reproduction, Ospedale Policlinico San Martino, Genoa, Italy; ^2^ Experimental Therapy in Oncology, IRCCS Istituto Giannina Gaslini, Genoa, Italy; ^3^ Department of Economy, University of Genoa, Genoa, Italy; ^4^ Epidemiology, Biostatistics and Committees, IRCCS Istituto Giannina Gaslini, Genoa, Italy; ^5^ Present address: Stem Cell Laboratory and Cell Therapy Center, IRCCS Istituto Giannina Gaslini, Genoa, Italy

**Keywords:** miRNA, neuroblastoma, bone marrow, mitochondria, gene expression

## Abstract

The miRNA expression profiles of bone marrow resident cells from children with neuroblastoma were compared to that of healthy children. No significant difference was found between localized and metastatic neuroblastoma, or between children with neuroblastoma and healthy children. By considering the fold change we identified six miRNAs over-expressed by more than 150 fold in neuroblastoma. Validation confirmed miR-221 over-expression in BM resident cells from children with neuroblastoma, regardless of localized or metastatic disease.

MiR-221 over-expression was unlikely derived from neuroblastoma primary tumors or from bone marrow-infiltrating metastatic cells, since neuroblastoma cells expressed lower or similar amount of miR-221 than BM cells, respectively.

To get insight on the genes potentially regulated by miR-221 we merged the list of miR-221 potential targets with the genes under-expressed by BM resident cells from children with neuroblastoma, as compared with healthy children. *In silico* analysis demonstrated that none of the miR-221 target genes belonged to heme biosynthetic processes found altered in children with neuroblastoma, whereas two genes associated with mitochondria. However, the encoded proteins were not under-expressed in children with neuroblastoma, making unlikely that altered erythrocyte maturation in children with neuroblastoma was mediated by miR-221.

In conclusion, miRNA expression profiles of BM resident cells from children with localized and metastatic neuroblastoma were similar to that of BM resident cells from healthy children. Moreover, miRNAs expressed by neuroblastoma primary tumors or by BM-infiltrating NB cells do not appear to be involved in mediating the functional defect of erythrocyte maturation recently observed in children with neuroblastoma.

## INTRODUCTION

MiRNAs are 20–22 nucleotide-long non coding RNAs that, following binding to complementary sequences in the 3′ UTR of an mRNA, repress translation or induce mRNA degradation. Therefore, miRNAs are considered major regulators of gene expression in both normal and neoplastic cells [1]. Since they can be found in plasma, either free or inside tumor-derived vesicles that can fuse to cells of different lineages, miRNAs can regulate gene expression also at distance [2–4].

Neuroblastoma (NB) is a pediatric tumor with a heterogeneous clinical behavior at diagnosis, varying from localized disease with good prognosis to metastatic disease with poor outcome [5]. Metastatic disease mainly involves the bone marrow (BM), and presence of BM-infiltrating NB cells is the main negative prognostic factor [6]. The role of miRNAs in regulating NB pathogenesis and invasiveness has been deeply investigated in human NB primary tumors (see [7, 8] for review) and NB experimental models [9, 10]. Prognostic miRNA expression profiles of human NB primary tumors have been defined [11–13], and public datasets of miRNA expression levels in specific subsets of human NB primary tumors are available (GSE86889, GSE21713, GSE16444) [14–16].

Recently, we showed that in children with NB erythrocyte maturation was selectively impaired at the ortho-chromic stage, regardless of the presence of metastatic cells in the BM [17]. Among the genes significantly under-expressed by BM resident cells from children with NB, as compared with healthy children, we identified a set involved in heme and porphyrin biosynthesis in the mitochondria [17]. Given the pivotal role of miRNAs in regulating gene expression, here we investigated whether the decreased expression of these genes was regulated by specific miRNA(s) over-expressed by BM resident cells of children with NB, as compared with healthy children. No information or public datasets of miRNA expression profiles of BM resident cells from children with NB and from healthy children were in fact available to test this hypothesis.

## RESULTS

### miRNA expression profiles of BM resident cells from children with NB and from healthy children

First, twelve BM resident cell samples from healthy children, twelve from children with localized NB and twelve from children with metastatic NB were identified in our RNA bio bank (Table 1). This cohort reflected the differences between localized and metastatic NB [5, 6]; in fact, children with localized NB were younger than those with metastatic disease, and they did not present *MYCN* amplification in the primary tumors, and were all alive at follow-up.

**Table 1:** Main features of children with NB and healthy children

	Localized NB	%	Metastatic NB	%	Healthy children	%
***N***	12		12		12	
***Stage***	L1/L2	100	M	100	NA	
***Sex***						
F	6	50	4	33.3	6	50
M	6	50	8	66.7	6	50
***Age***						
Median (range)	2 (0.3–6.9)		5.6 (1.0–12.6)		11.2 (4.0–15.0)	
***MYCN***						
Single copy	12	100	7	58.3		
Amplified	0	-	5	41.7		
***State at follow-up***						
Alive	12	100	0	-		
Dead	0	-	12	100		

Then, the expression of 671 unique miRNAs was evaluated by high-throughput RT-qPCR [18] in four samples randomly selected from each group. Despite the clinical, demographic and genetic disparities between children with localized and metastatic NB, the miRNA expression levels in BM resident cells did not show significant differences (MiQe for qPCR in Supplementary Table 1 and raw data in Supplementary Table 2). This finding was in accordance with the absence of significant differences in gene expression profiles of BM resident cells from children with localized and metastatic NB [19].

**Figure 1:**
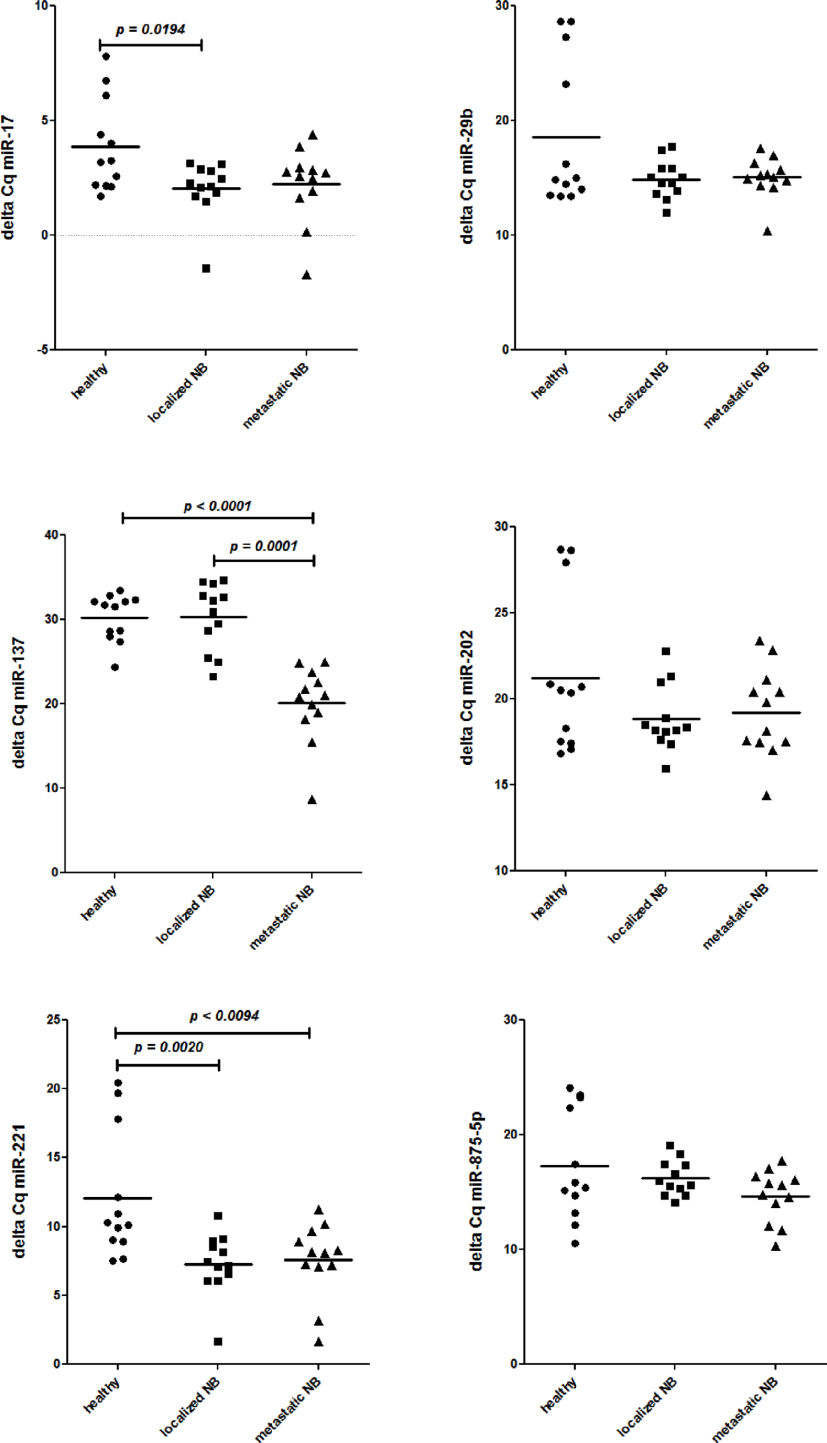
Expression levels (Delta Cq) of miR-17, miR-29b, miR-137, miR-202, miR-221, and miR-875-5p in BM resident cells from healthy children (closed circles) and from children with localized (closed squares) and metastatic (close triangles) NB *N* = 12 for each group.

Thus, the miRNA expression profiles of all children with NB were compared with those of healthy children. Also in this case no significant (Bonferroni’s adjusted *p* value) difference was found. Therefore, to identify miRNAs potentially over-expressed in BM resident cells from children with NB, as compared with healthy children, we ranked miRNAs according to the increased fold change (Table 2). To select miRNAs whose over-expression could be biologically relevant in regulating gene expression we considered only the six-top-ranked miRNAs with a fold increase over 150 fold (0.015 in Table 2). The expression of the six top-ranked miRNAs was then evaluated by specific RT-qPCR in all the samples. As shown in Figure 1, miR-29b, miR-202 and miR-875-5p were found expressed at similar levels in children with localized or metastatic NB and healthy children. MiR-17 was over-expressed in children with localized NB and miR-137 was significantly over-expressed in children with metastatic NB (Figure 1). Thus, only miR-221 was significantly over-expressed by BM resident cells from children with NB regardless of localized or metastatic disease (Figure 1).

**Table 2:** miRNAs potentially over-expressed by resident BM cells from children with NB, as compared with BM resident cells from healthy children, ranked by fold change

miRNA	Fold Change	∆Cq Children with NB	∆Cq Healthy children	∆∆Cq
hsa-miR-875-5p	0.003	15.962	24.504	8.542
hsa-miR-221	0.008	6.103	13.110	7.007
hsa-miR-137	0.010	18.922	25.618	6.696
hsa-miR-29b	0.010	10.864	17.503	6.639
hsa-miR-202	0.011	17.226	23.715	6.489
hsa-miR-17	0.012	1.854	8.232	6.378
hsa-let-7d	0.015	5.870	11.955	6.084
hsa-miR-639	0.017	15.454	21.304	5.851
hsa-miR-320	0.017	5.863	11.700	5.837
hsa-miR-383	0.018	20.729	26.531	5.802
hsa-miR-617	0.019	21.831	27.524	5.693
hsa-miR-34a	0.019	9.973	15.664	5.691
hsa-miR-376b	0.019	22.075	27.761	5.686
hsa-miR-378	0.,021	8.586	14.178	5.591
hsa-miR-599	0.023	18.730	24.170	5.440
hsa-miR-944	0.025	18.807	24.136	5.328
hsa-miR-10a	0.026	10.927	16.204	5.277
hsa-miR-190b	0.028	11.943	17.102	5.159
hsa-miR-369-3p	0.031	18.744	23.760	5.016

### Expression of miR-17, miR-137 and miR-221 by NB primary tumor cells and BM-infiltrating NB cells

To check whether the increased expression of miR-17, miR-137 and miR-221 in BM resident cells from children with NB, as compared with healthy children, could be ascribed to their release from NB primary tumor cells and/or BM-infiltrating metastatic NB cells [20], the mean expression levels in all these tissues were compared (MiQe for qPCR in Supplementary Table 1, raw data in Supplementary Table 3 and 4, respectively).

**Figure 2:**
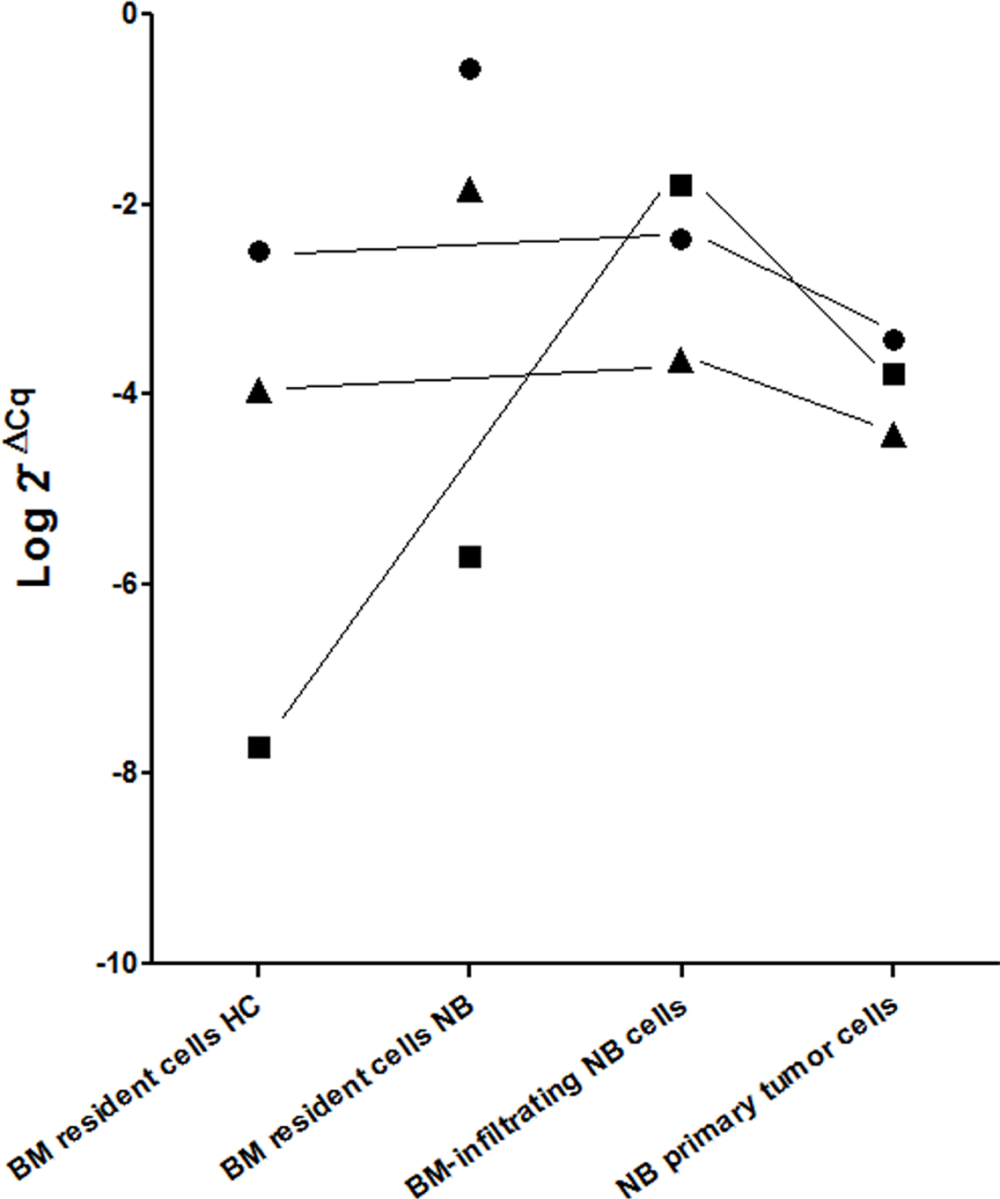
Mean expression levels (Log 2^–∆Cq^) of miR-17 (closed circle), miR-137 (closed square) and miR-221 (closed triangle) in BM resident cells from healthy children (HC) and from children with NB, in BM-infiltrating NB cells and in NB primary tumors MiR-17 and miR-221 expression levels in BM resident cells from healthy children were not significantly different from those of BM-infiltrating NB cells or primary tumor NB cells (*p* = ns). MiR-137 expression levels in BM resident cells from healthy children were significantly lower than those of BM-infiltrating NB cells or primary tumor NB cells (*p* = 0.0004 and *p* = 0.0002, respectively).

As shown in Figure 2, miR-17 and miR-221 were expressed at the same level by BM resident cells from healthy children, BM-infiltrating NB cells and NB primary tumor cells (*p* = ns), making unlikely a transfer from NB cells. Conversely, miR-137 was significantly more expressed by BM-infiltrating NB cells and NB primary tumor cells, as compared with BM resident cells from healthy children (Figure 2, *p* = 0.0036 and *p* = 0.0053, respectively), suggesting that miR-137 over-expression only in BM resident cells from children with metastatic NB may be dependent on its release by BM-infiltrating NB cells.

### Genes potentially targeted by miR-221 and under-expressed by BM resident cells from children with NB

Since only miR-221 over-expression occurred in all patients with NB regardless of presence of metastatic cells in the BM as it is the case of altered erythrocyte maturation [17], we extracted the lists of genes potentially targeted by miR-221 from three different miRNA databases, Targetscan, miRanda and PicTar. In fact, TargetScan predicts biological targets of miRNAs by searching for the presence of conserved 8mer, 7mer, and 6mer sites that match the seed region of each miRNA [21] (http://www.targetscan.org); PicTar uses an algorithm to provide details, such as 3′ UTR alignments with predicted sites and links to various public databases [22] (http://pictar.mdc-berlin.de), and miRanda prediction algorithm optimizes sequence complementarity using position-specific rules and relies on strict requirements of interspecies conservation [23] (http://www.microrna.org/microrna/home.do).

By merging the three target gene lists from the three databases, the unique potential miR-221 target genes were extracted (Supplementary Table 5, left column), and then matched to the list of genes under-expressed by BM-resident cells from children with NB, as compared with healthy children (Supplementary Table 5, right column). The latter list was derived from the GEO dataset GSE90689) [17] that were the most recent dataset available for this type of tissue.

The list of genes under-expressed by BM resident cells potentially targeted by miR-221, shown in Table 3, was then investigated for functional annotation (https://david.ncifcrf.gov/) [24] and pathway analysis (Ingenuity Systems Pathway Analysis, www.qiagenbioinformatics.com). None of the genes shown in Table 3 were involved in heme and porphyrin biosynthesis, whereas two genes, namely *BCL2L11* and *BNIP3L,* were related to mitochondria, where heme and porphyrin biosynthesis occurs.

**Table 3:** Genes potentially targeted by miR-221 and under-expressed in BM resident cells from children with NB, as compared with healthy children

Genes
ACOT1
ACOT2
ADIPOR1
AMMECR1
AP2A1
APEH
APOM
ARHGAP19
ATP1A1
ATP1B1
BCL11B
BCL2L11
BMP2K
BNIP3L
BPGM
C18orf56
C4B
CCDC47
CD2AP
CD40
CD99
CENPO
CLDN5
CMTM5
CTCF
CTSE
DCLRE1A
DCUN1D1
DLG7
E2F2
EIF3S1
EIF5A2
FAHD1
GBGT1
GPR137B
GSTT1
GUK1
HDAC4
HMG2L1
IFRD2
ITGB3
LHFPL2
MARCH8
MCM5
MST1
MYBL2
NAP1L5
NAPA
NGRN
NUTF2
PAIP1
PES1
PFDN6
PIGC
PLEKHF1
PPP1R8
PRPS1
PSMB5
QARS
SIGLECP3
SLC25A15
SLC25A37
SLC25A39
TIGD6
TIMP3
TMEM85
TMPRSS9
WDR40A
WDR89
WHSC1
WRN
WSB2
YWHAG
ZNF557

### Expression of *BCL2L11* and *BNIP3L* genes and their protein products by BM resident cells from children with NB as compared with healthy children

**Figure 3:**
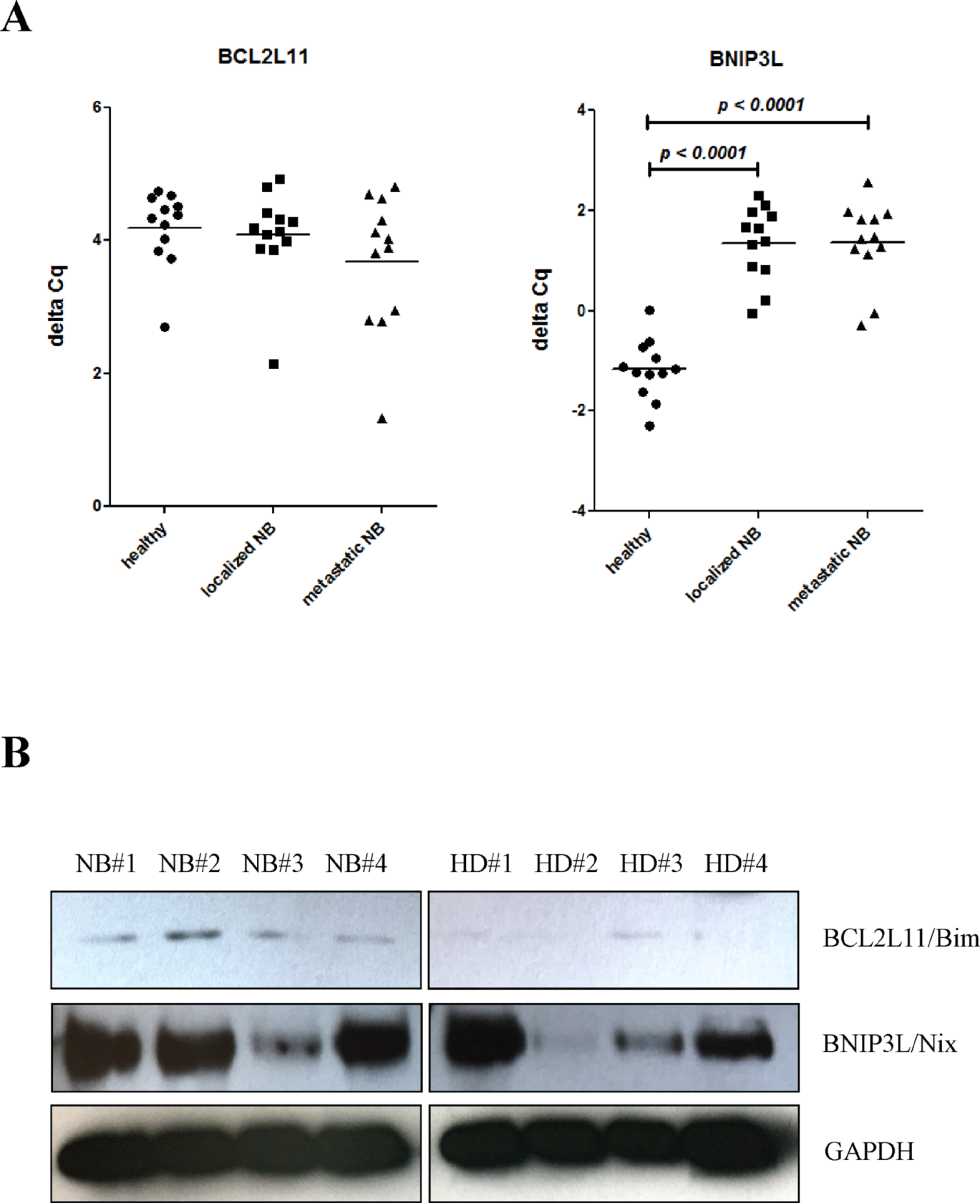
**(A)** Expression levels (Delta Cq) of *BCL2L11* and *BNIP3L* mRNA in BM resident cells from healthy children (closed circles) and from children with localized (closed squares) and metastatic (close triangles) NB. *N* = 12 for each group. **(B)** Western blot analysis of BCL2L11 and BNIP3L protein products, Bim and Nix, respectively, in BM resident cells from four children with NB (NB#1 to #4) and from four healthy children (HD#1 to #4).

We thus evaluated *BCL2L11* and *BNIP3L* gene expression levels in BM resident cells by RT-qPCR (Figure 3A) and their protein products by Western blot analysis (Figure 3B). *BCL2L11* gene expression and protein levels in BM resident cells from children with NB and healthy children were not dissimilar. Conversely, *BNIP3L* gene expression levels were confirmed to be significantly lower in BM resident cells from children with localized and metastatic NB, as compared with healthy children (Figure 3A). However, the level of *BNIP3L* protein product, Nix, was heterogeneous both in children with NB and healthy children (Figure 3B), without any significant reduction in children with NB.

## DISCUSSION

The transfer of miRNAs, key regulators of gene expression, is considered as one of the major causes leading to modification of physiological functions occurring in tissues distant from the primary tumor [2–4]. Therefore, we have investigated whether the selective erythrocyte impairment observed in children with NB, regardless of the presence of metastatic cells in the BM microenvironment [17], was mediated by a specific miRNA secreted by NB cells.

Our results did not support this hypothesis. First of all, the miRNA expression profile of BM resident cells from children with NB was not significantly different from that of healthy children. By considering the fold change we identified six miRNAs potentially over-expressed in NB by more than 150 fold, as compared with healthy children. Validation experiments performed in a larger number of samples from each group confirmed the differential expression for 50% of these miRNAs. The low percentage of validated miRNAs may be depended on the high heterogeneity of NB [6] or on the different platform used for the test and validation experiments. This finding was in line with other studies on miRNA expression in NB tumors [9, 10, 12–14, 20].

Three miRNAs, namely miR-17, miR-137 and miR-221, were over-expressed by BM resident cells from children with NB, as compared with healthy children. However, miR-17 was over-expressed only in children with localized NB and miR-137 only in children with metastatic NB. Whereas high level of miR-137 were likely due to close contact between BM-infiltrating NB cells and BM resident cells, the increased expression of miR-17 and miR-221 was unlikely to be a consequence of a transfer from NB primary tumor cells, because the levels in NB cells were similar to that of BM resident cells from healthy children.

Since the altered erythrocyte maturation, recently documented in children with NB [17], occurred in both localized and metastatic NB, we focused our attention on miR-221 that was significantly over-expressed by BM resident cells from all children with NB. All the genes potentially targeted by miR-221 were extracted from three public miRNA databases and compared to the full list of genes that were under-expressed in BM resident cells from children with NB, as compared with healthy children (GSE90689) [17]. The GSE90689 was the most recent dataset containing gene expression data from a total of 57 BM resident cells from both healthy children and children with NB. The older dataset, GSE25624 [19], contained data from a total of 40 samples. Recently, a dataset containing expression data of BM resident cells from children with NB in comparison with expression data of BM-infiltrating NB cells (GSE 94035) [25] became available, but the lack of expression data of BM resident cells from healthy children made it unsuitable to answer our question.

The genes under-expressed by BM resident cells from children with NB, as compared with healthy children, potentially targeted by miR-221 did not annotate in the pathway of heme and porphyrin synthesis. Two genes, *BCL2L11* and *BNIP3L*, expressed in mitochondria where heme and porphyrin synthesis occurs, were tested for gene and protein expression, but the results did not support a role for them in the impairment of late erythropoiesis. MiR-221 has been implicated in the regulation of early erythropoiesis [26], since its over-expression following lentiviral transfection in CD34^+^ precursors reduced the levels of its target gene *CKIT* and impaired stem cell activity. In our dataset, no difference in *CKIT* expression levels was found, confirming that early erythropoiesis was not impaired in children with NB.

In conclusion, our data did not support the hypothesis that selective erythrocyte maturation may be mediated by miRNAs released by primary NB tumor cells nor by miR-221. However, we cannot rule out the possibility that other miRNAs, not evaluated in the present study, may be involved in the process. Thus, we have made public (see Supplemental Tables) miRNA expression data of BM resident cells from children with NB and from healthy children described here, as well as miRNA expression data of NB primary tumors and BM-infiltrating NB cells that were published previously [20].

## MATERIALS AND METHODS

### Patients and controls

Patients included in the study were consecutively diagnosed with NB between January 2007 and December 2008. The main features of the patients are summarized in Table 1. Demographic, genetic, clinical and follow-up data were retrieved from the Italian NB Registry [27]. After diagnosis, patients were treated according to the European protocol suited for her/his risk category, dependent on age, stage and *MYCN* status [5].

As controls we used BM samples obtained from healthy siblings of leukemia patients admitted at the Gaslini Institute to undergo BM transplants.

Written consent for research use of samples and clinical data was obtained by the legal guardians. The study was approved by the Gaslini Institute Ethical Committee and all analyses were performed according to the Helsinki declaration.

### BM resident cells from NB patients and healthy children

BM resident cells were immune-selected from BM samples taken at diagnosis from 24 children with NB and from 12 healthy children, as previously described [19]. The procedure was applied to all BM samples to rule out the possibility that the procedure *per se* could influence the results. From each group of samples, i.e., from localized or metastatic NB and healthy children, 4 samples were randomly selected to perform complete miRNA profiling. All twelve samples of each group were used in the validation experiments.

### RNA extraction, miRNA profiling and validation

Total RNA and miRNA fractions were extracted from immune-selected BM resident cells using the miRNeasyMini kit (Qiagen, Hilden, Germany) according to manufacturer’s protocols. Quality of the RNA fractions was evaluated using the BioAnalyzer 2100 (Agilent Technologies, Santa Clara, CA, USA).

Forty ng of the miRNA fraction were reverse transcribed using the Megaplex RT Primers Human Pool A and B (Thermo Fisher, Milan, Italy). At the end of the reaction, each RT product was amplified with the Megaplex PreAmp Primers A and B for 25 cycles. Then, the amplification products of 4 samples from each group were loaded onto MicroRNA TaqMan Card A and B, respectively. Card amplifications were performed on ViiA7 equipment for 40 cycles (Thermo Fisher). Normalization of expression was made by using the mean expression value of U6 small RNA measured in each card [20].

Validation was performed by loading the amplification products in a 96 well plate in duplicates with the specific TaqMan^©^ human microRNA assays (Thermo Fisher: hsa-miR-17, catalog #002308; hsa-miR-137, catalog #01129; hsa-miR-202, catalog #002363; hsa-miR-221, catalog #000524; hsa-miR-29b, catalog #000413; hsa-miR-875-5p, catalog #002203; U6 snRNA, catalog #001973). Results were expressed as delta Cq by subtracting the Cq value obtained for U6 small RNA from the Cq value of each miRNA [28].

### Genes targeted by miR-221

The lists of genes potentially targeted by miR-221 were downloaded from three public miRNA databases:Targetscan(http://www.targetscan.org/vert_71/), miRANDA(http://www.microrna.org/microrna/home.do) and PicTar (http://pictar.mdc-berlin.de/). The three lists were then merged to obtain a comprehensive list of all the genes potentially targeted by miR-221. The unique gene list was then compared with the list of genes significantly under-expressed by BM resident cells from children with NB, as compared with healthy subjects [19], deposited in National Center for Biotechnology Information Gene Expression Omnibus (GEO, http://www.ncbi.nlm.nih.gov/geo/, accession GSE90689).

### Gene expression analysis of potential miRNA targets

One-hundred ng of total RNA from the 36 samples under study were reverse transcribed as described [19, 29], and then amplified in triplicate with the specific TaqMan^©^ human gene expression assays for *BCL2L11, BNIP3L* and *GAPDH* (Thermo Fisher, catalog# Hs00708019_s1, Hs00188949_m1 and 4333764F, respectively). Results were expressed as delta Cq by subtracting the Cq value obtained for GAPDH from the Cq value of each target gene [28].

### Western blot

Protein lysates of BM resident cells from four children with metastatic NB and four healthy children, all included in Table 1, were obtained using Cell Extraction Buffer (Thermo Fisher), following manufacturer’s protocol. Protein concentration was assessed using a BCA assay (Bio-Rad Laboratories, Segrate, Italy) and absorbance at 562 nm was measured using Infinite^®^ 200 PRO spectrometer (Tecan Italia Srl, Cernusco Sul Naviglio, Italy). Protein lysates (40 μg per lane) were resolved on SDS 10% polyacrylamide gels and were transferred to nitrocellulose membranes. The membranes were sequentially incubated with anti-Bim/BCL2L11 (mouse monoclonal, H-5) and anti-Nix/BNIP3L (mouse monoclonal, H-8), both from Santa Cruz Biotechnology Inc., Heidelberg, Germany, and with anti-GAPDH (rabbit monoclonal antibody from Cell Signaling Europe, Leiden, The Netherlands).

All primary antibodies were diluted at 1:200 in TBS 0.1% Tween (Sigma Aldrich, Milano, Italy) and 5% non-fat dry milk (BioRad), and membranes were incubated overnight at 4° C in constant agitation. Secondary reagents (HRP-conjugated horse anti-mouse or goat anti-rabbit antibodies (Cell signaling) were diluted following manufacturer’s protocol and membranes were incubated for 1h at RT in constant agitation. Immune complexes were visualized using Clarity ECL Western Blot Substrate (BioRad) according to the manufacturer’s instructions.

### Statistical analysis

Analysis of miRNA Cq values from high-throughput qPCR assays was conducted using the HTqPCR package [30] of Bioconductor [31], which runs on R statistical computing environment (http://www.R-project.org/). Difference in miRNA expression levels in BM resident cells from the three groups of samples were tested by performing Mann-Whitney rank test. Adjusting *p* values to face multiple comparison problems were performed according to the Benjamini-Hochberg procedure [32].

Differences in miRNA and gene expression levels evaluated by RT-qPCR in 96-well plates in all 36 samples were tested by the Mann-Whitney rank test using the Prism software (GraphPad Software Inc., La Jolla, CA, USA).

## SUPPLEMENTARY MATERIALS TABLES












