# Supplementary material for: miRNA expression profile of bone marrow resident cells from children with neuroblastoma is not significantly different from that of healthy children
Source: Oncotarget. 2018 Apr 10;9(27):19014–25. doi: 10.18632/oncotarget.24874 (PMC5922374; doi:10.18632/oncotarget.24874)
Supplement: Supplementary file 1 [file oncotarget-09-19014-s001.pdf]

miRNA expression profile of bone marrow resident cells from children with neuroblastoma is not significantly different from that of healthy children

SUPPLEMENTARY MATERIALS

Supplementary Table 1: MIQE checklist. See Supplementary_Table_1

Supplementary Table 2: RT-qPCR raw data of miRNA expression in BM resident cells from four healthy children, four children with localized NB and four children with metastatic NB. See Supplementary_Table_2

Supplementary Table 3: RT-qPCR raw data of miRNA expression in NB primary tumor cells from 12 children with metastatic NB. See Supplementary_Table_3

Supplementary Table 4: RT-qPCR raw data of miRNA expression in BM-infiltrating NB cells from 12 children with metastatic NB. See Supplementary_Table_4

Supplementary Table 5: List of unique genes potentially targeted by miR-221 extracted from Targetscan, PicTar and MiRanda databases and list of the genes under-expressed by BM resident cells from children with NB, as compared with healthy children. See Supplementary_Table_5
