# Supplementary material for: miRNA expression profile of bone marrow resident cells from children with neuroblastoma is not significantly different from that of healthy children
Source: Oncotarget. 2018 Apr 10;9(27):19014–25. doi: 10.18632/oncotarget.24874 (PMC5922374; doi:10.18632/oncotarget.24874)
Supplement: Supplementary file 2 [file oncotarget-09-19014-s002.docx]

| **Supplementary Table 1: MIQE checklist** | |  |  |
| --- | --- | --- | --- |
|  | |  |  |
| **ITEM TO CHECK** | **IMPORTANCE** | | **CHECKLIST** |
| **EXPERIMENTAL DESIGN** | |  |  |
| Definition of experimental and control groups | | **E** | The complete miRNA profiling was determined for:  **A:** BM-infiltrating NB cells, **B:** NB primary tumor cells, **C:** BM resident cells from children with localized NB, **D:** BM resident cells from children with metastatic NB, **E:** BM resident cells from healthy children |
| Number within each group | | **E** | For **groups A** and **B:** 12 samples; for **groups** **C, D** and **E:** 4 samples |
| Assay carried out by core lab or investigator's lab? | | **D** | Assay carried out by investigator's lab |
| Acknowledgement of authors' contributions | | **D** | Assay were performed by MVC |
| **SAMPLE** | |  |  |
| Description | | **E** | **A:** 22 BM-infiltrating GD2+ cells were immune-selected at diagnosis, **B:** 22 frozen primary tumor specimens from our bio-bank containing more than 80% NB cells taken at diagnosis from stage M NB patients aged more than 12 months at diagnosis. From **A and B**, 12 samples were randomly selected to perform miRNA profiling. (Stigliani S, et al. Oncotarget. 2015;6(15):13295-308).  **C** and **D:** BM resident cells were immune-selected from BM samples taken at diagnosis from 24 children with NB, **E:** BM resident cells from 12 healthy children (Scaruffi P, et al. Pediatr Blood Cancer. 2012;59(1):44-51). From each group 4 samples were randomly selected to perform miRNA profiling. |
| Volume/mass of sample processed | | **D** | **B:** for tumor samples volume was not measured, **A, C, D** and **E:** volume of BM samples ranged between 3 and 8 ml (average 5 ml) |
| Microdissection or macrodissection | | **E** | **B:** Tumor specimens containing more than 80% NB cells, **A, C, D** and **E:** not applicable |
| Processing procedure | | **E** | **B:** samples were extracted from frozen primary tumors containing more than 80% NB cells. **A, C, D and E:** After erythrocytes lysis, cells were incubated for 30 minutes at 4°C with antiGD2 mAb in the presence of human Fc blocking reagent. After washing, the cell pellet was incubated for 15 minutes at 4°C with goat anti-mouse IgG2a microbeads (Miltenyi Biotec). Cells were then washed twice, suspended in 500 μl of sterile ice-cold PBS and loaded onto a MS column (Miltenyi). Immunomagnetic separation was performed according to the manufacturer’s instructions to obtain GD2+ and GD2- fractions |
| If frozen - how and how quickly? | | **E** | **B:** samples were extracted from frozen specimens obtained at diagnosis from NB patients. After surgery and histo-pathological evaluation, the tumor specimen were immediately frozen in liquid nitrogen. **A, C, D** and **E:** fresh samples |
| If fixed - with what, how quickly? | | **E** | Not fixed |
| Sample storage conditions and duration (especially for FFPE samples) | | **E** | Frozen samples were stored at -80°C until RNA extraction. |
| **NUCLEIC ACID EXTRACTION** | |  |  |
| Procedure and/or instrumentation | | **E** | Total RNA and miRNA fractions were extracted using the miRNeasyMini kit (Qiagen, Hilden, Germany) according to the manufacturer’s procedures |
| Name of kit and details of any modifications | | **E** | miRNeasyMini kit (Cat No. 74104) |
| Source of additional reagents used | | **D** | None |
| Details of DNase or RNAse treatment | | **E** | On column RNase-free DNase I treatment |
| Contamination assessment (DNA or RNA) | | **E** | Not applicable |
| Nucleic acid quantification | | **E** | Quality of the RNA fractions were evaluated in the BioAnalyzer 2100 system (Agilent Technologies) using Agilent RNA 6000 Nano Kit and Agilent Small RNA kit |
| Instrument and method | | **E** | BioAnalyzer 2100 system (Agilent Technologies) according to standard protocol |
| Purity (A260/A280) | | **D** | According to Nanodrop measurement total RNA purity (A260/A280) ranged between 1.8 and 2.0 |
| Yield | | **D** | According to Nanodrop measurement total RNA yield was on average about 200 ng/μl (range 20 – 900) |
| RNA integrity method/instrument | | **E** | RIN obtained from BioAnalyzer 2100 system |
| RIN/RQI or Cq of 3' and 5' transcripts | | **E** | Only samples with RIN ≥ 7 were included in the study |
| Electrophoresis traces | | **D** | Traces obtained with BioAnalyzer 2100 system |
| Inhibition testing (Cq dilutions, spike or other) | | **E** | Not performed |
| **REVERSE TRANSCRIPTION** | |  |  |
| Complete reaction conditions | | **E** | The miRNA fraction of each sample was subjected to stem-loop RT-qPCR amplification, as described (Mestdagh P,et al. Nucleic Acids Res. 2008;36(21):e143). |
| Amount of RNA and reaction volume | | **E** | 30 ng of the miRNA fraction were reverse transcribed using the Megaplex RT Primers Human Pool A and B, in a final volume of 7,5 μl |
| Priming oligonucleotide (if using GSP) and concentration | | **E** | Primers Human Pool A and B provided from Thermo Fisher Scientific, USA. |
| Reverse transcriptase and concentration | | **E** | **RT:** Megaplex™ RT Primers (10✕) 0.80; dNTPs with dTTP (100 mM) 0.20; MultiScribe™ Reverse Transcriptase (50 U/μL) 1.50;10✕ RT Buffer 0.80;MgCl2 (25 mM) 0.90; RNase Inhibitor (20 U/μL) 0.10; Nuclease-free water 0.20  At the end of the reaction, each RT product was amplified with the Megaplex PreAmp Primers A and B for 25 cycles with PreAmp: TaqMan® PreAmp Master Mix (2✕) 12.5; Megaplex™ PreAmp Primers (10✕) 2.5; Nuclease-free water 7.5 |
| Temperature and time | | **E** | **RT**: (16 °C 2 min, 42 °C 1 min, 50 °C 1 sec) x 40 Cycles, then 85 °C 5 min and 4 °C.  **PreAmp:** 95 °C 10 min; 55 °C 2 min; 72 °C 2 min; (95 °C 15 sec; 60 °C 4 min) x 12 Cycles, then 99.9 °C 10 min and 4 °C |
| Manufacturer of reagents and catalogue numbers | | **D** | All reagents were purchased from Applied Biosystems now Thermo Fisher Scientific (Megaplex™ RT Human Pool A 4399966 , Megaplex™ RT Human Pool B 4399968; Megaplex™ PreAmp Primers, Human Pool A 4399233 Megaplex™ PreAmp Primers, Human Pool B 4399201) |
| Cqs with and without RT | | **D*** | Not applicable |
| Storage conditions of cDNA | | **D** | -20°C |
| **qPCR TARGET INFORMATION** | |  |  |
| Gene symbol | | **E** | Comprehensive coverage of Sanger miRBase v10 is enabled across the two-card set of TaqMan® MicroRNA Arrays (Arrays A and B) for a total of 671 unique assays specific to human miRNAs (see raw data submission) |
| Sequence accession number | | **E** | See raw data submission with official name for each miRNA |
| Location of amplicon | | **D** | Not applicable |
| Amplicon length | | **E** | Not applicable |
| In silico specificity screen (BLAST, etc) | | **E** | Not applicable |
| Pseudogenes, retropseudogenes or other homologs? | | **D** | Not applicable |
| Sequence alignment | | **D** | Not applicable |
| Secondary structure analysis of amplicon | | **D** | Not applicable |
| Location of each primer by exon or intron (if applicable) | | **E** | Not applicable |
| What splice variants are targeted? | | **E** | Not applicable |
| **qPCR OLIGONUCLEOTIDES** | |  |  |
| Primer sequences | | **E** | Commercial from Applied Biosystems now Thermo Fisher Scientific |
| RTPrimerDB Identification Number | | **D** | Not applicable |
| Probe sequences | | **D**** | Commercial |
| Location and identity of any modifications | | **E** | Not applicable |
| Manufacturer of oligonucleotides | | **D** | Applied Biosystems now Thermo Fisher Scientific |
| Purification method | | **D** |  |
| **qPCR PROTOCOL** | |  |  |
| Complete reaction conditions | | **E** | The amplification products were loaded onto MicroRNA TaqMan Card A and B, respectively. Card amplifications were performed on ViiA7 equipment for 40 cycles. |
| Reaction volume and amount of cDNA/DNA | | **E** | TaqMan Universal PCR Master Mix, No AmpErase® UNG, 2✕450 μl; Diluted PreAmp product 9 μl; Nuclease-free water 441 μl |
| Primer, (probe), Mg++ and dNTP concentrations | | **E** | primer and probe within the 384 well card |
| Polymerase identity and concentration | | **E** | AmpliTaq Gold® DNA Polymerase (Thermo Fisher Scientific PN 4324018) |
| Buffer/kit identity and manufacturer | | **E** | (Thermo Fisher Scientific PN 4324018) |
| Exact chemical constitution of the buffer | | **D** | Not available |
| Additives (SYBR Green I, DMSO, etc.) | | **E** | None |
| Manufacturer of plates/tubes and catalog number | | **D** | TaqMan® Human MicroRNA Array A 4398965; TaqMan® Human MicroRNA Array B 4398966 |
| Complete thermocycling parameters | | **E** | 95 °C 10 min; then 95 °C 15 sec; 60 °C 1 min) x 40 Cycles |
| Reaction setup (manual/robotic) | | **D** | Manual |
| Manufacturer of qPCR instrument | | **E** | ViiA 7 Real-Time PCR System (Thermo Fisher Scientific) |
| **qPCR VALIDATION** | |  |  |
| Evidence of optimisation (from gradients) | | **D** | Since it is a commercial assay we did not perform any optimization |
| Specificity (gel, sequence, melt, or digest) | | **E** | Not applicable |
| For SYBR Green I, Cq of the NTC | | **E** | No SYBR Green |
| Calibration curves with slope and y-intercept | | **E** | Not applicable |
| PCR efficiency calculated from slope | | **E** | Not applicable |
| Confidence interval for PCR efficiency or standard error | | **D** | Not applicable |
| r2 of calibration curve | | **E** | Not applicable |
| Linear dynamic range | | **E** | Not applicable |
| Cq variation at limit of detection | | **E** | Not applicable |
| Confidence intervals throughout range | | **D** | Not applicable |
| Evidence for limit of detection | | **E** | Not applicable |
| If multiplex, efficiency and LOD of each assay | | **E** | Each TaqMan assay was performed in a separate well of the 384 well plate |
| **DATA ANALYSIS** | |  |  |
| qPCR analysis program (source, version) | | **E** | qPCR analysis ViiA 7 Real-Time PCR System (Thermo Fisher Scientific) version 1.1 |
| method of Cq determination | | **E** | Fluorescence |
| Outlier identification and disposition | | **E** | ViiA 7 Real-Time PCR System (Thermo Fisher Scientific) Software version 1.1 |
| Results of NTCs | | **E** | Not applicable |
| Justification of number and choice of reference genes | | **E** | U6 was chosen for normalization because it was very stable across all the different samples |
| Description of normalization method | | **E** | Delta Cq according to Livak KJ1, Schmittgen TD. Analysis of relative gene expression data using real-time quantitative PCR and the 2(-Delta Delta C(T)) Method. Methods. 2001 Dec;25(4):402-8. |
| Number and concordance of biological replicates | | **D** | Replicates included into Cards A and B were always concordant |
| Number and stage (RT or qPCR) of technical replicates | | **E** | No technical replicates |
| Repeatability (intra-assay variation) | | **E** | Not applicable |
| Reproducibility (inter-assay variation, CV) | | **D** | Not applicable |
| Power analysis | | **D** | Not applicable |
| Statistical methods for result significance | | **E** | Analysis of miRNA expression Cq values from high-throughput qPCR assays was conducted using the HTqPCR package of Bioconductor, which runs on R statistical computing environment. |
| Software (source, version) | | **E** | http://www.R-project.org/ |
| Cq or raw data submission using RDML | | **D** | Yes |
|  | |  |  |
