# Supplementary material for: miRNA expression profile of bone marrow resident cells from children with neuroblastoma is not significantly different from that of healthy children
Source: Oncotarget. 2018 Apr 10;9(27):19014–25. doi: 10.18632/oncotarget.24874 (PMC5922374; doi:10.18632/oncotarget.24874)
Supplement: Supplementary file 4 [file oncotarget-09-19014-s004.docx]

**Supplementary Table 3:** **RT-qPCR raw data of miRNA expression in NB primary tumor cells from 12 children with metastatic NB**

| **miRNA** | **NB primary tumor cells 1** | **NB primary tumor cells 2** | **NB primary tumor cells 3** | **NB primary tumor cells 4** | **NB primary tumor cells 5** | **NB primary tumor cells 6** | **NB primary tumor cells 7** | **NB primary tumor cells 8** | **NB primary tumor cells 9** | **NB primary tumor cells 10** | **NB primary tumor cells 11** | **NB primary tumor cells 12** |
| --- | --- | --- | --- | --- | --- | --- | --- | --- | --- | --- | --- | --- |
| ath-miR159a | 40.00 | 25.96 | 31.73 | 34.95 | 35.38 | 35.26 | 32.48 | 31.08 | 35.17 | 32.20 | 37.03 | 34.95 |
| ath-miR159a | 40.00 | 40.00 | 40.00 | 40.00 | 40.00 | 30.73 | 40.00 | 40.00 | 40.00 | 33.70 | 40.00 | 40.00 |
| hsa-let-7a | 25.53 | 24.59 | 27.24 | 23.38 | 23.10 | 23.85 | 21.45 | 23.41 | 25.96 | 22.27 | 25.72 | 22.86 |
| hsa-let-7a* | 40.00 | 40.00 | 40.00 | 40.00 | 38.52 | 30.50 | 36.75 | 40.00 | 40.00 | 34.43 | 40.00 | 40.00 |
| hsa-let-7b | 20.13 | 20.80 | 16.15 | 20.58 | 20.08 | 21.02 | 20.42 | 19.48 | 21.55 | 20.54 | 21.40 | 19.92 |
| hsa-let-7b* | 40.00 | 40.00 | 37.17 | 40.00 | 40.00 | 40.00 | 40.00 | 38.38 | 40.00 | 37.26 | 39.99 | 40.00 |
| hsa-let-7c | 24.21 | 22.72 | 23.96 | 22.28 | 21.20 | 23.19 | 21.82 | 22.72 | 25.76 | 22.27 | 23.94 | 20.81 |
| hsa-let-7d | 25.06 | 23.71 | 25.57 | 23.90 | 23.69 | 24.11 | 22.14 | 23.11 | 24.58 | 23.23 | 24.81 | 23.16 |
| hsa-let-7d* | 40.00 | 40.00 | 40.00 | 40.00 | 37.65 | 38.60 | 37.93 | 37.06 | 40.00 | 38.56 | 40.00 | 40.00 |
| hsa-let-7e | 22.18 | 21.12 | 23.71 | 19.98 | 19.97 | 20.83 | 19.26 | 20.79 | 22.56 | 19.77 | 22.25 | 19.77 |
| hsa-let-7e* | 40.00 | 33.73 | 34.17 | 32.03 | 33.19 | 33.43 | 31.53 | 32.83 | 36.41 | 34.63 | 40.00 | 35.36 |
| hsa-let-7f | 32.38 | 28.77 | 32.69 | 31.39 | 31.27 | 32.86 | 25.70 | 28.96 | 34.79 | 28.28 | 31.48 | 30.56 |
| hsa-let-7f-1* | 40.00 | 40.00 | 40.00 | 40.00 | 40.00 | 40.00 | 40.00 | 40.00 | 40.00 | 38.89 | 40.00 | 40.00 |
| hsa-let-7f-2* | 40.00 | 40.00 | 34.48 | 40.00 | 40.00 | 40.00 | 37.74 | 40.00 | 40.00 | 33.48 | 40.00 | 40.00 |
| hsa-let-7g | 26.57 | 22.18 | 26.57 | 23.75 | 23.32 | 24.76 | 20.74 | 24.13 | 25.69 | 23.22 | 25.52 | 23.30 |
| hsa-let-7g* | 33.92 | 35.89 | 36.11 | 40.00 | 34.93 | 33.99 | 29.90 | 40.00 | 34.91 | 29.98 | 37.27 | 40.00 |
| hsa-let-7i* | 40.00 | 37.76 | 40.00 | 40.00 | 36.51 | 40.00 | 32.68 | 40.00 | 36.65 | 40.00 | 40.00 | 40.00 |
| hsa-miR-1 | 40.00 | 39.50 | 40.00 | 40.00 | 40.00 | 40.00 | 31.15 | 40.00 | 33.59 | 29.47 | 37.05 | 40.00 |
| hsa-miR-100 | 24.51 | 25.09 | 25.81 | 26.21 | 24.98 | 24.78 | 21.72 | 24.84 | 26.92 | 25.44 | 26.98 | 26.15 |
| hsa-miR-100* | 31.43 | 40.00 | 40.00 | 32.74 | 38.28 | 28.77 | 28.42 | 35.85 | 40.00 | 28.27 | 40.00 | 39.60 |
| hsa-miR-101 | 29.80 | 29.04 | 32.53 | 32.75 | 40.00 | 31.21 | 27.54 | 30.13 | 30.44 | 29.35 | 31.34 | 30.15 |
| hsa-miR-101* | 40.00 | 40.00 | 40.00 | 39.05 | 40.00 | 40.00 | 40.00 | 40.00 | 40.00 | 30.43 | 40.00 | 40.00 |
| hsa-miR-103 | 26.90 | 23.42 | 28.37 | 23.55 | 23.51 | 26.04 | 22.08 | 24.36 | 26.60 | 23.43 | 25.63 | 22.87 |
| hsa-miR-105 | 29.81 | 24.67 | 36.79 | 25.05 | 27.01 | 29.22 | 26.71 | 30.26 | 40.00 | 27.55 | 30.40 | 24.82 |
| hsa-miR-105* | 40.00 | 40.00 | 40.00 | 36.27 | 40.00 | 40.00 | 40.00 | 40.00 | 40.00 | 40.00 | 40.00 | 40.00 |
| hsa-miR-106a | 22.75 | 20.18 | 24.19 | 20.94 | 20.64 | 21.53 | 17.28 | 21.41 | 23.84 | 20.29 | 20.87 | 19.08 |
| hsa-miR-106a* | 40.00 | 40.00 | 40.00 | 40.00 | 40.00 | 40.00 | 40.00 | 40.00 | 40.00 | 40.00 | 40.00 | 40.00 |
| hsa-miR-106b | 25.53 | 21.98 | 27.26 | 23.39 | 23.75 | 24.41 | 20.19 | 23.89 | 25.61 | 23.32 | 23.99 | 21.65 |
| hsa-miR-106b* | 25.49 | 21.03 | 27.21 | 24.52 | 24.14 | 22.85 | 20.91 | 25.95 | 28.36 | 24.68 | 24.29 | 21.43 |
| hsa-miR-107 | 33.04 | 27.64 | 34.57 | 29.09 | 29.27 | 30.17 | 27.46 | 29.37 | 32.24 | 28.16 | 31.02 | 27.54 |
| hsa-miR-10a | 28.16 | 28.13 | 28.53 | 29.15 | 30.08 | 29.08 | 24.36 | 26.57 | 28.19 | 27.49 | 28.97 | 29.87 |
| hsa-miR-10a* | 40.00 | 33.06 | 40.00 | 32.58 | 35.33 | 33.24 | 29.54 | 39.91 | 40.00 | 38.74 | 40.00 | 40.00 |
| hsa-miR-10b | 24.48 | 25.61 | 27.59 | 26.73 | 26.44 | 27.81 | 21.26 | 27.60 | 31.45 | 25.02 | 28.27 | 25.42 |
| hsa-miR-10b* | 29.40 | 26.78 | 38.13 | 28.06 | 27.00 | 27.76 | 23.67 | 29.70 | 31.64 | 27.62 | 28.92 | 28.18 |
| hsa-miR-122 | 32.48 | 40.00 | 35.23 | 31.27 | 29.13 | 33.91 | 34.70 | 36.71 | 33.73 | 30.98 | 35.63 | 36.11 |
| hsa-miR-122* | 40.00 | 29.52 | 40.00 | 40.00 | 40.00 | 29.03 | 38.37 | 40.00 | 40.00 | 34.64 | 40.00 | 40.00 |
| hsa-miR-124 | 26.38 | 25.54 | 29.18 | 26.04 | 26.98 | 26.58 | 25.76 | 27.85 | 26.49 | 26.78 | 27.90 | 24.59 |
| hsa-miR-124* | 40.00 | 40.00 | 40.00 | 35.66 | 40.00 | 33.33 | 32.60 | 40.00 | 40.00 | 32.98 | 40.00 | 40.00 |
| hsa-miR-125a-3p | 30.59 | 31.65 | 31.00 | 30.07 | 30.38 | 32.23 | 30.87 | 30.72 | 31.32 | 29.72 | 30.72 | 29.41 |
| hsa-miR-125a-5p | 25.05 | 24.37 | 26.05 | 23.47 | 23.27 | 25.63 | 23.72 | 24.54 | 27.25 | 22.38 | 25.12 | 23.07 |
| hsa-miR-125b | 19.55 | 19.53 | 20.37 | 19.32 | 18.92 | 20.57 | 20.24 | 19.66 | 22.33 | 18.92 | 21.54 | 18.69 |
| hsa-miR-125b-1* | 34.40 | 32.87 | 34.10 | 31.15 | 30.98 | 31.84 | 30.73 | 32.21 | 40.00 | 31.21 | 31.26 | 32.63 |
| hsa-miR-125b-2* | 33.91 | 32.48 | 37.72 | 35.65 | 31.13 | 29.06 | 32.39 | 37.77 | 40.00 | 37.67 | 34.55 | 33.76 |
| hsa-miR-126 | 23.79 | 22.95 | 26.24 | 26.83 | 24.06 | 24.87 | 18.51 | 22.06 | 25.78 | 22.86 | 25.25 | 25.67 |
| hsa-miR-126* | 26.98 | 25.87 | 29.64 | 30.81 | 27.86 | 27.47 | 22.16 | 26.13 | 29.27 | 26.42 | 28.94 | 31.85 |
| hsa-miR-127-3p | 19.69 | 20.16 | 22.78 | 18.34 | 25.12 | 19.73 | 20.73 | 20.08 | 23.62 | 23.81 | 22.10 | 19.05 |
| hsa-miR-127-5p | 40.00 | 40.00 | 40.00 | 34.63 | 40.00 | 31.86 | 38.43 | 34.94 | 40.00 | 35.55 | 40.00 | 33.14 |
| hsa-miR-128 | 30.18 | 26.48 | 33.63 | 27.33 | 26.23 | 27.92 | 25.11 | 29.01 | 30.01 | 26.76 | 29.38 | 25.50 |
| hsa-miR-129-3p | 31.07 | 27.15 | 34.84 | 23.82 | 26.71 | 26.42 | 27.36 | 31.01 | 32.78 | 23.08 | 29.46 | 23.05 |
| hsa-miR-129-5p | 40.00 | 30.27 | 40.00 | 27.05 | 30.98 | 29.97 | 40.00 | 35.63 | 40.00 | 26.60 | 35.78 | 26.29 |
| hsa-miR-130a | 29.56 | 26.50 | 31.68 | 29.87 | 27.92 | 29.46 | 23.48 | 27.70 | 31.39 | 27.67 | 29.44 | 27.09 |
| hsa-miR-130a* | 34.41 | 19.60 | 40.00 | 40.00 | 40.00 | 36.33 | 30.21 | 40.00 | 40.00 | 40.00 | 40.00 | 40.00 |
| hsa-miR-130b | 29.57 | 24.96 | 29.57 | 24.54 | 25.84 | 26.96 | 21.92 | 29.00 | 29.53 | 25.62 | 26.22 | 25.02 |
| hsa-miR-130b* | 30.48 | 25.75 | 35.42 | 27.65 | 28.79 | 26.88 | 25.93 | 35.55 | 33.07 | 27.98 | 28.92 | 28.63 |
| hsa-miR-132 | 23.44 | 23.07 | 26.58 | 23.71 | 22.81 | 22.61 | 21.62 | 23.62 | 24.14 | 22.91 | 24.53 | 23.28 |
| hsa-miR-132* | 40.00 | 40.00 | 40.00 | 40.00 | 40.00 | 33.55 | 40.00 | 40.00 | 40.00 | 32.50 | 40.00 | 40.00 |
| hsa-miR-133a | 29.07 | 31.05 | 29.76 | 29.64 | 27.56 | 28.41 | 26.12 | 26.18 | 27.96 | 24.79 | 27.99 | 29.58 |
| hsa-miR-133b | 30.42 | 37.75 | 37.18 | 36.74 | 36.24 | 35.03 | 31.62 | 30.40 | 38.37 | 29.01 | 33.87 | 40.00 |
| hsa-miR-134 | 27.17 | 27.17 | 30.48 | 24.33 | 31.84 | 25.92 | 27.55 | 26.73 | 31.66 | 30.51 | 29.00 | 26.34 |
| hsa-miR-135a | 32.44 | 28.15 | 15.19 | 30.74 | 30.70 | 30.48 | 25.41 | 30.10 | 32.87 | 30.01 | 30.59 | 30.01 |
| hsa-miR-135a* | 18.34 | 18.14 | 18.56 | 18.62 | 18.36 | 18.29 | 18.04 | 18.51 | 18.76 | 18.42 | 18.40 | 19.01 |
| hsa-miR-135b | 40.00 | 27.49 | 32.85 | 31.71 | 30.93 | 27.86 | 25.13 | 29.63 | 40.00 | 29.64 | 29.47 | 27.94 |
| hsa-miR-135b* | 40.00 | 40.00 | 40.00 | 40.00 | 40.00 | 31.24 | 30.54 | 40.00 | 40.00 | 34.60 | 40.00 | 40.00 |
| hsa-miR-136 | 36.04 | 32.84 | 37.23 | 34.13 | 40.00 | 37.01 | 37.42 | 35.17 | 40.00 | 40.00 | 40.00 | 39.45 |
| hsa-miR-136* | 30.03 | 28.40 | 33.18 | 30.63 | 40.00 | 29.99 | 28.65 | 29.23 | 33.43 | 30.14 | 40.00 | 29.75 |
| hsa-miR-137 | 30.44 | 27.34 | 30.45 | 29.02 | 29.53 | 30.35 | 26.35 | 27.98 | 39.97 | 26.13 | 30.94 | 30.24 |
| hsa-miR-138 | 22.11 | 22.73 | 25.24 | 19.14 | 19.07 | 22.54 | 21.82 | 24.79 | 30.12 | 19.80 | 25.30 | 20.49 |
| hsa-miR-138-1* | 25.95 | 25.78 | 26.47 | 26.21 | 25.19 | 25.21 | 25.42 | 26.51 | 26.47 | 25.75 | 25.98 | 26.77 |
| hsa-miR-138-2* | 40.00 | 36.94 | 40.00 | 36.92 | 31.19 | 33.10 | 39.69 | 40.00 | 40.00 | 29.82 | 40.00 | 35.32 |
| hsa-miR-139-3p | 29.14 | 33.48 | 29.66 | 25.44 | 25.94 | 26.05 | 26.97 | 25.78 | 37.61 | 26.30 | 26.62 | 24.13 |
| hsa-miR-139-5p | 21.39 | 22.26 | 23.35 | 20.90 | 21.19 | 20.07 | 20.91 | 20.41 | 25.66 | 20.48 | 23.00 | 19.12 |
| hsa-miR-140-3p | 28.81 | 28.66 | 29.85 | 28.63 | 27.92 | 29.25 | 26.34 | 27.91 | 30.18 | 28.12 | 28.36 | 27.76 |
| hsa-miR-140-5p | 29.39 | 27.55 | 30.91 | 29.56 | 28.65 | 28.96 | 24.49 | 27.29 | 30.06 | 27.64 | 33.38 | 28.87 |
| hsa-miR-141 | 38.92 | 32.68 | 40.00 | 32.81 | 40.00 | 40.00 | 32.68 | 33.50 | 40.00 | 40.00 | 39.52 | 40.00 |
| hsa-miR-141* | 40.00 | 40.00 | 40.00 | 40.00 | 37.17 | 35.78 | 36.12 | 40.00 | 40.00 | 36.92 | 35.33 | 40.00 |
| hsa-miR-142-3p | 26.29 | 25.04 | 28.66 | 30.39 | 28.85 | 29.24 | 21.54 | 22.80 | 24.42 | 27.39 | 26.92 | 30.60 |
| hsa-miR-142-5p | 39.72 | 31.96 | 40.00 | 38.60 | 40.00 | 32.41 | 29.71 | 31.33 | 34.14 | 35.34 | 40.00 | 40.00 |
| hsa-miR-143 | 26.40 | 25.35 | 28.23 | 27.00 | 24.99 | 27.01 | 22.53 | 24.10 | 25.99 | 24.36 | 27.47 | 26.63 |
| hsa-miR-143* | 40.00 | 40.00 | 40.00 | 40.00 | 40.00 | 35.28 | 37.60 | 40.00 | 40.00 | 40.00 | 40.00 | 40.00 |
| hsa-miR-144* | 30.62 | 29.06 | 40.00 | 32.07 | 31.68 | 31.21 | 26.64 | 30.11 | 40.00 | 32.64 | 28.20 | 40.00 |
| hsa-miR-145 | 18.28 | 19.76 | 19.29 | 19.34 | 18.87 | 19.90 | 17.74 | 18.41 | 18.91 | 17.80 | 21.22 | 19.96 |
| hsa-miR-145* | 34.24 | 30.15 | 36.42 | 31.93 | 30.12 | 30.20 | 26.64 | 31.43 | 31.44 | 30.49 | 32.62 | 35.37 |
| hsa-miR-146a | 23.29 | 23.23 | 24.82 | 27.56 | 25.07 | 24.46 | 19.94 | 21.67 | 23.25 | 26.06 | 27.48 | 26.01 |
| hsa-miR-146a* | 40.00 | 27.49 | 40.00 | 40.00 | 40.00 | 27.76 | 34.09 | 40.00 | 40.00 | 40.00 | 40.00 | 40.00 |
| hsa-miR-146b-3p | 27.68 | 28.25 | 29.47 | 32.11 | 29.78 | 28.56 | 27.00 | 26.68 | 27.82 | 33.39 | 29.55 | 31.84 |
| hsa-miR-146b-5p | 24.91 | 24.94 | 28.06 | 27.97 | 25.97 | 26.35 | 20.61 | 23.55 | 24.26 | 26.59 | 26.51 | 28.17 |
| hsa-miR-147 | 34.25 | 40.00 | 40.00 | 40.00 | 40.00 | 40.00 | 40.00 | 40.00 | 40.00 | 40.00 | 32.97 | 40.00 |
| hsa-miR-147b | 40.00 | 36.06 | 40.00 | 40.00 | 40.00 | 40.00 | 40.00 | 40.00 | 31.87 | 40.00 | 40.00 | 40.00 |
| hsa-miR-148a | 28.28 | 26.79 | 35.04 | 31.15 | 27.43 | 28.26 | 24.32 | 26.99 | 29.64 | 27.91 | 28.85 | 26.20 |
| hsa-miR-148a* | 40.00 | 40.00 | 40.00 | 40.00 | 40.00 | 40.00 | 40.00 | 40.00 | 40.00 | 40.00 | 40.00 | 40.00 |
| hsa-miR-148b | 32.63 | 28.59 | 32.89 | 30.38 | 28.76 | 29.84 | 28.40 | 28.89 | 32.76 | 28.60 | 33.29 | 29.08 |
| hsa-miR-148b* | 32.43 | 29.25 | 33.13 | 35.46 | 29.94 | 26.82 | 27.60 | 36.78 | 32.16 | 24.78 | 33.35 | 40.00 |
| hsa-miR-149 | 19.51 | 19.20 | 21.84 | 18.29 | 17.87 | 19.39 | 19.50 | 21.41 | 28.34 | 18.68 | 22.72 | 16.95 |
| hsa-miR-149* | 27.47 | 27.02 | 28.51 | 28.08 | 27.99 | 27.93 | 24.59 | 27.16 | 28.96 | 27.75 | 27.27 | 27.87 |
| hsa-miR-150 | 21.38 | 20.88 | 23.32 | 25.57 | 21.44 | 19.99 | 15.76 | 19.79 | 21.94 | 25.10 | 23.06 | 22.44 |
| hsa-miR-151-3p | 19.53 | 19.23 | 19.58 | 20.17 | 19.50 | 19.29 | 19.06 | 20.37 | 20.22 | 19.71 | 19.91 | 21.29 |
| hsa-miR-152 | 26.32 | 25.54 | 27.98 | 25.42 | 26.14 | 28.73 | 24.41 | 26.21 | 26.89 | 24.85 | 28.18 | 25.69 |
| hsa-miR-153 | 40.00 | 31.99 | 40.00 | 40.00 | 40.00 | 36.30 | 36.69 | 40.00 | 40.00 | 34.23 | 40.00 | 33.65 |
| hsa-miR-154 | 40.00 | 40.00 | 40.00 | 39.43 | 40.00 | 36.48 | 32.39 | 35.44 | 33.84 | 40.00 | 40.00 | 39.57 |
| hsa-miR-154* | 40.00 | 31.68 | 40.00 | 32.56 | 40.00 | 36.12 | 32.17 | 37.83 | 40.00 | 33.77 | 40.00 | 32.62 |
| hsa-miR-155 | 20.32 | 18.93 | 18.87 | 18.62 | 19.14 | 18.50 | 18.74 | 19.35 | 18.69 | 18.15 | 17.85 | 19.02 |
| hsa-miR-155* | 40.00 | 40.00 | 40.00 | 40.00 | 32.70 | 26.71 | 35.32 | 40.00 | 40.00 | 26.53 | 40.00 | 40.00 |
| hsa-miR-15a | 37.14 | 29.49 | 32.80 | 30.01 | 40.00 | 31.46 | 27.19 | 28.59 | 35.31 | 34.56 | 38.43 | 32.48 |
| hsa-miR-15a* | 33.91 | 35.18 | 37.86 | 40.00 | 32.10 | 40.00 | 29.81 | 32.43 | 40.00 | 30.95 | 33.41 | 30.96 |
| hsa-miR-15b | 23.31 | 21.48 | 24.44 | 23.85 | 23.11 | 23.94 | 20.56 | 22.50 | 25.37 | 23.72 | 22.18 | 23.94 |
| hsa-miR-15b* | 40.00 | 40.00 | 40.00 | 40.00 | 40.00 | 38.06 | 28.05 | 40.00 | 40.00 | 34.20 | 40.00 | 40.00 |
| hsa-miR-16 | 25.53 | 23.19 | 26.74 | 24.97 | 24.73 | 25.94 | 20.55 | 24.06 | 25.67 | 24.47 | 22.18 | 25.01 |
| hsa-miR-16-1* | 40.00 | 32.78 | 40.00 | 33.42 | 35.43 | 40.00 | 28.58 | 40.00 | 40.00 | 36.56 | 33.41 | 39.57 |
| hsa-miR-16-2* | 40.00 | 40.00 | 40.00 | 40.00 | 40.00 | 29.14 | 38.98 | 40.00 | 40.00 | 29.94 | 40.00 | 40.00 |
| hsa-miR-17 | 22.82 | 20.10 | 24.18 | 20.89 | 21.48 | 21.35 | 17.34 | 21.38 | 23.56 | 20.23 | 20.78 | 19.21 |
| hsa-miR-17* | 22.13 | 40.00 | 40.00 | 40.00 | 40.00 | 31.99 | 28.83 | 40.00 | 40.00 | 33.85 | 40.00 | 35.52 |
| hsa-miR-181a | 21.51 | 22.01 | 23.40 | 24.90 | 21.78 | 23.60 | 22.13 | 21.48 | 25.45 | 22.13 | 23.01 | 20.59 |
| hsa-miR-181a* | 36.58 | 35.40 | 40.00 | 40.00 | 38.82 | 40.00 | 27.85 | 40.00 | 32.41 | 36.49 | 40.00 | 40.00 |
| hsa-miR-181a-2* | 27.68 | 27.49 | 28.63 | 29.77 | 25.72 | 27.98 | 24.42 | 29.21 | 28.80 | 26.22 | 27.07 | 27.08 |
| hsa-miR-181c | 27.62 | 26.98 | 29.09 | 28.70 | 27.16 | 28.70 | 27.38 | 26.58 | 30.74 | 27.33 | 28.33 | 25.64 |
| hsa-miR-181c* | 40.00 | 32.03 | 40.00 | 34.61 | 32.97 | 33.57 | 31.31 | 40.00 | 40.00 | 32.02 | 38.92 | 40.00 |
| hsa-miR-182 | 40.00 | 38.05 | 40.00 | 34.74 | 40.00 | 40.00 | 40.00 | 36.98 | 40.00 | 40.00 | 40.00 | 40.00 |
| hsa-miR-182* | 30.52 | 35.97 | 40.00 | 40.00 | 40.00 | 37.88 | 40.00 | 40.00 | 40.00 | 34.93 | 40.00 | 40.00 |
| hsa-miR-183 | 40.00 | 40.00 | 40.00 | 36.95 | 37.17 | 37.88 | 36.14 | 38.69 | 40.00 | 38.85 | 38.34 | 40.00 |
| hsa-miR-183* | 40.00 | 32.89 | 40.00 | 34.45 | 32.83 | 33.34 | 36.51 | 40.00 | 40.00 | 37.66 | 40.00 | 40.00 |
| hsa-miR-184 | 30.79 | 36.16 | 32.65 | 29.74 | 26.32 | 31.48 | 30.74 | 33.54 | 34.65 | 29.77 | 37.81 | 35.94 |
| hsa-miR-185 | 29.43 | 26.31 | 32.53 | 27.41 | 27.23 | 30.88 | 25.74 | 26.64 | 26.84 | 26.93 | 27.99 | 28.23 |
| hsa-miR-185* | 40.00 | 40.00 | 40.00 | 40.00 | 40.00 | 40.00 | 40.00 | 40.00 | 40.00 | 40.00 | 40.00 | 40.00 |
| hsa-miR-186 | 19.69 | 18.54 | 19.01 | 19.14 | 20.05 | 19.29 | 18.57 | 19.16 | 18.76 | 18.50 | 18.76 | 19.04 |
| hsa-miR-186* | 40.00 | 40.00 | 40.00 | 40.00 | 40.00 | 31.39 | 38.69 | 40.00 | 40.00 | 32.66 | 40.00 | 40.00 |
| hsa-miR-187 | 40.00 | 40.00 | 40.00 | 40.00 | 38.73 | 40.00 | 37.28 | 40.00 | 40.00 | 40.00 | 40.00 | 40.00 |
| hsa-miR-188-3p | 40.00 | 39.86 | 40.00 | 40.00 | 40.00 | 40.00 | 40.00 | 40.00 | 40.00 | 40.00 | 40.00 | 40.00 |
| hsa-miR-188-5p | 25.61 | 25.34 | 25.83 | 25.58 | 26.06 | 24.60 | 24.46 | 25.20 | 26.37 | 25.18 | 26.13 | 26.76 |
| hsa-miR-18a | 40.00 | 27.87 | 40.00 | 29.78 | 37.97 | 35.36 | 23.27 | 30.06 | 34.89 | 29.64 | 30.62 | 28.85 |
| hsa-miR-18a* | 29.87 | 10.84 | 33.55 | 27.95 | 29.16 | 26.87 | 25.60 | 29.46 | 32.19 | 29.50 | 27.83 | 27.67 |
| hsa-miR-18b | 40.00 | 34.45 | 40.00 | 40.00 | 40.00 | 40.00 | 28.63 | 40.00 | 40.00 | 40.00 | 40.00 | 40.00 |
| hsa-miR-18b* | 40.00 | 40.00 | 40.00 | 40.00 | 40.00 | 40.00 | 40.00 | 40.00 | 40.00 | 33.88 | 40.00 | 40.00 |
| hsa-miR-190 | 33.96 | 34.74 | 36.10 | 40.00 | 40.00 | 40.00 | 37.18 | 35.55 | 40.00 | 32.33 | 40.00 | 40.00 |
| hsa-miR-190b | 34.99 | 28.34 | 35.75 | 29.85 | 27.66 | 28.02 | 27.98 | 32.86 | 40.00 | 26.21 | 34.51 | 31.17 |
| hsa-miR-191 | 24.26 | 20.98 | 25.70 | 22.59 | 21.87 | 22.75 | 19.58 | 22.59 | 23.19 | 22.59 | 23.71 | 21.29 |
| hsa-miR-192 | 29.61 | 26.89 | 31.17 | 29.45 | 27.67 | 29.30 | 25.91 | 27.15 | 28.54 | 28.14 | 29.71 | 28.86 |
| hsa-miR-192* | 24.08 | 30.20 | 40.00 | 40.00 | 29.32 | 30.14 | 29.37 | 40.00 | 40.00 | 30.46 | 32.22 | 33.00 |
| hsa-miR-193a-3p | 40.00 | 34.12 | 40.00 | 40.00 | 36.25 | 35.76 | 33.93 | 33.48 | 36.91 | 35.99 | 39.35 | 40.00 |
| hsa-miR-193a-5p | 22.55 | 22.63 | 22.84 | 23.95 | 23.99 | 24.04 | 23.95 | 21.75 | 22.66 | 25.11 | 23.89 | 23.99 |
| hsa-miR-193b | 21.55 | 21.60 | 22.98 | 20.67 | 20.28 | 21.63 | 21.59 | 20.60 | 22.07 | 19.07 | 22.64 | 20.38 |
| hsa-miR-193b* | 28.34 | 31.13 | 30.99 | 29.18 | 27.46 | 29.01 | 31.34 | 29.07 | 31.62 | 25.99 | 35.22 | 30.48 |
| hsa-miR-194 | 25.91 | 25.24 | 29.55 | 27.37 | 25.44 | 26.75 | 24.98 | 25.48 | 26.90 | 26.57 | 26.40 | 27.85 |
| hsa-miR-194* | 40.00 | 40.00 | 40.00 | 40.00 | 37.04 | 37.23 | 40.00 | 40.00 | 40.00 | 39.17 | 40.00 | 40.00 |
| hsa-miR-195 | 24.88 | 24.23 | 26.52 | 26.97 | 25.92 | 26.64 | 22.32 | 25.08 | 27.96 | 25.92 | 25.47 | 27.58 |
| hsa-miR-195* | 40.00 | 40.00 | 40.00 | 40.00 | 40.00 | 36.93 | 40.00 | 40.00 | 40.00 | 40.00 | 40.00 | 40.00 |
| hsa-miR-196b | 40.00 | 30.15 | 33.79 | 31.82 | 40.00 | 33.29 | 27.33 | 34.36 | 31.53 | 32.63 | 40.00 | 32.95 |
| hsa-miR-197 | 21.75 | 21.35 | 22.35 | 20.46 | 20.43 | 22.24 | 22.80 | 21.19 | 23.97 | 20.92 | 24.97 | 19.53 |
| hsa-miR-198 | 32.04 | 32.31 | 35.13 | 39.13 | 40.00 | 35.68 | 36.38 | 38.25 | 32.22 | 40.00 | 35.90 | 40.00 |
| hsa-miR-199a-3p | 24.14 | 24.33 | 26.48 | 26.05 | 24.60 | 25.47 | 21.40 | 23.45 | 24.29 | 24.59 | 24.98 | 26.01 |
| hsa-miR-199a-5p | 28.20 | 31.55 | 36.14 | 32.54 | 30.43 | 30.68 | 27.31 | 29.36 | 30.46 | 31.51 | 30.43 | 35.27 |
| hsa-miR-199b-5p | 31.12 | 30.89 | 39.41 | 33.88 | 33.31 | 33.02 | 28.03 | 30.58 | 31.20 | 40.00 | 32.97 | 40.00 |
| hsa-miR-19a | 28.77 | 25.84 | 31.16 | 30.34 | 34.76 | 31.78 | 24.73 | 27.47 | 29.76 | 27.80 | 28.82 | 29.62 |
| hsa-miR-19a* | 34.51 | 40.00 | 40.00 | 40.00 | 40.00 | 37.62 | 40.00 | 40.00 | 40.00 | 33.21 | 40.00 | 40.00 |
| hsa-miR-19b | 24.73 | 21.97 | 26.39 | 25.27 | 25.84 | 25.59 | 20.03 | 23.34 | 25.65 | 22.86 | 23.57 | 23.71 |
| hsa-miR-19b-1* | 40.00 | 29.88 | 33.18 | 30.06 | 30.40 | 29.85 | 27.13 | 31.74 | 31.75 | 28.96 | 30.39 | 29.94 |
| hsa-miR-19b-2* | 40.00 | 22.31 | 40.00 | 40.00 | 40.00 | 34.83 | 35.35 | 40.00 | 40.00 | 39.31 | 40.00 | 40.00 |
| hsa-miR-200a | 35.52 | 35.46 | 40.00 | 35.20 | 35.22 | 40.00 | 38.35 | 34.50 | 35.20 | 37.41 | 38.67 | 40.00 |
| hsa-miR-200a* | 36.69 | 37.13 | 40.00 | 36.89 | 35.71 | 27.69 | 32.90 | 40.00 | 40.00 | 32.08 | 40.00 | 37.24 |
| hsa-miR-200b | 40.00 | 33.78 | 40.00 | 40.00 | 40.00 | 40.00 | 30.92 | 34.50 | 34.51 | 40.00 | 40.00 | 35.68 |
| hsa-miR-200b* | 36.85 | 40.00 | 40.00 | 40.00 | 40.00 | 32.37 | 40.00 | 40.00 | 40.00 | 40.00 | 40.00 | 40.00 |
| hsa-miR-200c | 31.65 | 27.67 | 34.06 | 31.19 | 27.61 | 29.41 | 27.36 | 27.74 | 31.97 | 29.93 | 34.22 | 28.33 |
| hsa-miR-200c* | 40.00 | 40.00 | 40.00 | 40.00 | 37.26 | 30.27 | 37.64 | 40.00 | 40.00 | 37.66 | 40.00 | 40.00 |
| hsa-miR-202 | 31.59 | 29.68 | 31.41 | 29.85 | 30.27 | 33.76 | 29.48 | 29.27 | 31.41 | 29.13 | 32.59 | 32.45 |
| hsa-miR-202* | 38.29 | 40.00 | 40.00 | 40.00 | 40.00 | 38.20 | 40.00 | 40.00 | 40.00 | 32.91 | 40.00 | 40.00 |
| hsa-miR-203 | 40.00 | 29.49 | 33.24 | 29.40 | 32.70 | 40.00 | 29.28 | 30.01 | 27.83 | 30.18 | 39.93 | 40.00 |
| hsa-miR-204 | 24.41 | 26.32 | 24.73 | 22.35 | 25.01 | 27.02 | 24.71 | 26.69 | 29.95 | 23.41 | 28.85 | 25.24 |
| hsa-miR-205 | 40.00 | 38.19 | 40.00 | 40.00 | 40.00 | 40.00 | 39.27 | 40.00 | 40.00 | 40.00 | 40.00 | 33.09 |
| hsa-miR-206 | 40.00 | 29.08 | 29.90 | 30.75 | 29.23 | 28.77 | 27.72 | 29.75 | 30.30 | 28.44 | 31.01 | 29.71 |
| hsa-miR-208 | 40.00 | 32.68 | 40.00 | 40.00 | 37.21 | 40.00 | 39.01 | 40.00 | 40.00 | 34.67 | 40.00 | 40.00 |
| hsa-miR-208b | 39.25 | 31.46 | 40.00 | 40.00 | 35.04 | 33.91 | 32.21 | 31.58 | 34.73 | 34.20 | 40.00 | 40.00 |
| hsa-miR-20a | 25.34 | 21.64 | 25.98 | 23.40 | 23.75 | 23.72 | 18.75 | 23.26 | 25.75 | 22.17 | 22.41 | 21.23 |
| hsa-miR-20a* | 40.00 | 29.62 | 32.50 | 35.39 | 33.49 | 33.60 | 28.11 | 32.54 | 33.67 | 30.01 | 31.08 | 33.40 |
| hsa-miR-20b | 25.87 | 22.63 | 27.15 | 24.59 | 25.00 | 24.72 | 20.55 | 24.25 | 26.47 | 23.56 | 23.86 | 22.79 |
| hsa-miR-20b* | 40.00 | 40.00 | 40.00 | 40.00 | 40.00 | 35.90 | 40.00 | 40.00 | 40.00 | 40.00 | 40.00 | 40.00 |
| hsa-miR-21 | 25.56 | 24.68 | 29.23 | 28.51 | 26.88 | 28.44 | 23.09 | 23.71 | 24.36 | 25.81 | 28.02 | 28.89 |
| hsa-miR-21* | 25.06 | 28.32 | 25.51 | 29.20 | 29.69 | 27.62 | 26.58 | 27.77 | 25.66 | 26.77 | 29.38 | 34.35 |
| hsa-miR-210 | 30.95 | 25.03 | 32.33 | 24.97 | 26.75 | 29.85 | 25.35 | 29.05 | 26.43 | 24.85 | 27.62 | 25.06 |
| hsa-miR-211 | 31.77 | 31.33 | 33.18 | 34.41 | 35.17 | 31.49 | 31.74 | 26.26 | 32.98 | 30.79 | 31.20 | 33.86 |
| hsa-miR-212 | 22.30 | 21.25 | 24.91 | 24.40 | 22.42 | 21.26 | 20.59 | 19.75 | 23.47 | 22.43 | 24.32 | 23.36 |
| hsa-miR-214 | 22.49 | 23.92 | 24.18 | 24.80 | 21.86 | 22.79 | 22.87 | 20.96 | 23.34 | 23.64 | 21.81 | 26.56 |
| hsa-miR-214* | 27.92 | 29.17 | 30.76 | 31.19 | 27.19 | 27.51 | 25.11 | 28.44 | 27.38 | 28.53 | 28.05 | 30.46 |
| hsa-miR-215 | 35.02 | 31.89 | 40.00 | 30.81 | 37.22 | 34.25 | 30.29 | 32.59 | 38.15 | 29.77 | 38.37 | 32.91 |
| hsa-miR-216a | 40.00 | 40.00 | 40.00 | 39.93 | 37.84 | 36.92 | 40.00 | 40.00 | 40.00 | 40.00 | 40.00 | 35.36 |
| hsa-miR-216b | 40.00 | 39.88 | 40.00 | 33.31 | 35.30 | 29.36 | 28.60 | 40.00 | 40.00 | 33.62 | 40.00 | 34.06 |
| hsa-miR-217 | 40.00 | 40.00 | 40.00 | 40.00 | 32.91 | 40.00 | 31.87 | 40.00 | 40.00 | 40.00 | 40.00 | 40.00 |
| hsa-miR-218 | 24.23 | 21.53 | 25.94 | 22.20 | 24.99 | 24.22 | 19.41 | 23.09 | 27.66 | 20.33 | 23.93 | 21.81 |
| hsa-miR-218-2* | 27.27 | 27.11 | 29.25 | 25.78 | 27.85 | 28.29 | 24.61 | 28.99 | 33.52 | 25.58 | 26.72 | 25.49 |
| hsa-miR-219-1-3p | 40.00 | 40.00 | 40.00 | 39.09 | 40.00 | 34.41 | 40.00 | 40.00 | 40.00 | 40.00 | 40.00 | 34.93 |
| hsa-miR-219-2-3p | 40.00 | 40.00 | 40.00 | 40.00 | 40.00 | 40.00 | 40.00 | 40.00 | 40.00 | 40.00 | 40.00 | 40.00 |
| hsa-miR-219-5p | 40.00 | 33.06 | 37.30 | 36.73 | 39.62 | 40.00 | 32.93 | 32.86 | 35.16 | 35.28 | 33.43 | 38.36 |
| hsa-miR-22 | 34.10 | 31.57 | 40.00 | 37.24 | 32.69 | 40.00 | 27.41 | 31.25 | 32.78 | 32.75 | 38.55 | 40.00 |
| hsa-miR-22* | 32.79 | 30.54 | 32.99 | 32.36 | 31.20 | 35.61 | 28.00 | 31.20 | 30.94 | 29.27 | 39.35 | 34.58 |
| hsa-miR-220 | 40.00 | 40.00 | 40.00 | 40.00 | 40.00 | 40.00 | 40.00 | 40.00 | 40.00 | 40.00 | 40.00 | 40.00 |
| hsa-miR-220b | 40.00 | 40.00 | 40.00 | 37.36 | 36.63 | 40.00 | 31.76 | 40.00 | 34.31 | 35.28 | 40.00 | 40.00 |
| hsa-miR-220c | 40.00 | 40.00 | 40.00 | 40.00 | 40.00 | 40.00 | 40.00 | 40.00 | 40.00 | 40.00 | 40.00 | 40.00 |
| hsa-miR-221 | 26.20 | 24.73 | 28.14 | 26.57 | 25.37 | 27.35 | 22.88 | 25.51 | 25.96 | 25.38 | 25.73 | 24.51 |
| hsa-miR-221* | 40.00 | 40.00 | 40.00 | 40.00 | 40.00 | 35.22 | 31.18 | 40.00 | 40.00 | 29.62 | 40.00 | 40.00 |
| hsa-miR-222 | 21.25 | 20.96 | 23.19 | 23.46 | 20.67 | 21.58 | 18.41 | 21.12 | 22.81 | 23.29 | 21.47 | 21.07 |
| hsa-miR-222* | 38.92 | 40.00 | 40.00 | 40.00 | 40.00 | 36.70 | 31.87 | 38.53 | 40.00 | 38.09 | 40.00 | 40.00 |
| hsa-miR-223 | 24.65 | 24.60 | 27.29 | 28.32 | 26.34 | 26.12 | 19.04 | 22.96 | 24.28 | 27.67 | 24.48 | 27.69 |
| hsa-miR-223* | 40.00 | 32.99 | 33.49 | 40.00 | 40.00 | 32.51 | 27.50 | 31.91 | 31.02 | 33.61 | 40.00 | 40.00 |
| hsa-miR-224 | 27.95 | 27.88 | 29.89 | 31.33 | 27.39 | 31.68 | 24.93 | 29.66 | 28.49 | 27.87 | 29.22 | 29.09 |
| hsa-miR-23a | 40.00 | 36.66 | 40.00 | 40.00 | 35.03 | 40.00 | 33.78 | 34.34 | 35.63 | 34.34 | 40.00 | 35.99 |
| hsa-miR-23a* | 36.69 | 32.65 | 31.27 | 33.96 | 34.81 | 40.00 | 31.68 | 36.17 | 40.00 | 29.78 | 35.74 | 40.00 |
| hsa-miR-23b | 29.26 | 35.37 | 30.21 | 26.99 | 26.75 | 34.06 | 27.78 | 30.54 | 35.31 | 27.95 | 35.48 | 27.28 |
| hsa-miR-23b* | 40.00 | 40.00 | 40.00 | 35.35 | 33.76 | 38.91 | 36.79 | 40.00 | 40.00 | 37.64 | 40.00 | 40.00 |
| hsa-miR-24 | 20.17 | 19.88 | 21.31 | 18.77 | 18.93 | 21.33 | 18.71 | 19.82 | 20.58 | 18.32 | 22.09 | 18.41 |
| hsa-miR-24-1* | 40.00 | 40.00 | 40.00 | 40.00 | 40.00 | 36.30 | 40.00 | 40.00 | 40.00 | 35.26 | 40.00 | 40.00 |
| hsa-miR-25 | 27.42 | 22.66 | 27.38 | 24.45 | 24.16 | 24.72 | 20.83 | 25.69 | 26.67 | 24.84 | 24.07 | 23.96 |
| hsa-miR-25* | 38.54 | 30.12 | 40.00 | 38.42 | 35.44 | 31.21 | 27.34 | 40.00 | 40.00 | 32.64 | 37.74 | 32.78 |
| hsa-miR-26a | 22.02 | 21.97 | 22.72 | 21.67 | 21.19 | 23.40 | 19.97 | 21.83 | 23.72 | 21.74 | 24.33 | 20.71 |
| hsa-miR-26a-1* | 38.73 | 40.00 | 40.00 | 40.00 | 37.01 | 40.00 | 30.88 | 36.52 | 40.00 | 38.01 | 40.00 | 40.00 |
| hsa-miR-26a-2* | 40.00 | 40.00 | 40.00 | 40.00 | 40.00 | 34.13 | 40.00 | 40.00 | 40.00 | 33.43 | 40.00 | 40.00 |
| hsa-miR-26b | 27.03 | 26.79 | 28.50 | 28.19 | 26.97 | 28.47 | 22.64 | 26.29 | 28.02 | 26.24 | 28.98 | 25.83 |
| hsa-miR-26b* | 30.50 | 30.46 | 31.44 | 33.01 | 30.77 | 31.44 | 28.58 | 32.54 | 33.26 | 29.47 | 32.68 | 29.38 |
| hsa-miR-27a | 26.02 | 25.13 | 27.06 | 25.48 | 24.81 | 26.98 | 24.01 | 24.36 | 25.19 | 24.92 | 27.86 | 26.43 |
| hsa-miR-27a* | 31.34 | 29.43 | 34.07 | 33.62 | 31.13 | 29.37 | 30.09 | 29.15 | 32.18 | 31.07 | 30.01 | 37.22 |
| hsa-miR-27b | 24.99 | 25.30 | 26.39 | 23.67 | 23.33 | 27.99 | 22.75 | 25.45 | 26.16 | 23.55 | 27.48 | 24.73 |
| hsa-miR-27b* | 32.54 | 37.82 | 40.00 | 31.95 | 33.52 | 34.08 | 27.23 | 38.07 | 40.00 | 30.89 | 37.72 | 33.52 |
| hsa-miR-28-3p | 25.50 | 26.18 | 26.83 | 26.45 | 26.18 | 25.78 | 23.69 | 24.54 | 24.90 | 25.39 | 27.22 | 26.24 |
| hsa-miR-28-5p | 27.87 | 27.00 | 28.91 | 28.78 | 27.77 | 28.17 | 24.20 | 25.64 | 26.25 | 27.53 | 29.12 | 27.71 |
| hsa-miR-296-3p | 37.86 | 33.42 | 35.73 | 33.79 | 39.97 | 37.36 | 30.40 | 35.35 | 40.00 | 33.91 | 32.47 | 31.64 |
| hsa-miR-296-5p | 26.15 | 25.43 | 26.86 | 25.27 | 24.79 | 26.98 | 24.79 | 26.67 | 28.45 | 26.55 | 28.60 | 23.18 |
| hsa-miR-298 | 40.00 | 40.00 | 40.00 | 40.00 | 40.00 | 40.00 | 39.76 | 40.00 | 40.00 | 40.00 | 40.00 | 40.00 |
| hsa-miR-299-3p | 40.00 | 40.00 | 40.00 | 40.00 | 40.00 | 40.00 | 39.10 | 40.00 | 40.00 | 38.40 | 40.00 | 40.00 |
| hsa-miR-299-5p | 40.00 | 33.14 | 20.08 | 37.07 | 40.00 | 33.14 | 35.03 | 40.00 | 36.92 | 40.00 | 40.00 | 33.67 |
| hsa-miR-29a | 23.67 | 22.63 | 25.35 | 24.35 | 21.92 | 24.30 | 22.12 | 23.23 | 24.65 | 23.38 | 25.96 | 25.85 |
| hsa-miR-29a* | 35.06 | 33.61 | 40.00 | 40.00 | 36.40 | 31.15 | 30.78 | 40.00 | 35.83 | 29.57 | 40.00 | 40.00 |
| hsa-miR-29b | 30.22 | 28.92 | 34.63 | 29.94 | 32.96 | 30.50 | 27.17 | 28.21 | 28.67 | 27.95 | 29.27 | 35.10 |
| hsa-miR-29b-1* | 40.00 | 40.00 | 40.00 | 40.00 | 32.55 | 31.39 | 31.49 | 40.00 | 40.00 | 32.00 | 40.00 | 40.00 |
| hsa-miR-29b-2* | 33.32 | 31.07 | 33.14 | 30.53 | 27.89 | 30.03 | 29.55 | 32.52 | 31.94 | 27.95 | 31.59 | 33.53 |
| hsa-miR-29c | 22.38 | 20.72 | 20.73 | 21.18 | 22.10 | 21.56 | 20.57 | 20.16 | 20.07 | 20.78 | 20.02 | 20.08 |
| hsa-miR-29c* | 29.09 | 28.39 | 33.34 | 28.98 | 27.44 | 28.86 | 26.94 | 29.21 | 28.61 | 28.04 | 40.00 | 29.84 |
| hsa-miR-301a | 30.07 | 25.40 | 25.20 | 30.47 | 30.33 | 29.19 | 24.92 | 30.66 | 33.05 | 27.98 | 29.92 | 31.30 |
| hsa-miR-301b | 32.26 | 29.58 | 40.00 | 34.11 | 40.00 | 40.00 | 27.20 | 31.39 | 40.00 | 30.32 | 32.64 | 32.47 |
| hsa-miR-302a | 32.65 | 31.69 | 35.14 | 37.72 | 26.99 | 38.57 | 34.25 | 34.38 | 32.05 | 37.39 | 36.04 | 40.00 |
| hsa-miR-302a* | 40.00 | 34.50 | 40.00 | 32.53 | 40.00 | 36.80 | 35.14 | 40.00 | 40.00 | 32.25 | 40.00 | 36.14 |
| hsa-miR-302b | 40.00 | 32.30 | 40.00 | 40.00 | 40.00 | 40.00 | 34.70 | 40.00 | 40.00 | 40.00 | 40.00 | 40.00 |
| hsa-miR-302b* | 40.00 | 40.00 | 40.00 | 40.00 | 40.00 | 40.00 | 40.00 | 40.00 | 40.00 | 40.00 | 40.00 | 40.00 |
| hsa-miR-302c | 25.46 | 23.18 | 24.87 | 24.50 | 24.65 | 24.82 | 23.69 | 23.38 | 23.25 | 24.36 | 23.64 | 24.86 |
| hsa-miR-302c* | 32.67 | 37.87 | 40.00 | 40.00 | 40.00 | 33.72 | 40.00 | 40.00 | 40.00 | 25.66 | 40.00 | 40.00 |
| hsa-miR-302d | 40.00 | 40.00 | 40.00 | 40.00 | 40.00 | 40.00 | 34.38 | 40.00 | 40.00 | 30.32 | 40.00 | 40.00 |
| hsa-miR-302d | 40.00 | 40.00 | 40.00 | 40.00 | 40.00 | 40.00 | 40.00 | 40.00 | 40.00 | 40.00 | 35.15 | 40.00 |
| hsa-miR-302d* | 40.00 | 40.00 | 40.00 | 40.00 | 40.00 | 40.00 | 40.00 | 40.00 | 40.00 | 40.00 | 40.00 | 40.00 |
| hsa-miR-30a | 22.93 | 21.82 | 24.13 | 22.81 | 22.52 | 23.30 | 20.89 | 23.48 | 24.31 | 22.52 | 24.66 | 22.55 |
| hsa-miR-30a* | 24.74 | 25.66 | 26.42 | 25.71 | 25.54 | 26.61 | 24.04 | 26.07 | 28.29 | 26.37 | 28.84 | 27.48 |
| hsa-miR-30b | 24.30 | 22.80 | 24.59 | 23.19 | 23.08 | 23.84 | 20.54 | 23.09 | 24.51 | 22.09 | 24.39 | 22.45 |
| hsa-miR-30b* | 40.00 | 40.00 | 40.00 | 40.00 | 40.00 | 33.63 | 40.00 | 40.00 | 40.00 | 40.00 | 40.00 | 40.00 |
| hsa-miR-30c | 23.66 | 22.02 | 24.24 | 22.48 | 22.56 | 22.78 | 19.91 | 22.97 | 23.97 | 21.53 | 23.84 | 21.71 |
| hsa-miR-30c-1* | 38.53 | 40.00 | 40.00 | 40.00 | 40.00 | 40.00 | 32.44 | 40.00 | 40.00 | 37.65 | 40.00 | 37.20 |
| hsa-miR-30c-2* | 40.00 | 40.00 | 40.00 | 40.00 | 40.00 | 38.99 | 40.00 | 40.00 | 40.00 | 34.83 | 40.00 | 40.00 |
| hsa-miR-30d | 25.40 | 24.20 | 26.48 | 25.05 | 24.37 | 24.38 | 22.72 | 25.72 | 26.57 | 40.00 | 26.97 | 24.20 |
| hsa-miR-30d | 25.56 | 24.36 | 26.87 | 25.16 | 24.13 | 24.22 | 23.12 | 25.80 | 27.10 | 40.00 | 26.70 | 24.88 |
| hsa-miR-30d* | 33.93 | 34.03 | 33.23 | 40.00 | 40.00 | 31.03 | 28.42 | 32.79 | 34.70 | 31.42 | 40.00 | 31.88 |
| hsa-miR-30e | 25.82 | 24.58 | 27.24 | 25.41 | 25.26 | 24.99 | 23.11 | 26.12 | 26.73 | 24.56 | 26.82 | 25.40 |
| hsa-miR-30e* | 24.89 | 24.60 | 26.42 | 25.95 | 25.64 | 25.71 | 23.63 | 26.28 | 26.80 | 25.25 | 27.90 | 26.87 |
| hsa-miR-31 | 32.88 | 30.21 | 33.49 | 32.02 | 31.49 | 33.32 | 24.97 | 26.66 | 28.07 | 35.90 | 28.15 | 40.00 |
| hsa-miR-32 | 34.73 | 40.00 | 40.00 | 40.00 | 40.00 | 40.00 | 33.06 | 33.27 | 40.00 | 40.00 | 40.00 | 40.00 |
| hsa-miR-32* | 40.00 | 40.00 | 40.00 | 40.00 | 40.00 | 35.51 | 39.88 | 40.00 | 40.00 | 40.00 | 40.00 | 40.00 |
| hsa-miR-320 | 20.35 | 20.22 | 20.71 | 19.56 | 19.96 | 20.05 | 20.11 | 19.48 | 22.22 | 20.29 | 19.79 | 18.65 |
| hsa-miR-323-3p | 28.22 | 25.63 | 30.08 | 25.12 | 31.15 | 25.50 | 25.86 | 27.51 | 30.61 | 29.84 | 29.68 | 24.95 |
| hsa-miR-324-3p | 18.07 | 17.98 | 17.99 | 17.62 | 18.32 | 18.07 | 17.91 | 17.75 | 17.92 | 17.46 | 17.87 | 18.02 |
| hsa-miR-324-5p | 24.23 | 22.26 | 26.23 | 22.52 | 22.93 | 22.76 | 22.33 | 22.91 | 25.59 | 23.77 | 25.62 | 21.89 |
| hsa-miR-325 | 40.00 | 32.97 | 40.00 | 40.00 | 40.00 | 40.00 | 40.00 | 40.00 | 36.92 | 40.00 | 40.00 | 40.00 |
| hsa-miR-326 | 36.39 | 40.00 | 34.46 | 30.40 | 31.39 | 32.03 | 31.11 | 30.62 | 32.10 | 31.62 | 37.07 | 32.86 |
| hsa-miR-328 | 18.74 | 19.42 | 19.51 | 18.77 | 18.57 | 19.58 | 20.63 | 18.06 | 22.22 | 18.34 | 22.22 | 17.95 |
| hsa-miR-329 | 34.73 | 31.27 | 40.00 | 30.42 | 40.00 | 34.04 | 30.51 | 31.39 | 34.96 | 39.68 | 33.64 | 29.97 |
| hsa-miR-330-3p | 32.03 | 29.09 | 37.09 | 28.89 | 29.15 | 30.98 | 32.06 | 30.73 | 33.44 | 29.43 | 35.19 | 28.23 |
| hsa-miR-330-5p | 37.92 | 30.65 | 40.00 | 40.00 | 30.59 | 32.12 | 38.89 | 33.94 | 38.13 | 40.00 | 40.00 | 31.75 |
| hsa-miR-331-3p | 20.94 | 19.37 | 22.53 | 19.94 | 18.58 | 19.59 | 19.98 | 19.67 | 21.70 | 19.89 | 22.35 | 17.93 |
| hsa-miR-331-5p | 29.13 | 27.49 | 30.41 | 28.25 | 28.07 | 28.10 | 27.70 | 28.12 | 29.96 | 26.98 | 31.88 | 27.69 |
| hsa-miR-335 | 27.52 | 27.28 | 30.43 | 30.44 | 31.35 | 32.26 | 28.88 | 28.95 | 32.63 | 26.96 | 31.26 | 30.36 |
| hsa-miR-335* | 30.58 | 28.63 | 31.53 | 31.45 | 29.34 | 28.37 | 27.31 | 31.96 | 33.33 | 29.71 | 30.66 | 32.36 |
| hsa-miR-337-3p | 40.00 | 40.00 | 40.00 | 36.96 | 40.00 | 33.14 | 40.00 | 40.00 | 40.00 | 35.63 | 40.00 | 40.00 |
| hsa-miR-337-5p | 32.19 | 29.83 | 32.85 | 30.12 | 40.00 | 29.20 | 30.10 | 30.14 | 31.02 | 34.08 | 34.28 | 29.80 |
| hsa-miR-338-3p | 34.81 | 36.10 | 37.18 | 40.00 | 40.00 | 40.00 | 32.87 | 37.95 | 40.00 | 35.59 | 40.00 | 40.00 |
| hsa-miR-339-3p | 33.44 | 26.45 | 32.01 | 26.79 | 27.77 | 26.56 | 26.88 | 26.96 | 29.50 | 27.12 | 29.81 | 26.15 |
| hsa-miR-339-5p | 25.82 | 25.62 | 28.38 | 25.33 | 25.03 | 27.23 | 23.80 | 24.02 | 25.35 | 24.79 | 27.49 | 24.36 |
| hsa-miR-33a* | 36.36 | 32.89 | 36.35 | 34.64 | 33.93 | 40.00 | 29.77 | 40.00 | 34.09 | 33.32 | 40.00 | 40.00 |
| hsa-miR-33b | 32.02 | 26.63 | 33.15 | 27.16 | 27.84 | 30.84 | 26.35 | 32.33 | 35.71 | 28.22 | 31.68 | 28.89 |
| hsa-miR-340 | 29.43 | 26.09 | 30.21 | 27.01 | 28.10 | 29.74 | 23.99 | 28.11 | 29.00 | 25.99 | 29.90 | 29.32 |
| hsa-miR-340* | 30.85 | 27.39 | 31.79 | 29.41 | 27.95 | 28.78 | 25.30 | 30.96 | 29.84 | 28.10 | 32.16 | 30.28 |
| hsa-miR-342-3p | 23.73 | 21.46 | 25.39 | 21.69 | 22.97 | 22.43 | 19.02 | 22.87 | 23.15 | 22.19 | 24.45 | 21.81 |
| hsa-miR-342-5p | 29.79 | 28.18 | 30.59 | 26.51 | 29.37 | 28.60 | 26.38 | 27.37 | 30.66 | 29.58 | 30.33 | 26.22 |
| hsa-miR-345 | 24.93 | 16.31 | 25.74 | 23.14 | 24.29 | 23.58 | 20.48 | 23.71 | 25.08 | 25.05 | 24.13 | 23.26 |
| hsa-miR-346 | 28.68 | 24.95 | 26.91 | 28.00 | 28.72 | 27.13 | 25.69 | 26.22 | 26.62 | 25.85 | 27.39 | 26.33 |
| hsa-miR-34a | 23.47 | 21.93 | 25.48 | 22.76 | 23.69 | 25.53 | 22.05 | 22.24 | 22.45 | 24.04 | 25.12 | 24.21 |
| hsa-miR-34a* | 28.68 | 28.94 | 31.33 | 30.39 | 30.44 | 30.77 | 26.71 | 28.22 | 29.62 | 31.46 | 31.47 | 40.00 |
| hsa-miR-34b* | 40.00 | 34.59 | 40.00 | 40.00 | 40.00 | 40.00 | 31.73 | 40.00 | 40.00 | 35.06 | 40.00 | 40.00 |
| hsa-miR-34c-5p | 31.40 | 27.77 | 38.91 | 31.48 | 31.00 | 36.98 | 25.98 | 31.04 | 33.31 | 32.98 | 32.89 | 32.28 |
| hsa-miR-361-3p | 37.19 | 40.00 | 40.00 | 40.00 | 40.00 | 40.00 | 40.00 | 40.00 | 40.00 | 26.51 | 40.00 | 36.96 |
| hsa-miR-361-3p | 37.44 | 40.00 | 40.00 | 40.00 | 40.00 | 40.00 | 40.00 | 40.00 | 38.73 | 26.97 | 40.00 | 40.00 |
| hsa-miR-361-5p | 29.00 | 25.32 | 29.28 | 26.53 | 25.99 | 26.69 | 24.67 | 25.98 | 28.08 | 25.22 | 29.06 | 25.08 |
| hsa-miR-362-3p | 31.10 | 31.72 | 40.00 | 40.00 | 40.00 | 40.00 | 31.85 | 32.27 | 40.00 | 31.22 | 40.00 | 31.02 |
| hsa-miR-362-5p | 27.59 | 26.89 | 29.69 | 27.29 | 27.11 | 30.33 | 27.02 | 27.96 | 28.82 | 25.85 | 29.74 | 26.14 |
| hsa-miR-363 | 31.34 | 32.79 | 32.46 | 33.05 | 31.69 | 31.61 | 26.98 | 32.16 | 32.11 | 31.02 | 31.05 | 33.53 |
| hsa-miR-363* | 35.36 | 35.06 | 40.00 | 36.52 | 31.71 | 32.89 | 35.61 | 40.00 | 40.00 | 28.23 | 40.00 | 40.00 |
| hsa-miR-365 | 30.71 | 27.13 | 30.75 | 29.89 | 27.51 | 29.86 | 25.34 | 27.44 | 28.62 | 27.07 | 30.43 | 28.72 |
| hsa-miR-367 | 22.06 | 20.47 | 21.01 | 21.97 | 22.16 | 21.31 | 20.80 | 19.96 | 20.46 | 21.34 | 21.35 | 20.36 |
| hsa-miR-367* | 40.00 | 40.00 | 40.00 | 40.00 | 40.00 | 40.00 | 40.00 | 40.00 | 40.00 | 40.00 | 40.00 | 40.00 |
| hsa-miR-369-3p | 40.00 | 30.34 | 40.00 | 31.56 | 40.00 | 40.00 | 40.00 | 40.00 | 40.00 | 40.00 | 40.00 | 33.51 |
| hsa-miR-369-5p | 34.70 | 26.09 | 40.00 | 33.87 | 40.00 | 35.91 | 34.11 | 32.61 | 40.00 | 40.00 | 40.00 | 40.00 |
| hsa-miR-370 | 20.98 | 21.29 | 23.03 | 19.80 | 27.52 | 20.25 | 22.19 | 21.63 | 25.63 | 26.07 | 23.95 | 20.35 |
| hsa-miR-371-3p | 40.00 | 40.00 | 40.00 | 40.00 | 40.00 | 40.00 | 40.00 | 40.00 | 40.00 | 40.00 | 40.00 | 40.00 |
| hsa-miR-372 | 37.82 | 32.20 | 35.94 | 37.34 | 37.04 | 35.16 | 34.49 | 34.16 | 35.23 | 36.19 | 36.85 | 37.23 |
| hsa-miR-373 | 40.00 | 32.95 | 31.96 | 40.00 | 32.93 | 32.02 | 31.06 | 33.87 | 38.29 | 34.13 | 40.00 | 40.00 |
| hsa-miR-373* | 36.73 | 39.00 | 40.00 | 40.00 | 40.00 | 37.34 | 32.29 | 40.00 | 40.00 | 40.00 | 36.28 | 40.00 |
| hsa-miR-374a | 27.40 | 25.64 | 30.06 | 31.19 | 29.23 | 30.98 | 25.13 | 26.11 | 28.32 | 26.53 | 30.01 | 29.13 |
| hsa-miR-374a* | 40.00 | 40.00 | 40.00 | 40.00 | 40.00 | 40.00 | 40.00 | 40.00 | 40.00 | 40.00 | 40.00 | 40.00 |
| hsa-miR-374b | 27.37 | 24.93 | 28.93 | 27.04 | 26.90 | 28.32 | 22.54 | 25.00 | 29.16 | 25.33 | 29.74 | 26.46 |
| hsa-miR-374b* | 40.00 | 38.42 | 40.00 | 40.00 | 35.62 | 28.60 | 31.60 | 40.00 | 40.00 | 28.60 | 33.63 | 40.00 |
| hsa-miR-375 | 24.85 | 19.05 | 27.40 | 24.02 | 23.03 | 19.79 | 21.72 | 17.34 | 30.64 | 17.01 | 24.40 | 15.32 |
| hsa-miR-376a | 25.70 | 24.77 | 26.93 | 26.45 | 27.11 | 26.18 | 24.09 | 25.04 | 25.85 | 25.94 | 25.54 | 25.81 |
| hsa-miR-376a* | 40.00 | 37.62 | 36.10 | 40.00 | 35.77 | 33.14 | 32.10 | 40.00 | 40.00 | 30.15 | 40.00 | 40.00 |
| hsa-miR-376b | 36.26 | 37.95 | 40.00 | 40.00 | 40.00 | 40.00 | 40.00 | 40.00 | 34.92 | 40.00 | 40.00 | 35.44 |
| hsa-miR-376c | 27.83 | 25.89 | 31.57 | 27.11 | 34.92 | 28.73 | 25.54 | 26.99 | 34.51 | 31.20 | 29.55 | 26.33 |
| hsa-miR-377 | 40.00 | 34.37 | 40.00 | 40.00 | 40.00 | 40.00 | 40.00 | 40.00 | 40.00 | 40.00 | 40.00 | 40.00 |
| hsa-miR-377* | 29.53 | 27.63 | 31.33 | 27.85 | 34.16 | 28.79 | 31.24 | 32.98 | 40.00 | 40.00 | 34.29 | 30.11 |
| hsa-miR-378 | 27.18 | 25.96 | 27.52 | 27.00 | 27.35 | 25.65 | 24.93 | 26.99 | 26.25 | 24.90 | 27.13 | 26.42 |
| hsa-miR-378* | 29.36 | 30.44 | 31.37 | 29.10 | 29.75 | 30.08 | 27.64 | 29.88 | 28.91 | 25.25 | 32.30 | 30.38 |
| hsa-miR-379 | 30.11 | 26.19 | 34.85 | 25.98 | 40.00 | 29.15 | 25.97 | 27.41 | 31.48 | 34.85 | 29.48 | 26.64 |
| hsa-miR-379* | 40.00 | 40.00 | 40.00 | 37.18 | 40.00 | 34.04 | 34.14 | 40.00 | 40.00 | 36.47 | 40.00 | 40.00 |
| hsa-miR-380 | 36.90 | 33.35 | 40.00 | 40.00 | 40.00 | 38.82 | 36.94 | 34.74 | 38.96 | 37.87 | 40.00 | 39.48 |
| hsa-miR-380* | 28.47 | 28.04 | 30.41 | 29.15 | 30.38 | 26.37 | 26.22 | 29.18 | 27.47 | 27.72 | 28.32 | 30.11 |
| hsa-miR-381 | 34.61 | 36.32 | 33.78 | 40.00 | 31.39 | 32.40 | 29.73 | 27.22 | 40.00 | 31.19 | 40.00 | 30.51 |
| hsa-miR-382 | 25.99 | 22.07 | 29.47 | 21.31 | 35.70 | 23.01 | 23.70 | 23.94 | 32.36 | 30.56 | 28.07 | 21.63 |
| hsa-miR-383 | 27.25 | 22.89 | 28.56 | 28.82 | 21.04 | 23.72 | 20.21 | 32.98 | 35.04 | 21.24 | 26.30 | 19.76 |
| hsa-miR-384 | 40.00 | 40.00 | 40.00 | 40.00 | 40.00 | 40.00 | 40.00 | 40.00 | 40.00 | 40.00 | 40.00 | 40.00 |
| hsa-miR-409-3p | 22.60 | 21.75 | 25.84 | 20.06 | 26.39 | 20.47 | 21.29 | 23.23 | 40.00 | 24.61 | 25.09 | 22.05 |
| hsa-miR-409-3p | 22.68 | 21.92 | 25.84 | 20.09 | 26.62 | 20.36 | 21.48 | 23.27 | 40.00 | 25.02 | 25.44 | 22.06 |
| hsa-miR-409-5p | 40.00 | 29.10 | 40.00 | 31.31 | 40.00 | 30.59 | 29.68 | 33.60 | 40.00 | 40.00 | 40.00 | 31.03 |
| hsa-miR-410 | 28.19 | 26.86 | 31.79 | 26.45 | 30.98 | 27.19 | 26.60 | 27.60 | 29.90 | 30.59 | 31.27 | 26.14 |
| hsa-miR-411 | 28.70 | 26.73 | 30.81 | 28.19 | 40.00 | 29.03 | 27.12 | 26.63 | 31.18 | 30.06 | 30.96 | 28.05 |
| hsa-miR-411* | 40.00 | 40.00 | 40.00 | 35.32 | 40.00 | 40.00 | 34.25 | 40.00 | 40.00 | 40.00 | 40.00 | 40.00 |
| hsa-miR-412 | 37.80 | 39.24 | 40.00 | 40.00 | 40.00 | 35.39 | 40.00 | 40.00 | 40.00 | 40.00 | 40.00 | 40.00 |
| hsa-miR-422a | 31.57 | 28.88 | 35.30 | 29.15 | 33.65 | 30.04 | 28.62 | 29.06 | 29.02 | 29.64 | 32.10 | 29.82 |
| hsa-miR-423-5p | 26.72 | 26.25 | 26.83 | 25.34 | 26.02 | 28.04 | 26.53 | 25.02 | 28.01 | 27.54 | 25.82 | 26.05 |
| hsa-miR-424 | 40.00 | 36.76 | 40.00 | 40.00 | 40.00 | 40.00 | 31.15 | 40.00 | 40.00 | 33.27 | 40.00 | 40.00 |
| hsa-miR-424* | 27.68 | 31.61 | 32.89 | 30.50 | 34.94 | 27.57 | 30.79 | 34.57 | 32.71 | 29.69 | 34.16 | 40.00 |
| hsa-miR-425 | 28.84 | 26.46 | 29.57 | 29.64 | 28.64 | 28.49 | 25.34 | 27.78 | 28.60 | 29.49 | 27.55 | 28.82 |
| hsa-miR-425* | 28.30 | 26.68 | 33.67 | 28.29 | 28.20 | 27.80 | 25.65 | 28.31 | 27.92 | 27.38 | 29.80 | 28.15 |
| hsa-miR-429 | 40.00 | 36.86 | 36.67 | 40.00 | 40.00 | 40.00 | 38.24 | 36.54 | 40.00 | 40.00 | 40.00 | 40.00 |
| hsa-miR-431 | 40.00 | 35.89 | 40.00 | 31.74 | 40.00 | 33.31 | 29.36 | 34.21 | 40.00 | 40.00 | 40.00 | 31.29 |
| hsa-miR-431* | 30.48 | 33.21 | 38.20 | 28.25 | 40.00 | 29.03 | 34.65 | 36.03 | 40.00 | 40.00 | 39.58 | 30.90 |
| hsa-miR-432 | 22.30 | 22.99 | 24.74 | 20.87 | 27.53 | 20.50 | 22.92 | 23.39 | 30.46 | 40.00 | 24.72 | 22.25 |
| hsa-miR-432 | 22.21 | 22.97 | 24.62 | 20.95 | 27.47 | 20.57 | 22.83 | 23.65 | 30.88 | 40.00 | 24.74 | 22.22 |
| hsa-miR-432* | 31.90 | 31.42 | 35.42 | 33.28 | 40.00 | 31.32 | 30.39 | 34.94 | 37.04 | 32.10 | 37.25 | 38.60 |
| hsa-miR-433 | 22.72 | 22.30 | 24.89 | 21.68 | 31.61 | 21.50 | 24.83 | 22.65 | 29.35 | 29.64 | 26.34 | 20.83 |
| hsa-miR-448 | 40.00 | 40.00 | 40.00 | 40.00 | 40.00 | 40.00 | 40.00 | 40.00 | 40.00 | 40.00 | 40.00 | 40.00 |
| hsa-miR-449a | 40.00 | 33.39 | 40.00 | 40.00 | 40.00 | 40.00 | 36.82 | 37.01 | 40.00 | 36.51 | 40.00 | 40.00 |
| hsa-miR-449b | 40.00 | 40.00 | 40.00 | 40.00 | 31.68 | 40.00 | 36.90 | 40.00 | 40.00 | 40.00 | 40.00 | 40.00 |
| hsa-miR-450a | 40.00 | 36.42 | 40.00 | 32.43 | 40.00 | 40.00 | 40.00 | 40.00 | 38.04 | 40.00 | 40.00 | 40.00 |
| hsa-miR-450b-3p | 40.00 | 32.17 | 40.00 | 40.00 | 40.00 | 40.00 | 40.00 | 40.00 | 40.00 | 40.00 | 40.00 | 40.00 |
| hsa-miR-450b-5p | 40.00 | 32.11 | 38.33 | 32.33 | 40.00 | 40.00 | 40.00 | 37.21 | 38.47 | 33.07 | 37.12 | 39.94 |
| hsa-miR-451 | 30.85 | 24.73 | 27.61 | 28.02 | 25.87 | 28.41 | 22.47 | 24.46 | 31.07 | 26.89 | 23.28 | 27.14 |
| hsa-miR-452 | 32.00 | 32.22 | 34.91 | 40.00 | 32.81 | 40.00 | 29.09 | 40.00 | 40.00 | 31.40 | 35.59 | 40.00 |
| hsa-miR-452* | 35.31 | 40.00 | 37.09 | 40.00 | 40.00 | 33.09 | 31.57 | 40.00 | 37.99 | 33.38 | 40.00 | 40.00 |
| hsa-miR-453 | 38.24 | 39.99 | 40.00 | 31.22 | 40.00 | 34.44 | 40.00 | 40.00 | 40.00 | 40.00 | 37.45 | 40.00 |
| hsa-miR-454 | 28.80 | 24.66 | 27.02 | 25.53 | 26.75 | 27.44 | 22.61 | 25.85 | 25.91 | 26.51 | 25.78 | 26.58 |
| hsa-miR-454* | 40.00 | 40.00 | 40.00 | 34.73 | 40.00 | 40.00 | 29.82 | 40.00 | 40.00 | 33.61 | 40.00 | 40.00 |
| hsa-miR-455-3p | 25.32 | 27.06 | 27.42 | 29.03 | 26.68 | 26.72 | 25.89 | 26.61 | 27.94 | 26.98 | 28.85 | 30.87 |
| hsa-miR-455-5p | 31.32 | 31.69 | 30.55 | 37.31 | 38.54 | 36.64 | 28.43 | 31.57 | 30.26 | 32.18 | 31.99 | 40.00 |
| hsa-miR-483-5p | 24.68 | 25.75 | 24.99 | 25.81 | 24.86 | 25.27 | 24.39 | 22.85 | 27.39 | 20.27 | 24.89 | 25.38 |
| hsa-miR-484 | 21.07 | 19.44 | 22.74 | 19.63 | 19.80 | 19.29 | 19.75 | 19.77 | 21.73 | 20.68 | 21.42 | 19.05 |
| hsa-miR-485-3p | 23.93 | 23.71 | 26.19 | 21.11 | 29.47 | 22.23 | 25.27 | 22.99 | 29.53 | 27.18 | 26.04 | 21.20 |
| hsa-miR-485-5p | 40.00 | 29.33 | 40.00 | 25.82 | 40.00 | 31.43 | 40.00 | 30.18 | 40.00 | 40.00 | 40.00 | 25.70 |
| hsa-miR-486-3p | 30.93 | 31.59 | 31.99 | 32.90 | 31.50 | 31.66 | 31.82 | 29.85 | 32.59 | 32.56 | 27.75 | 30.29 |
| hsa-miR-486-5p | 34.59 | 27.97 | 29.73 | 28.45 | 26.55 | 27.38 | 27.77 | 25.86 | 38.35 | 29.93 | 24.20 | 26.93 |
| hsa-miR-487a | 29.97 | 27.66 | 31.36 | 27.30 | 29.51 | 29.27 | 29.06 | 29.03 | 31.52 | 29.27 | 30.33 | 29.55 |
| hsa-miR-487b | 27.00 | 24.14 | 30.65 | 22.37 | 30.16 | 25.13 | 25.57 | 25.89 | 34.65 | 28.94 | 29.13 | 22.88 |
| hsa-miR-488 | 33.27 | 40.00 | 40.00 | 40.00 | 31.72 | 40.00 | 30.43 | 40.00 | 40.00 | 29.84 | 34.00 | 32.02 |
| hsa-miR-488* | 40.00 | 40.00 | 40.00 | 36.72 | 35.68 | 40.00 | 30.98 | 40.00 | 40.00 | 32.83 | 40.00 | 40.00 |
| hsa-miR-489 | 33.03 | 30.95 | 35.71 | 32.27 | 34.88 | 34.72 | 33.16 | 32.64 | 32.99 | 33.32 | 32.87 | 31.19 |
| hsa-miR-490-3p | 36.53 | 26.84 | 35.12 | 32.64 | 25.05 | 32.91 | 28.15 | 36.22 | 35.34 | 40.00 | 37.57 | 27.06 |
| hsa-miR-491-3p | 40.00 | 40.00 | 40.00 | 40.00 | 40.00 | 40.00 | 40.00 | 40.00 | 40.00 | 40.00 | 40.00 | 40.00 |
| hsa-miR-491-5p | 24.72 | 23.50 | 26.92 | 23.71 | 21.98 | 24.94 | 25.17 | 25.55 | 26.68 | 23.24 | 28.23 | 23.97 |
| hsa-miR-492 | 38.14 | 31.30 | 40.00 | 40.00 | 40.00 | 40.00 | 40.00 | 40.00 | 40.00 | 40.00 | 40.00 | 40.00 |
| hsa-miR-493 | 29.75 | 30.91 | 31.77 | 27.78 | 40.00 | 29.70 | 28.02 | 27.69 | 31.77 | 31.87 | 30.05 | 28.50 |
| hsa-miR-493* | 32.48 | 30.49 | 40.00 | 29.72 | 32.79 | 30.68 | 29.20 | 32.14 | 40.00 | 28.51 | 40.00 | 29.79 |
| hsa-miR-494 | 24.43 | 25.06 | 26.29 | 25.33 | 26.02 | 25.45 | 25.59 | 24.34 | 25.00 | 26.05 | 27.37 | 26.15 |
| hsa-miR-495 | 28.16 | 25.71 | 31.73 | 24.48 | 40.00 | 26.44 | 24.48 | 25.54 | 40.00 | 29.62 | 28.74 | 24.15 |
| hsa-miR-496 | 40.00 | 36.92 | 40.00 | 40.00 | 40.00 | 40.00 | 37.68 | 31.43 | 38.86 | 40.00 | 39.67 | 40.00 |
| hsa-miR-497 | 27.86 | 26.78 | 40.00 | 28.98 | 40.00 | 40.00 | 25.54 | 29.34 | 32.63 | 29.40 | 40.00 | 32.07 |
| hsa-miR-497 | 27.69 | 26.90 | 40.00 | 28.88 | 40.00 | 40.00 | 25.45 | 29.34 | 32.66 | 28.81 | 40.00 | 32.04 |
| hsa-miR-497* | 40.00 | 40.00 | 40.00 | 40.00 | 40.00 | 32.68 | 40.00 | 40.00 | 40.00 | 39.46 | 40.00 | 40.00 |
| hsa-miR-498 | 40.00 | 40.00 | 40.00 | 40.00 | 40.00 | 38.18 | 40.00 | 40.00 | 40.00 | 34.21 | 40.00 | 40.00 |
| hsa-miR-499-3p | 40.00 | 38.45 | 40.00 | 40.00 | 40.00 | 40.00 | 40.00 | 40.00 | 40.00 | 40.00 | 40.00 | 40.00 |
| hsa-miR-499-5p | 40.00 | 40.00 | 40.00 | 40.00 | 40.00 | 40.00 | 35.56 | 40.00 | 40.00 | 40.00 | 40.00 | 40.00 |
| hsa-miR-500 | 27.73 | 28.00 | 31.57 | 28.64 | 27.94 | 30.39 | 26.82 | 27.76 | 28.37 | 26.94 | 30.37 | 27.68 |
| hsa-miR-500* | 31.63 | 33.22 | 33.91 | 30.57 | 30.68 | 33.36 | 31.26 | 37.07 | 33.56 | 28.83 | 40.00 | 32.24 |
| hsa-miR-501-3p | 40.00 | 40.00 | 40.00 | 40.00 | 40.00 | 40.00 | 40.00 | 34.49 | 40.00 | 38.05 | 40.00 | 40.00 |
| hsa-miR-501-5p | 26.32 | 24.35 | 28.86 | 27.42 | 26.86 | 27.66 | 23.75 | 25.77 | 29.25 | 25.84 | 26.76 | 27.05 |
| hsa-miR-502-3p | 28.80 | 29.95 | 30.90 | 29.16 | 28.86 | 31.49 | 29.92 | 27.58 | 31.55 | 28.77 | 32.98 | 29.98 |
| hsa-miR-502-5p | 27.48 | 27.10 | 29.88 | 28.88 | 27.97 | 29.41 | 28.64 | 26.43 | 28.85 | 28.09 | 28.42 | 28.38 |
| hsa-miR-503 | 40.00 | 40.00 | 40.00 | 40.00 | 40.00 | 40.00 | 40.00 | 40.00 | 40.00 | 40.00 | 40.00 | 40.00 |
| hsa-miR-504 | 29.78 | 26.31 | 32.21 | 25.77 | 25.35 | 25.85 | 26.62 | 29.34 | 40.00 | 26.93 | 29.96 | 25.84 |
| hsa-miR-505 | 30.74 | 26.28 | 31.31 | 27.87 | 27.38 | 29.15 | 25.73 | 29.11 | 28.83 | 28.72 | 32.71 | 26.20 |
| hsa-miR-505* | 28.76 | 25.15 | 29.42 | 26.74 | 25.37 | 25.86 | 25.48 | 27.93 | 29.23 | 27.23 | 28.70 | 24.80 |
| hsa-miR-506 | 33.13 | 30.38 | 38.93 | 40.00 | 40.00 | 40.00 | 31.79 | 35.01 | 32.04 | 34.84 | 38.74 | 40.00 |
| hsa-miR-507 | 40.00 | 40.00 | 40.00 | 40.00 | 40.00 | 40.00 | 39.90 | 40.00 | 40.00 | 40.00 | 40.00 | 40.00 |
| hsa-miR-508-3p | 40.00 | 31.57 | 40.00 | 40.00 | 40.00 | 40.00 | 40.00 | 40.00 | 40.00 | 32.98 | 40.00 | 40.00 |
| hsa-miR-508-5p | 40.00 | 40.00 | 40.00 | 40.00 | 40.00 | 40.00 | 40.00 | 40.00 | 40.00 | 40.00 | 40.00 | 40.00 |
| hsa-miR-509-3-5p | 40.00 | 40.00 | 40.00 | 40.00 | 40.00 | 40.00 | 40.00 | 40.00 | 40.00 | 40.00 | 40.00 | 40.00 |
| hsa-miR-509-3p | 29.00 | 29.04 | 29.07 | 28.97 | 29.44 | 28.35 | 28.31 | 29.00 | 30.67 | 25.78 | 29.91 | 29.66 |
| hsa-miR-509-5p | 34.11 | 30.76 | 35.10 | 40.00 | 33.59 | 31.47 | 33.17 | 32.33 | 40.00 | 30.16 | 40.00 | 32.08 |
| hsa-miR-510 | 40.00 | 40.00 | 40.00 | 40.00 | 40.00 | 40.00 | 40.00 | 40.00 | 39.87 | 40.00 | 40.00 | 40.00 |
| hsa-miR-511 | 28.78 | 31.13 | 32.41 | 32.22 | 29.54 | 31.12 | 26.43 | 28.35 | 32.42 | 29.68 | 40.00 | 31.89 |
| hsa-miR-512-3p | 40.00 | 33.87 | 40.00 | 40.00 | 34.25 | 36.47 | 28.79 | 40.00 | 40.00 | 34.51 | 40.00 | 40.00 |
| hsa-miR-512-5p | 40.00 | 40.00 | 40.00 | 40.00 | 40.00 | 40.00 | 40.00 | 40.00 | 40.00 | 40.00 | 40.00 | 40.00 |
| hsa-miR-513-3p | 31.23 | 29.94 | 31.46 | 29.99 | 30.85 | 28.85 | 28.29 | 30.01 | 30.28 | 27.57 | 30.00 | 31.12 |
| hsa-miR-513-5p | 40.00 | 32.74 | 36.20 | 39.72 | 34.97 | 40.00 | 34.15 | 40.00 | 33.35 | 40.00 | 40.00 | 40.00 |
| hsa-miR-515-3p | 37.71 | 29.68 | 40.00 | 33.76 | 40.00 | 38.62 | 33.13 | 31.10 | 31.99 | 36.36 | 40.00 | 34.68 |
| hsa-miR-515-5p | 34.73 | 31.22 | 33.74 | 37.56 | 39.48 | 35.22 | 33.30 | 31.25 | 31.67 | 31.68 | 33.25 | 33.89 |
| hsa-miR-516a-3p | 30.61 | 28.57 | 29.68 | 29.35 | 29.37 | 28.60 | 28.32 | 29.44 | 30.11 | 26.31 | 29.23 | 28.71 |
| hsa-miR-516a-5p | 40.00 | 40.00 | 40.00 | 40.00 | 40.00 | 40.00 | 40.00 | 40.00 | 40.00 | 40.00 | 40.00 | 40.00 |
| hsa-miR-516b | 40.00 | 32.85 | 40.00 | 40.00 | 40.00 | 34.17 | 34.85 | 40.00 | 34.91 | 40.00 | 40.00 | 40.00 |
| hsa-miR-517* | 40.00 | 40.00 | 40.00 | 40.00 | 40.00 | 34.97 | 40.00 | 40.00 | 40.00 | 38.94 | 40.00 | 40.00 |
| hsa-miR-517a | 38.77 | 30.62 | 40.00 | 32.58 | 32.76 | 36.83 | 29.54 | 36.57 | 40.00 | 40.00 | 40.00 | 40.00 |
| hsa-miR-517b | 30.17 | 29.53 | 34.35 | 34.16 | 32.80 | 32.99 | 29.57 | 30.92 | 30.80 | 28.77 | 33.40 | 40.00 |
| hsa-miR-517c | 36.05 | 32.18 | 40.00 | 32.65 | 33.49 | 36.53 | 29.99 | 37.73 | 40.00 | 40.00 | 40.00 | 40.00 |
| hsa-miR-518a-3p | 40.00 | 40.00 | 37.22 | 40.00 | 40.00 | 40.00 | 40.00 | 40.00 | 40.00 | 40.00 | 40.00 | 40.00 |
| hsa-miR-518a-5p | 40.00 | 40.00 | 38.98 | 40.00 | 40.00 | 40.00 | 40.00 | 40.00 | 40.00 | 40.00 | 40.00 | 40.00 |
| hsa-miR-518b | 40.00 | 36.88 | 39.88 | 40.00 | 37.75 | 35.42 | 40.00 | 36.52 | 34.54 | 40.00 | 38.39 | 40.00 |
| hsa-miR-518c | 40.00 | 40.00 | 40.00 | 40.00 | 40.00 | 40.00 | 40.00 | 40.00 | 40.00 | 40.00 | 40.00 | 40.00 |
| hsa-miR-518c* | 40.00 | 39.74 | 40.00 | 40.00 | 40.00 | 40.00 | 40.00 | 40.00 | 40.00 | 34.37 | 40.00 | 40.00 |
| hsa-miR-518d-3p | 35.79 | 32.82 | 34.35 | 34.47 | 39.38 | 32.87 | 33.95 | 31.29 | 40.00 | 40.00 | 40.00 | 35.03 |
| hsa-miR-518d-5p | 40.00 | 40.00 | 40.00 | 40.00 | 40.00 | 40.00 | 40.00 | 40.00 | 40.00 | 40.00 | 40.00 | 40.00 |
| hsa-miR-518e | 40.00 | 27.58 | 35.46 | 37.18 | 36.16 | 38.81 | 32.01 | 34.52 | 34.74 | 36.44 | 36.97 | 39.85 |
| hsa-miR-518e* | 40.00 | 40.00 | 40.00 | 40.00 | 40.00 | 40.00 | 40.00 | 40.00 | 40.00 | 40.00 | 40.00 | 40.00 |
| hsa-miR-518f | 30.79 | 25.72 | 30.73 | 31.48 | 30.37 | 28.39 | 28.68 | 28.90 | 27.76 | 27.68 | 30.43 | 29.88 |
| hsa-miR-518f* | 40.00 | 40.00 | 40.00 | 40.00 | 40.00 | 40.00 | 40.00 | 40.00 | 40.00 | 40.00 | 40.00 | 40.00 |
| hsa-miR-519a | 33.88 | 32.54 | 37.59 | 40.00 | 35.68 | 40.00 | 32.93 | 34.01 | 40.00 | 40.00 | 40.00 | 40.00 |
| hsa-miR-519b-3p | 39.63 | 40.00 | 40.00 | 40.00 | 40.00 | 40.00 | 30.03 | 40.00 | 40.00 | 24.42 | 40.00 | 40.00 |
| hsa-miR-519b-3p | 40.00 | 40.00 | 32.07 | 40.00 | 40.00 | 40.00 | 29.42 | 40.00 | 36.83 | 23.87 | 40.00 | 40.00 |
| hsa-miR-519c-3p | 40.00 | 32.56 | 40.00 | 40.00 | 40.00 | 40.00 | 40.00 | 40.00 | 40.00 | 40.00 | 40.00 | 40.00 |
| hsa-miR-519d | 40.00 | 40.00 | 40.00 | 40.00 | 40.00 | 38.49 | 36.12 | 40.00 | 37.04 | 40.00 | 40.00 | 40.00 |
| hsa-miR-519e | 40.00 | 33.39 | 40.00 | 31.61 | 40.00 | 35.13 | 39.37 | 40.00 | 40.00 | 36.67 | 40.00 | 40.00 |
| hsa-miR-519e* | 40.00 | 40.00 | 40.00 | 40.00 | 40.00 | 40.00 | 40.00 | 40.00 | 40.00 | 40.00 | 40.00 | 40.00 |
| hsa-miR-520a-3p | 38.84 | 40.00 | 40.00 | 40.00 | 40.00 | 40.00 | 37.80 | 40.00 | 40.00 | 40.00 | 40.00 | 40.00 |
| hsa-miR-520a-5p | 40.00 | 40.00 | 40.00 | 40.00 | 40.00 | 40.00 | 40.00 | 40.00 | 40.00 | 40.00 | 40.00 | 39.80 |
| hsa-miR-520b | 29.33 | 25.79 | 39.24 | 35.12 | 33.94 | 33.62 | 32.43 | 30.88 | 30.55 | 31.83 | 33.39 | 37.90 |
| hsa-miR-520c-3p | 28.77 | 28.97 | 29.87 | 29.98 | 29.02 | 26.47 | 27.61 | 30.33 | 29.46 | 24.32 | 29.92 | 30.20 |
| hsa-miR-520d-5p | 40.00 | 40.00 | 34.18 | 34.96 | 37.30 | 40.00 | 36.78 | 37.17 | 34.99 | 40.00 | 32.93 | 38.49 |
| hsa-miR-520e | 35.23 | 30.98 | 39.99 | 37.02 | 40.00 | 40.00 | 35.85 | 33.30 | 33.24 | 40.00 | 32.92 | 39.56 |
| hsa-miR-520f | 37.38 | 38.32 | 34.11 | 32.48 | 40.00 | 32.01 | 40.00 | 40.00 | 40.00 | 32.75 | 40.00 | 40.00 |
| hsa-miR-520g | 40.00 | 40.00 | 40.00 | 40.00 | 40.00 | 40.00 | 40.00 | 40.00 | 40.00 | 40.00 | 40.00 | 40.00 |
| hsa-miR-520h | 40.00 | 40.00 | 40.00 | 40.00 | 36.73 | 31.81 | 33.46 | 40.00 | 40.00 | 38.84 | 40.00 | 40.00 |
| hsa-miR-521 | 40.00 | 40.00 | 40.00 | 40.00 | 40.00 | 40.00 | 40.00 | 40.00 | 40.00 | 40.00 | 40.00 | 40.00 |
| hsa-miR-522 | 40.00 | 33.25 | 40.00 | 40.00 | 40.00 | 40.00 | 33.31 | 40.00 | 40.00 | 36.46 | 40.00 | 40.00 |
| hsa-miR-523 | 28.73 | 31.07 | 40.00 | 40.00 | 40.00 | 33.51 | 36.10 | 33.48 | 30.95 | 36.74 | 40.00 | 32.83 |
| hsa-miR-524-3p | 40.00 | 37.62 | 40.00 | 40.00 | 39.06 | 33.69 | 40.00 | 40.00 | 40.00 | 40.00 | 40.00 | 40.00 |
| hsa-miR-524-3p | 40.00 | 40.00 | 35.56 | 40.00 | 40.00 | 32.98 | 40.00 | 40.00 | 37.89 | 40.00 | 35.87 | 40.00 |
| hsa-miR-524-5p | 40.00 | 40.00 | 40.00 | 40.00 | 40.00 | 40.00 | 40.00 | 40.00 | 40.00 | 40.00 | 40.00 | 40.00 |
| hsa-miR-525-3p | 40.00 | 40.00 | 40.00 | 40.00 | 40.00 | 40.00 | 40.00 | 40.00 | 40.00 | 40.00 | 40.00 | 40.00 |
| hsa-miR-525-5p | 40.00 | 40.00 | 40.00 | 37.25 | 40.00 | 40.00 | 40.00 | 40.00 | 40.00 | 40.00 | 40.00 | 40.00 |
| hsa-miR-526b | 40.00 | 40.00 | 40.00 | 40.00 | 40.00 | 40.00 | 40.00 | 40.00 | 40.00 | 40.00 | 40.00 | 40.00 |
| hsa-miR-526b* | 28.53 | 29.67 | 28.97 | 29.86 | 30.09 | 27.13 | 28.58 | 28.13 | 28.78 | 27.76 | 28.90 | 29.23 |
| hsa-miR-532-3p | 21.62 | 22.96 | 23.58 | 22.99 | 22.53 | 24.12 | 23.66 | 21.51 | 24.22 | 21.81 | 24.97 | 21.48 |
| hsa-miR-532-5p | 25.98 | 25.42 | 27.80 | 25.69 | 24.81 | 27.87 | 23.59 | 25.38 | 26.93 | 24.08 | 27.71 | 24.81 |
| hsa-miR-539 | 26.24 | 23.87 | 33.01 | 23.32 | 31.63 | 25.00 | 24.22 | 24.62 | 40.00 | 31.09 | 30.10 | 24.19 |
| hsa-miR-541 | 31.58 | 29.14 | 36.33 | 29.99 | 40.00 | 31.25 | 35.74 | 37.11 | 38.84 | 40.00 | 40.00 | 30.69 |
| hsa-miR-541* | 40.00 | 32.63 | 34.77 | 33.97 | 40.00 | 29.32 | 34.46 | 40.00 | 38.97 | 31.79 | 36.55 | 37.30 |
| hsa-miR-542-3p | 29.14 | 31.80 | 35.83 | 34.77 | 31.70 | 33.24 | 30.53 | 31.09 | 34.86 | 31.80 | 34.47 | 35.33 |
| hsa-miR-542-5p | 33.84 | 40.00 | 40.00 | 40.00 | 40.00 | 35.16 | 40.00 | 38.39 | 34.63 | 33.91 | 34.90 | 40.00 |
| hsa-miR-543 | 32.41 | 28.46 | 40.00 | 26.23 | 40.00 | 27.06 | 28.13 | 30.48 | 40.00 | 32.26 | 32.43 | 27.09 |
| hsa-miR-544 | 33.17 | 31.80 | 40.00 | 40.00 | 40.00 | 40.00 | 40.00 | 40.00 | 40.00 | 40.00 | 40.00 | 40.00 |
| hsa-miR-545 | 31.04 | 27.11 | 33.48 | 30.49 | 38.58 | 28.89 | 27.08 | 29.13 | 29.58 | 30.36 | 31.84 | 33.98 |
| hsa-miR-545* | 40.00 | 33.72 | 40.00 | 40.00 | 37.27 | 33.05 | 35.62 | 40.00 | 40.00 | 29.85 | 40.00 | 40.00 |
| hsa-miR-548a-3p | 30.19 | 34.35 | 32.93 | 33.39 | 34.10 | 33.73 | 31.72 | 31.39 | 40.00 | 31.45 | 40.00 | 32.94 |
| hsa-miR-548a-5p | 40.00 | 40.00 | 40.00 | 40.00 | 40.00 | 40.00 | 39.64 | 40.00 | 40.00 | 40.00 | 40.00 | 40.00 |
| hsa-miR-548b-3p | 40.00 | 40.00 | 40.00 | 40.00 | 40.00 | 40.00 | 40.00 | 40.00 | 40.00 | 40.00 | 40.00 | 40.00 |
| hsa-miR-548b-5p | 37.08 | 31.04 | 34.23 | 40.00 | 40.00 | 31.16 | 29.95 | 33.85 | 39.50 | 40.00 | 31.95 | 40.00 |
| hsa-miR-548c-3p | 40.00 | 40.00 | 40.00 | 34.29 | 33.45 | 40.00 | 40.00 | 40.00 | 32.99 | 40.00 | 40.00 | 40.00 |
| hsa-miR-548c-5p | 37.67 | 30.55 | 35.62 | 36.83 | 40.00 | 33.15 | 30.02 | 31.38 | 32.78 | 35.57 | 36.77 | 40.00 |
| hsa-miR-548d-3p | 40.00 | 40.00 | 40.00 | 40.00 | 40.00 | 40.00 | 40.00 | 40.00 | 40.00 | 40.00 | 40.00 | 40.00 |
| hsa-miR-548d-5p | 31.90 | 31.12 | 33.15 | 37.15 | 36.37 | 31.05 | 30.17 | 32.13 | 32.10 | 32.16 | 31.75 | 40.00 |
| hsa-miR-549 | 40.00 | 40.00 | 40.00 | 40.00 | 40.00 | 40.00 | 40.00 | 40.00 | 40.00 | 34.03 | 40.00 | 40.00 |
| hsa-miR-550 | 32.95 | 31.37 | 34.55 | 39.54 | 33.08 | 32.19 | 32.06 | 34.65 | 34.46 | 33.02 | 34.09 | 35.47 |
| hsa-miR-550* | 37.58 | 40.00 | 40.00 | 40.00 | 36.47 | 37.00 | 35.11 | 40.00 | 38.83 | 40.00 | 38.00 | 40.00 |
| hsa-miR-551a | 40.00 | 40.00 | 40.00 | 40.00 | 40.00 | 40.00 | 40.00 | 40.00 | 40.00 | 40.00 | 40.00 | 40.00 |
| hsa-miR-551b | 40.00 | 40.00 | 34.69 | 40.00 | 40.00 | 40.00 | 30.67 | 33.54 | 34.36 | 40.00 | 40.00 | 40.00 |
| hsa-miR-551b* | 37.83 | 34.50 | 39.68 | 37.30 | 35.55 | 32.27 | 29.50 | 36.46 | 40.00 | 33.08 | 34.24 | 36.44 |
| hsa-miR-552 | 40.00 | 40.00 | 40.00 | 40.00 | 40.00 | 40.00 | 40.00 | 40.00 | 40.00 | 40.00 | 40.00 | 40.00 |
| hsa-miR-553 | 40.00 | 40.00 | 40.00 | 40.00 | 40.00 | 31.64 | 40.00 | 40.00 | 40.00 | 37.40 | 40.00 | 40.00 |
| hsa-miR-554 | 40.00 | 40.00 | 40.00 | 40.00 | 37.81 | 35.02 | 40.00 | 40.00 | 40.00 | 32.11 | 40.00 | 40.00 |
| hsa-miR-555 | 40.00 | 36.75 | 40.00 | 40.00 | 40.00 | 29.68 | 33.77 | 35.73 | 40.00 | 28.31 | 40.00 | 40.00 |
| hsa-miR-556-3p | 40.00 | 40.00 | 40.00 | 40.00 | 37.98 | 40.00 | 40.00 | 40.00 | 40.00 | 40.00 | 40.00 | 40.00 |
| hsa-miR-556-5p | 40.00 | 40.00 | 40.00 | 40.00 | 40.00 | 40.00 | 40.00 | 40.00 | 40.00 | 40.00 | 40.00 | 40.00 |
| hsa-miR-557 | 40.00 | 40.00 | 40.00 | 40.00 | 39.68 | 29.84 | 39.32 | 40.00 | 40.00 | 30.87 | 40.00 | 40.00 |
| hsa-miR-558 | 40.00 | 40.00 | 40.00 | 40.00 | 40.00 | 37.40 | 40.00 | 40.00 | 40.00 | 33.36 | 40.00 | 40.00 |
| hsa-miR-559 | 40.00 | 37.99 | 40.00 | 40.00 | 39.70 | 40.00 | 40.00 | 36.39 | 40.00 | 40.00 | 40.00 | 40.00 |
| hsa-miR-559 | 40.00 | 40.00 | 37.45 | 40.00 | 40.00 | 40.00 | 40.00 | 40.00 | 40.00 | 40.00 | 40.00 | 40.00 |
| hsa-miR-561 | 40.00 | 40.00 | 40.00 | 40.00 | 40.00 | 40.00 | 40.00 | 39.50 | 40.00 | 40.00 | 40.00 | 40.00 |
| hsa-miR-562 | 40.00 | 40.00 | 40.00 | 40.00 | 40.00 | 37.60 | 40.00 | 40.00 | 40.00 | 40.00 | 40.00 | 40.00 |
| hsa-miR-562 | 40.00 | 40.00 | 40.00 | 40.00 | 40.00 | 40.00 | 40.00 | 40.00 | 35.52 | 40.00 | 40.00 | 40.00 |
| hsa-miR-563 | 40.00 | 40.00 | 40.00 | 40.00 | 40.00 | 40.00 | 36.66 | 40.00 | 40.00 | 38.51 | 40.00 | 40.00 |
| hsa-miR-564 | 28.49 | 27.81 | 30.45 | 30.45 | 30.13 | 28.50 | 26.84 | 29.60 | 29.84 | 28.73 | 28.55 | 33.72 |
| hsa-miR-565 | 25.07 | 24.97 | 22.15 | 27.46 | 26.13 | 24.33 | 22.40 | 27.03 | 27.38 | 21.27 | 27.40 | 25.10 |
| hsa-miR-566 | 29.60 | 27.71 | 40.00 | 40.00 | 40.00 | 24.84 | 27.32 | 40.00 | 40.00 | 40.00 | 40.00 | 35.53 |
| hsa-miR-566 | 29.14 | 27.75 | 40.00 | 40.00 | 40.00 | 25.28 | 27.18 | 40.00 | 40.00 | 40.00 | 40.00 | 40.00 |
| hsa-miR-567 | 40.00 | 35.99 | 40.00 | 40.00 | 40.00 | 30.95 | 31.48 | 40.00 | 34.59 | 28.43 | 38.32 | 40.00 |
| hsa-miR-569 | 40.00 | 40.00 | 40.00 | 34.30 | 40.00 | 38.30 | 40.00 | 40.00 | 40.00 | 31.00 | 40.00 | 40.00 |
| hsa-miR-570 | 40.00 | 40.00 | 40.00 | 40.00 | 36.05 | 40.00 | 40.00 | 40.00 | 33.53 | 40.00 | 40.00 | 40.00 |
| hsa-miR-571 | 40.00 | 26.73 | 40.00 | 40.00 | 29.08 | 25.63 | 25.41 | 40.00 | 29.23 | 26.28 | 40.00 | 32.60 |
| hsa-miR-571 | 40.00 | 27.37 | 40.00 | 40.00 | 28.89 | 25.31 | 25.07 | 40.00 | 29.02 | 26.23 | 40.00 | 40.00 |
| hsa-miR-572 | 40.00 | 30.09 | 40.00 | 30.20 | 40.00 | 28.80 | 40.00 | 40.00 | 30.63 | 40.00 | 40.00 | 40.00 |
| hsa-miR-572 | 40.00 | 29.48 | 40.00 | 30.48 | 40.00 | 29.43 | 40.00 | 40.00 | 30.25 | 40.00 | 40.00 | 40.00 |
| hsa-miR-573 | 31.71 | 34.50 | 36.71 | 36.42 | 29.67 | 29.60 | 29.75 | 34.31 | 33.83 | 32.94 | 35.46 | 40.00 |
| hsa-miR-574-3p | 18.73 | 21.61 | 19.98 | 20.89 | 19.86 | 21.91 | 21.03 | 19.83 | 21.11 | 20.31 | 21.89 | 19.55 |
| hsa-miR-575 | 36.36 | 38.58 | 40.00 | 40.00 | 39.62 | 33.33 | 31.59 | 40.00 | 33.58 | 33.09 | 38.42 | 40.00 |
| hsa-miR-576-3p | 30.66 | 32.11 | 32.64 | 30.89 | 30.87 | 33.03 | 29.08 | 31.67 | 30.80 | 30.47 | 32.49 | 29.94 |
| hsa-miR-576-5p | 40.00 | 34.33 | 40.00 | 40.00 | 40.00 | 40.00 | 40.00 | 40.00 | 40.00 | 33.37 | 40.00 | 40.00 |
| hsa-miR-578 | 40.00 | 40.00 | 40.00 | 40.00 | 37.19 | 40.00 | 40.00 | 40.00 | 40.00 | 40.00 | 40.00 | 40.00 |
| hsa-miR-578 | 40.00 | 40.00 | 40.00 | 40.00 | 40.00 | 40.00 | 40.00 | 40.00 | 40.00 | 40.00 | 40.00 | 40.00 |
| hsa-miR-579 | 40.00 | 30.35 | 40.00 | 33.56 | 40.00 | 40.00 | 32.54 | 30.86 | 40.00 | 40.00 | 40.00 | 40.00 |
| hsa-miR-580 | 40.00 | 40.00 | 40.00 | 40.00 | 35.71 | 30.75 | 40.00 | 40.00 | 40.00 | 30.47 | 40.00 | 40.00 |
| hsa-miR-580 | 40.00 | 40.00 | 39.57 | 40.00 | 40.00 | 31.09 | 40.00 | 40.00 | 40.00 | 30.27 | 40.00 | 40.00 |
| hsa-miR-581 | 40.00 | 40.00 | 40.00 | 40.00 | 40.00 | 40.00 | 40.00 | 38.19 | 40.00 | 28.54 | 40.00 | 40.00 |
| hsa-miR-581 | 34.63 | 40.00 | 40.00 | 40.00 | 40.00 | 40.00 | 40.00 | 40.00 | 40.00 | 28.80 | 40.00 | 40.00 |
| hsa-miR-582-3p | 36.88 | 32.06 | 34.76 | 34.09 | 40.00 | 40.00 | 40.00 | 40.00 | 36.06 | 40.00 | 40.00 | 40.00 |
| hsa-miR-582-5p | 40.00 | 40.00 | 40.00 | 40.00 | 40.00 | 40.00 | 40.00 | 40.00 | 40.00 | 40.00 | 40.00 | 40.00 |
| hsa-miR-583 | 35.64 | 40.00 | 40.00 | 40.00 | 39.33 | 36.87 | 35.67 | 40.00 | 40.00 | 34.95 | 40.00 | 40.00 |
| hsa-miR-584 | 33.33 | 40.00 | 40.00 | 40.00 | 40.00 | 33.98 | 40.00 | 40.00 | 40.00 | 40.00 | 40.00 | 35.20 |
| hsa-miR-584 | 33.69 | 40.00 | 40.00 | 40.00 | 40.00 | 33.62 | 40.00 | 40.00 | 40.00 | 40.00 | 40.00 | 34.85 |
| hsa-miR-585 | 40.00 | 40.00 | 40.00 | 40.00 | 40.00 | 37.26 | 40.00 | 40.00 | 40.00 | 31.36 | 40.00 | 40.00 |
| hsa-miR-586 | 34.91 | 33.67 | 35.26 | 35.92 | 31.68 | 33.00 | 31.88 | 36.42 | 40.00 | 30.63 | 36.12 | 33.04 |
| hsa-miR-587 | 39.63 | 40.00 | 40.00 | 40.00 | 40.00 | 33.09 | 37.45 | 40.00 | 40.00 | 35.50 | 40.00 | 40.00 |
| hsa-miR-588 | 40.00 | 40.00 | 40.00 | 40.00 | 40.00 | 37.61 | 40.00 | 40.00 | 40.00 | 36.51 | 40.00 | 40.00 |
| hsa-miR-588 | 40.00 | 40.00 | 40.00 | 40.00 | 40.00 | 40.00 | 40.00 | 40.00 | 40.00 | 35.93 | 40.00 | 40.00 |
| hsa-miR-589 | 31.75 | 29.43 | 35.04 | 31.13 | 29.87 | 30.72 | 31.35 | 31.57 | 40.00 | 32.87 | 32.47 | 31.40 |
| hsa-miR-589* | 40.00 | 31.23 | 40.00 | 39.98 | 39.98 | 30.93 | 29.04 | 40.00 | 37.08 | 40.00 | 40.00 | 38.57 |
| hsa-miR-590-5p | 32.60 | 28.11 | 33.92 | 34.01 | 32.73 | 32.33 | 28.69 | 29.56 | 33.68 | 30.22 | 33.77 | 35.77 |
| hsa-miR-591 | 40.00 | 33.84 | 40.00 | 40.00 | 40.00 | 40.00 | 40.00 | 40.00 | 33.26 | 30.56 | 40.00 | 40.00 |
| hsa-miR-591 | 40.00 | 33.45 | 40.00 | 40.00 | 40.00 | 40.00 | 40.00 | 33.25 | 40.00 | 40.00 | 40.00 | 40.00 |
| hsa-miR-592 | 38.03 | 25.98 | 31.35 | 29.31 | 27.88 | 28.71 | 24.46 | 30.58 | 40.00 | 31.14 | 30.78 | 29.57 |
| hsa-miR-593 | 40.00 | 40.00 | 40.00 | 40.00 | 40.00 | 40.00 | 40.00 | 40.00 | 40.00 | 32.42 | 40.00 | 40.00 |
| hsa-miR-593* | 40.00 | 40.00 | 40.00 | 40.00 | 40.00 | 40.00 | 40.00 | 40.00 | 40.00 | 40.00 | 40.00 | 40.00 |
| hsa-miR-595 | 40.00 | 40.00 | 40.00 | 40.00 | 40.00 | 33.63 | 34.49 | 40.00 | 40.00 | 40.00 | 40.00 | 40.00 |
| hsa-miR-596 | 40.00 | 40.00 | 40.00 | 40.00 | 40.00 | 40.00 | 30.10 | 40.00 | 31.37 | 40.00 | 40.00 | 37.49 |
| hsa-miR-596 | 40.00 | 40.00 | 40.00 | 40.00 | 40.00 | 40.00 | 30.26 | 40.00 | 31.29 | 40.00 | 40.00 | 40.00 |
| hsa-miR-597 | 31.91 | 28.02 | 29.57 | 28.87 | 29.99 | 29.90 | 28.29 | 30.18 | 29.49 | 28.42 | 30.47 | 27.67 |
| hsa-miR-598 | 26.05 | 26.18 | 26.80 | 23.34 | 25.26 | 23.96 | 23.29 | 25.89 | 28.21 | 22.29 | 27.25 | 20.95 |
| hsa-miR-599 | 40.00 | 40.00 | 40.00 | 40.00 | 40.00 | 40.00 | 40.00 | 40.00 | 40.00 | 34.69 | 40.00 | 40.00 |
| hsa-miR-599 | 40.00 | 40.00 | 40.00 | 40.00 | 40.00 | 40.00 | 40.00 | 40.00 | 40.00 | 40.00 | 40.00 | 40.00 |
| hsa-miR-600 | 40.00 | 40.00 | 34.62 | 40.00 | 40.00 | 40.00 | 40.00 | 40.00 | 40.00 | 40.00 | 40.00 | 40.00 |
| hsa-miR-600 | 40.00 | 40.00 | 40.00 | 40.00 | 40.00 | 40.00 | 40.00 | 40.00 | 40.00 | 40.00 | 40.00 | 40.00 |
| hsa-miR-601 | 27.74 | 28.94 | 30.24 | 29.49 | 28.52 | 28.22 | 27.07 | 28.95 | 29.08 | 29.68 | 28.99 | 30.77 |
| hsa-miR-603 | 40.00 | 40.00 | 40.00 | 40.00 | 40.00 | 35.06 | 40.00 | 40.00 | 40.00 | 40.00 | 40.00 | 40.00 |
| hsa-miR-604 | 40.00 | 40.00 | 40.00 | 33.91 | 40.00 | 28.80 | 40.00 | 35.52 | 40.00 | 33.15 | 40.00 | 40.00 |
| hsa-miR-604 | 40.00 | 40.00 | 40.00 | 40.00 | 40.00 | 28.78 | 40.00 | 34.95 | 40.00 | 32.80 | 40.00 | 40.00 |
| hsa-miR-605 | 40.00 | 40.00 | 33.33 | 40.00 | 40.00 | 38.14 | 40.00 | 40.00 | 40.00 | 40.00 | 40.00 | 40.00 |
| hsa-miR-605 | 40.00 | 40.00 | 40.00 | 40.00 | 40.00 | 40.00 | 40.00 | 40.00 | 36.36 | 40.00 | 40.00 | 40.00 |
| hsa-miR-606 | 40.00 | 33.42 | 40.00 | 40.00 | 40.00 | 40.00 | 33.42 | 40.00 | 32.36 | 40.00 | 40.00 | 35.65 |
| hsa-miR-606 | 36.39 | 32.90 | 40.00 | 34.22 | 40.00 | 40.00 | 32.81 | 40.00 | 40.00 | 40.00 | 40.00 | 40.00 |
| hsa-miR-607 | 40.00 | 40.00 | 40.00 | 40.00 | 40.00 | 40.00 | 40.00 | 40.00 | 40.00 | 40.00 | 40.00 | 40.00 |
| hsa-miR-607 | 40.00 | 40.00 | 40.00 | 40.00 | 40.00 | 40.00 | 40.00 | 40.00 | 40.00 | 40.00 | 40.00 | 40.00 |
| hsa-miR-608 | 40.00 | 40.00 | 40.00 | 40.00 | 40.00 | 28.03 | 40.00 | 40.00 | 40.00 | 33.01 | 40.00 | 40.00 |
| hsa-miR-608 | 40.00 | 40.00 | 40.00 | 40.00 | 40.00 | 40.00 | 40.00 | 40.00 | 40.00 | 40.00 | 40.00 | 40.00 |
| hsa-miR-609 | 40.00 | 40.00 | 40.00 | 40.00 | 40.00 | 40.00 | 40.00 | 40.00 | 40.00 | 40.00 | 39.78 | 40.00 |
| hsa-miR-609 | 40.00 | 40.00 | 40.00 | 40.00 | 36.20 | 40.00 | 40.00 | 40.00 | 40.00 | 40.00 | 39.38 | 40.00 |
| hsa-miR-610 | 27.82 | 27.78 | 28.31 | 27.82 | 27.62 | 25.99 | 27.03 | 27.28 | 28.47 | 25.84 | 28.98 | 28.86 |
| hsa-miR-612 | 40.00 | 40.00 | 40.00 | 40.00 | 40.00 | 39.21 | 40.00 | 40.00 | 40.00 | 34.73 | 40.00 | 40.00 |
| hsa-miR-613 | 40.00 | 39.37 | 40.00 | 40.00 | 40.00 | 32.52 | 40.00 | 40.00 | 40.00 | 40.00 | 40.00 | 40.00 |
| hsa-miR-613 | 40.00 | 40.00 | 40.00 | 40.00 | 40.00 | 32.24 | 40.00 | 40.00 | 40.00 | 36.26 | 40.00 | 40.00 |
| hsa-miR-614 | 40.00 | 40.00 | 40.00 | 40.00 | 40.00 | 40.00 | 40.00 | 38.15 | 40.00 | 40.00 | 40.00 | 40.00 |
| hsa-miR-614 | 36.43 | 38.28 | 40.00 | 40.00 | 40.00 | 40.00 | 31.93 | 40.00 | 40.00 | 31.91 | 40.00 | 40.00 |
| hsa-miR-615-3p | 34.18 | 30.01 | 31.69 | 27.83 | 36.24 | 31.91 | 36.08 | 38.34 | 40.00 | 30.84 | 40.00 | 27.76 |
| hsa-miR-615-5p | 35.25 | 40.00 | 40.00 | 33.47 | 40.00 | 40.00 | 40.00 | 40.00 | 40.00 | 38.88 | 40.00 | 40.00 |
| hsa-miR-616 | 40.00 | 40.00 | 38.53 | 39.86 | 40.00 | 36.36 | 40.00 | 40.00 | 38.47 | 40.00 | 40.00 | 40.00 |
| hsa-miR-616* | 40.00 | 40.00 | 40.00 | 40.00 | 40.00 | 40.00 | 40.00 | 40.00 | 40.00 | 34.45 | 40.00 | 40.00 |
| hsa-miR-617 | 40.00 | 40.00 | 40.00 | 40.00 | 40.00 | 40.00 | 38.52 | 40.00 | 40.00 | 33.28 | 40.00 | 40.00 |
| hsa-miR-618 | 30.54 | 28.75 | 31.74 | 29.63 | 31.26 | 30.28 | 29.77 | 30.72 | 30.03 | 30.79 | 33.36 | 31.53 |
| hsa-miR-619 | 40.00 | 40.00 | 40.00 | 40.00 | 40.00 | 38.39 | 40.00 | 40.00 | 40.00 | 40.00 | 40.00 | 40.00 |
| hsa-miR-621 | 40.00 | 40.00 | 40.00 | 40.00 | 40.00 | 37.40 | 40.00 | 40.00 | 40.00 | 40.00 | 40.00 | 40.00 |
| hsa-miR-621 | 40.00 | 40.00 | 40.00 | 40.00 | 40.00 | 40.00 | 40.00 | 40.00 | 40.00 | 38.27 | 40.00 | 40.00 |
| hsa-miR-622 | 40.00 | 36.32 | 40.00 | 40.00 | 40.00 | 40.00 | 40.00 | 40.00 | 40.00 | 35.14 | 40.00 | 40.00 |
| hsa-miR-622 | 40.00 | 40.00 | 40.00 | 40.00 | 40.00 | 40.00 | 40.00 | 40.00 | 40.00 | 35.18 | 40.00 | 40.00 |
| hsa-miR-623 | 33.00 | 40.00 | 40.00 | 33.68 | 40.00 | 40.00 | 30.41 | 40.00 | 40.00 | 40.00 | 40.00 | 40.00 |
| hsa-miR-623 | 33.31 | 40.00 | 40.00 | 33.56 | 40.00 | 40.00 | 30.49 | 38.46 | 40.00 | 40.00 | 40.00 | 40.00 |
| hsa-miR-624 | 40.00 | 40.00 | 40.00 | 40.00 | 40.00 | 40.00 | 40.00 | 40.00 | 40.00 | 40.00 | 40.00 | 40.00 |
| hsa-miR-624* | 33.65 | 31.26 | 32.49 | 32.76 | 34.36 | 30.85 | 29.63 | 35.72 | 31.54 | 30.31 | 33.32 | 36.56 |
| hsa-miR-625 | 31.60 | 28.68 | 32.36 | 31.31 | 30.75 | 31.16 | 29.15 | 30.54 | 32.75 | 32.28 | 35.37 | 30.64 |
| hsa-miR-625* | 25.50 | 24.72 | 26.06 | 25.94 | 25.22 | 24.44 | 23.39 | 25.59 | 26.14 | 24.46 | 25.31 | 26.63 |
| hsa-miR-626 | 40.00 | 40.00 | 40.00 | 40.00 | 40.00 | 40.00 | 40.00 | 40.00 | 40.00 | 31.24 | 40.00 | 40.00 |
| hsa-miR-626 | 38.63 | 40.00 | 40.00 | 40.00 | 40.00 | 40.00 | 40.00 | 40.00 | 40.00 | 30.86 | 40.00 | 40.00 |
| hsa-miR-627 | 31.58 | 31.65 | 33.50 | 40.00 | 40.00 | 39.21 | 33.26 | 38.72 | 39.58 | 33.81 | 40.00 | 34.92 |
| hsa-miR-628-3p | 37.41 | 40.00 | 40.00 | 40.00 | 40.00 | 40.00 | 40.00 | 40.00 | 40.00 | 31.83 | 40.00 | 40.00 |
| hsa-miR-628-3p | 40.00 | 40.00 | 40.00 | 40.00 | 40.00 | 40.00 | 40.00 | 40.00 | 37.78 | 31.45 | 40.00 | 40.00 |
| hsa-miR-628-5p | 18.99 | 18.53 | 18.48 | 19.16 | 19.35 | 18.98 | 18.45 | 18.23 | 18.50 | 18.23 | 18.87 | 18.18 |
| hsa-miR-629 | 32.34 | 28.68 | 32.97 | 40.00 | 30.86 | 40.00 | 30.86 | 31.71 | 29.26 | 30.19 | 32.16 | 29.91 |
| hsa-miR-629* | 29.65 | 29.68 | 34.79 | 30.13 | 32.18 | 29.60 | 27.12 | 32.51 | 34.73 | 31.07 | 29.29 | 31.77 |
| hsa-miR-630 | 33.72 | 32.51 | 31.64 | 35.05 | 34.62 | 29.84 | 30.06 | 34.34 | 32.19 | 30.66 | 31.41 | 32.36 |
| hsa-miR-631 | 40.00 | 40.00 | 40.00 | 40.00 | 40.00 | 40.00 | 40.00 | 40.00 | 40.00 | 36.09 | 40.00 | 40.00 |
| hsa-miR-631 | 40.00 | 40.00 | 40.00 | 40.00 | 40.00 | 40.00 | 40.00 | 40.00 | 40.00 | 36.34 | 40.00 | 40.00 |
| hsa-miR-632 | 28.15 | 28.31 | 29.09 | 28.61 | 28.87 | 28.00 | 27.86 | 28.43 | 29.68 | 28.56 | 29.39 | 30.10 |
| hsa-miR-633 | 40.00 | 40.00 | 40.00 | 40.00 | 40.00 | 40.00 | 40.00 | 40.00 | 40.00 | 40.00 | 40.00 | 40.00 |
| hsa-miR-633 | 40.00 | 40.00 | 40.00 | 40.00 | 40.00 | 40.00 | 40.00 | 40.00 | 40.00 | 40.00 | 40.00 | 40.00 |
| hsa-miR-634 | 40.00 | 40.00 | 40.00 | 40.00 | 40.00 | 40.00 | 37.88 | 40.00 | 40.00 | 40.00 | 40.00 | 40.00 |
| hsa-miR-634 | 32.71 | 40.00 | 40.00 | 40.00 | 40.00 | 40.00 | 40.00 | 40.00 | 40.00 | 32.69 | 40.00 | 40.00 |
| hsa-miR-635 | 32.11 | 40.00 | 40.00 | 40.00 | 40.00 | 29.80 | 40.00 | 40.00 | 33.18 | 40.00 | 40.00 | 40.00 |
| hsa-miR-635 | 40.00 | 40.00 | 40.00 | 40.00 | 40.00 | 30.08 | 40.00 | 40.00 | 33.66 | 40.00 | 40.00 | 40.00 |
| hsa-miR-636 | 29.04 | 28.15 | 30.80 | 30.04 | 29.24 | 28.69 | 28.28 | 29.36 | 30.03 | 28.28 | 29.85 | 28.47 |
| hsa-miR-637 | 40.00 | 40.00 | 40.00 | 40.00 | 40.00 | 39.08 | 40.00 | 40.00 | 40.00 | 33.02 | 40.00 | 40.00 |
| hsa-miR-637 | 40.00 | 40.00 | 40.00 | 40.00 | 40.00 | 40.00 | 40.00 | 40.00 | 40.00 | 40.00 | 40.00 | 40.00 |
| hsa-miR-638 | 28.03 | 27.36 | 27.58 | 27.91 | 28.27 | 26.98 | 26.46 | 29.00 | 27.19 | 28.03 | 27.74 | 27.74 |
| hsa-miR-639 | 29.82 | 40.00 | 30.24 | 29.81 | 28.98 | 28.86 | 28.21 | 30.34 | 40.00 | 28.68 | 29.87 | 30.85 |
| hsa-miR-639 | 29.91 | 40.00 | 30.22 | 29.97 | 29.42 | 28.71 | 27.66 | 30.26 | 40.00 | 28.42 | 29.57 | 30.97 |
| hsa-miR-640 | 40.00 | 40.00 | 40.00 | 40.00 | 40.00 | 33.55 | 40.00 | 40.00 | 40.00 | 40.00 | 36.69 | 40.00 |
| hsa-miR-640 | 36.88 | 40.00 | 40.00 | 40.00 | 40.00 | 33.67 | 40.00 | 40.00 | 40.00 | 34.46 | 36.87 | 40.00 |
| hsa-miR-641 | 40.00 | 34.50 | 40.00 | 33.70 | 35.42 | 40.00 | 40.00 | 40.00 | 40.00 | 40.00 | 40.00 | 40.00 |
| hsa-miR-641 | 35.32 | 40.00 | 40.00 | 40.00 | 40.00 | 40.00 | 40.00 | 40.00 | 40.00 | 40.00 | 40.00 | 40.00 |
| hsa-miR-642 | 29.68 | 29.15 | 31.11 | 32.89 | 32.65 | 31.02 | 27.96 | 29.52 | 28.94 | 28.35 | 31.61 | 29.27 |
| hsa-miR-643 | 40.00 | 33.84 | 36.46 | 35.30 | 37.40 | 31.06 | 32.87 | 35.21 | 37.35 | 28.52 | 33.14 | 36.50 |
| hsa-miR-644 | 40.00 | 40.00 | 40.00 | 40.00 | 40.00 | 40.00 | 40.00 | 35.09 | 40.00 | 40.00 | 40.00 | 39.34 |
| hsa-miR-644 | 40.00 | 40.00 | 40.00 | 40.00 | 40.00 | 40.00 | 40.00 | 34.70 | 40.00 | 40.00 | 40.00 | 40.00 |
| hsa-miR-645 | 31.05 | 29.51 | 29.60 | 29.85 | 30.66 | 29.77 | 40.00 | 40.00 | 29.48 | 29.47 | 29.37 | 29.32 |
| hsa-miR-645 | 30.91 | 29.77 | 29.40 | 29.35 | 30.81 | 29.37 | 40.00 | 40.00 | 29.07 | 28.83 | 29.88 | 29.65 |
| hsa-miR-646 | 40.00 | 34.96 | 40.00 | 40.00 | 40.00 | 40.00 | 40.00 | 40.00 | 40.00 | 40.00 | 40.00 | 40.00 |
| hsa-miR-646 | 40.00 | 35.30 | 40.00 | 38.97 | 40.00 | 40.00 | 40.00 | 40.00 | 40.00 | 40.00 | 40.00 | 40.00 |
| hsa-miR-647 | 40.00 | 40.00 | 40.00 | 40.00 | 40.00 | 40.00 | 40.00 | 40.00 | 40.00 | 40.00 | 40.00 | 40.00 |
| hsa-miR-647 | 40.00 | 40.00 | 40.00 | 40.00 | 40.00 | 40.00 | 40.00 | 40.00 | 40.00 | 40.00 | 40.00 | 40.00 |
| hsa-miR-648 | 40.00 | 40.00 | 40.00 | 40.00 | 36.36 | 40.00 | 40.00 | 37.06 | 40.00 | 33.51 | 37.93 | 39.40 |
| hsa-miR-648 | 40.00 | 40.00 | 40.00 | 40.00 | 36.12 | 40.00 | 40.00 | 40.00 | 40.00 | 33.81 | 40.00 | 40.00 |
| hsa-miR-649 | 34.15 | 40.00 | 40.00 | 40.00 | 40.00 | 40.00 | 32.21 | 40.00 | 40.00 | 32.65 | 40.00 | 38.74 |
| hsa-miR-649 | 34.73 | 40.00 | 40.00 | 40.00 | 40.00 | 40.00 | 31.63 | 39.10 | 40.00 | 31.97 | 35.41 | 40.00 |
| hsa-miR-650 | 26.94 | 27.27 | 40.00 | 40.00 | 40.00 | 25.73 | 23.95 | 40.00 | 23.12 | 40.00 | 40.00 | 36.40 |
| hsa-miR-650 | 26.44 | 27.71 | 40.00 | 40.00 | 40.00 | 25.54 | 23.87 | 40.00 | 23.01 | 40.00 | 40.00 | 40.00 |
| hsa-miR-651 | 40.00 | 40.00 | 40.00 | 40.00 | 40.00 | 40.00 | 40.00 | 40.00 | 40.00 | 40.00 | 40.00 | 40.00 |
| hsa-miR-652 | 25.71 | 22.50 | 28.67 | 24.96 | 23.82 | 24.68 | 23.13 | 23.03 | 26.34 | 24.05 | 26.43 | 22.12 |
| hsa-miR-653 | 40.00 | 34.86 | 40.00 | 40.00 | 40.00 | 40.00 | 40.00 | 40.00 | 40.00 | 40.00 | 40.00 | 40.00 |
| hsa-miR-654-3p | 31.65 | 27.77 | 40.00 | 29.54 | 40.00 | 30.53 | 29.13 | 31.01 | 32.37 | 40.00 | 30.57 | 28.03 |
| hsa-miR-654-5p | 32.41 | 25.89 | 40.00 | 26.21 | 40.00 | 26.33 | 30.00 | 28.95 | 38.31 | 37.84 | 37.52 | 25.89 |
| hsa-miR-655 | 29.22 | 27.03 | 40.00 | 29.68 | 33.13 | 31.83 | 28.19 | 29.32 | 31.35 | 33.37 | 35.37 | 29.46 |
| hsa-miR-656 | 40.00 | 40.00 | 40.00 | 40.00 | 40.00 | 40.00 | 28.74 | 33.57 | 33.85 | 40.00 | 40.00 | 40.00 |
| hsa-miR-656 | 40.00 | 40.00 | 40.00 | 40.00 | 40.00 | 40.00 | 29.28 | 40.00 | 40.00 | 40.00 | 32.63 | 40.00 |
| hsa-miR-657 | 40.00 | 40.00 | 40.00 | 40.00 | 38.08 | 40.00 | 32.23 | 40.00 | 38.80 | 40.00 | 40.00 | 40.00 |
| hsa-miR-657 | 37.29 | 40.00 | 35.35 | 40.00 | 40.00 | 40.00 | 32.84 | 40.00 | 40.00 | 40.00 | 40.00 | 40.00 |
| hsa-miR-658 | 40.00 | 40.00 | 40.00 | 40.00 | 40.00 | 34.44 | 40.00 | 40.00 | 40.00 | 40.00 | 40.00 | 40.00 |
| hsa-miR-658 | 40.00 | 40.00 | 40.00 | 40.00 | 40.00 | 40.00 | 40.00 | 40.00 | 40.00 | 40.00 | 40.00 | 40.00 |
| hsa-miR-659 | 28.98 | 28.37 | 27.40 | 27.87 | 27.95 | 29.66 | 27.60 | 29.50 | 28.10 | 26.83 | 27.74 | 27.48 |
| hsa-miR-660 | 28.44 | 27.90 | 33.17 | 29.87 | 29.23 | 33.90 | 25.91 | 27.82 | 29.69 | 26.73 | 30.84 | 28.44 |
| hsa-miR-661 | 27.15 | 28.23 | 28.02 | 28.93 | 28.19 | 27.04 | 26.60 | 30.18 | 26.75 | 26.91 | 29.10 | 31.40 |
| hsa-miR-662 | 40.00 | 36.82 | 40.00 | 34.70 | 40.00 | 40.00 | 40.00 | 40.00 | 40.00 | 31.94 | 40.00 | 38.45 |
| hsa-miR-662 | 34.91 | 40.00 | 40.00 | 34.09 | 40.00 | 40.00 | 40.00 | 40.00 | 40.00 | 32.27 | 40.00 | 40.00 |
| hsa-miR-668 | 28.19 | 27.44 | 33.94 | 24.83 | 40.00 | 24.94 | 40.00 | 31.42 | 40.00 | 40.00 | 40.00 | 26.34 |
| hsa-miR-668 | 28.08 | 27.26 | 33.29 | 24.88 | 40.00 | 25.32 | 40.00 | 31.67 | 40.00 | 40.00 | 39.45 | 26.44 |
| hsa-miR-671-3p | 25.82 | 25.19 | 27.29 | 25.15 | 24.68 | 27.07 | 26.76 | 25.97 | 27.65 | 24.76 | 29.03 | 25.57 |
| hsa-miR-672 | 34.10 | 32.57 | 36.57 | 36.62 | 32.34 | 36.04 | 32.61 | 32.21 | 35.74 | 40.00 | 31.18 | 40.00 |
| hsa-miR-674 | 33.08 | 39.68 | 40.00 | 40.00 | 40.00 | 40.00 | 40.00 | 40.00 | 37.31 | 40.00 | 40.00 | 40.00 |
| hsa-miR-675 | 31.24 | 40.00 | 40.00 | 40.00 | 40.00 | 40.00 | 40.00 | 40.00 | 40.00 | 26.19 | 40.00 | 40.00 |
| hsa-miR-675 | 31.61 | 40.00 | 40.00 | 40.00 | 38.25 | 40.00 | 40.00 | 35.97 | 40.00 | 25.76 | 36.97 | 40.00 |
| hsa-miR-7 | 40.00 | 26.42 | 31.71 | 30.01 | 26.89 | 40.00 | 24.78 | 27.15 | 40.00 | 24.53 | 40.00 | 28.13 |
| hsa-miR-7 | 40.00 | 26.48 | 31.22 | 30.26 | 27.25 | 40.00 | 25.21 | 27.42 | 40.00 | 25.16 | 40.00 | 28.82 |
| hsa-miR-708 | 24.52 | 24.77 | 28.91 | 26.96 | 27.08 | 27.53 | 22.19 | 26.39 | 26.47 | 25.74 | 26.39 | 25.59 |
| hsa-miR-708* | 40.00 | 40.00 | 40.00 | 40.00 | 40.00 | 34.84 | 40.00 | 40.00 | 40.00 | 35.46 | 40.00 | 40.00 |
| hsa-miR-7-1* | 35.08 | 27.13 | 33.87 | 27.80 | 27.60 | 27.20 | 24.05 | 31.41 | 33.14 | 26.77 | 32.72 | 28.43 |
| hsa-miR-7-2* | 40.00 | 29.71 | 40.00 | 40.00 | 32.66 | 34.18 | 27.57 | 36.37 | 40.00 | 29.40 | 40.00 | 32.30 |
| hsa-miR-744 | 22.94 | 22.03 | 24.46 | 21.60 | 20.76 | 22.05 | 22.58 | 22.38 | 25.07 | 22.32 | 24.94 | 21.29 |
| hsa-miR-744* | 34.66 | 30.94 | 36.00 | 32.74 | 31.51 | 33.03 | 28.64 | 32.89 | 40.00 | 32.14 | 33.90 | 33.88 |
| hsa-miR-758 | 29.40 | 27.98 | 38.22 | 28.67 | 40.00 | 28.39 | 28.24 | 30.93 | 35.80 | 33.19 | 32.31 | 29.39 |
| hsa-miR-760 | 25.77 | 24.12 | 25.01 | 24.64 | 24.21 | 23.43 | 23.55 | 25.08 | 26.00 | 24.00 | 24.66 | 25.19 |
| hsa-miR-766 | 25.15 | 23.21 | 27.60 | 22.29 | 25.05 | 22.34 | 21.66 | 24.87 | 40.00 | 22.96 | 25.94 | 23.41 |
| hsa-miR-766 | 25.26 | 23.34 | 27.65 | 22.32 | 25.01 | 22.52 | 21.83 | 24.91 | 40.00 | 23.14 | 25.76 | 23.52 |
| hsa-miR-767-3p | 40.00 | 40.00 | 40.00 | 40.00 | 40.00 | 40.00 | 40.00 | 40.00 | 40.00 | 40.00 | 40.00 | 40.00 |
| hsa-miR-767-3p | 40.00 | 40.00 | 40.00 | 40.00 | 40.00 | 40.00 | 40.00 | 40.00 | 40.00 | 40.00 | 40.00 | 31.60 |
| hsa-miR-767-5p | 40.00 | 25.94 | 38.94 | 26.84 | 28.98 | 40.00 | 26.61 | 40.00 | 40.00 | 40.00 | 29.89 | 26.43 |
| hsa-miR-767-5p | 40.00 | 25.99 | 40.00 | 26.62 | 28.68 | 40.00 | 26.70 | 40.00 | 40.00 | 40.00 | 30.13 | 25.78 |
| hsa-miR-768-3p | 18.34 | 17.95 | 19.93 | 18.30 | 16.44 | 18.05 | 18.47 | 17.74 | 19.79 | 17.97 | 20.54 | 17.12 |
| hsa-miR-768-3p | 18.36 | 18.07 | 19.97 | 18.41 | 16.47 | 18.01 | 18.56 | 17.89 | 19.86 | 18.22 | 20.51 | 17.19 |
| hsa-miR-769-3p | 40.00 | 29.44 | 40.00 | 28.55 | 40.00 | 40.00 | 40.00 | 40.00 | 40.00 | 28.61 | 40.00 | 30.22 |
| hsa-miR-769-3p | 40.00 | 29.42 | 40.00 | 28.10 | 40.00 | 40.00 | 40.00 | 40.00 | 40.00 | 29.18 | 40.00 | 29.62 |
| hsa-miR-769-5p | 28.48 | 40.00 | 29.64 | 24.03 | 24.31 | 25.30 | 25.45 | 40.00 | 30.35 | 25.39 | 28.06 | 24.27 |
| hsa-miR-769-5p | 28.52 | 40.00 | 29.84 | 24.35 | 24.50 | 24.93 | 25.51 | 40.00 | 30.58 | 26.05 | 28.71 | 24.73 |
| hsa-miR-770-5p | 24.69 | 26.20 | 28.31 | 25.76 | 34.61 | 23.59 | 26.57 | 27.23 | 32.72 | 29.04 | 28.70 | 27.06 |
| hsa-miR-801 | 21.50 | 40.00 | 23.58 | 22.75 | 23.34 | 24.19 | 20.73 | 25.40 | 21.23 | 21.57 | 25.57 | 25.99 |
| hsa-miR-801 | 21.65 | 40.00 | 23.37 | 22.58 | 23.14 | 24.02 | 20.81 | 25.47 | 21.11 | 21.51 | 25.97 | 26.61 |
| hsa-miR-871 | 40.00 | 30.20 | 33.84 | 40.00 | 40.00 | 40.00 | 40.00 | 40.00 | 40.00 | 40.00 | 39.78 | 40.00 |
| hsa-miR-872 | 37.44 | 40.00 | 34.34 | 40.00 | 40.00 | 40.00 | 37.80 | 37.67 | 38.66 | 37.92 | 40.00 | 40.00 |
| hsa-miR-873 | 40.00 | 40.00 | 40.00 | 40.00 | 33.13 | 34.88 | 30.88 | 40.00 | 40.00 | 40.00 | 40.00 | 40.00 |
| hsa-miR-874 | 26.12 | 27.64 | 30.26 | 26.77 | 25.88 | 27.26 | 27.98 | 26.94 | 29.59 | 26.00 | 31.06 | 27.68 |
| hsa-miR-875-3p | 38.58 | 30.25 | 40.00 | 40.00 | 40.00 | 40.00 | 40.00 | 40.00 | 40.00 | 40.00 | 40.00 | 40.00 |
| hsa-miR-875-5p | 23.64 | 28.76 | 29.41 | 30.04 | 29.98 | 28.16 | 27.57 | 32.26 | 30.85 | 25.44 | 31.18 | 30.56 |
| hsa-miR-876-3p | 40.00 | 31.53 | 40.00 | 32.75 | 40.00 | 40.00 | 30.02 | 40.00 | 40.00 | 30.89 | 40.00 | 40.00 |
| hsa-miR-876-5p | 40.00 | 40.00 | 40.00 | 40.00 | 40.00 | 40.00 | 34.40 | 40.00 | 40.00 | 37.12 | 40.00 | 40.00 |
| hsa-miR-877 | 25.81 | 24.84 | 25.82 | 25.19 | 24.88 | 24.33 | 24.33 | 26.02 | 26.14 | 25.80 | 25.89 | 24.81 |
| hsa-miR-885-3p | 38.65 | 27.55 | 40.00 | 38.40 | 40.00 | 40.00 | 40.00 | 36.31 | 35.48 | 33.10 | 39.15 | 40.00 |
| hsa-miR-885-5p | 26.68 | 23.78 | 28.25 | 23.42 | 23.74 | 25.59 | 23.62 | 26.79 | 32.54 | 24.02 | 26.80 | 24.29 |
| hsa-miR-886-3p | 22.99 | 26.42 | 23.25 | 27.10 | 27.00 | 25.55 | 24.41 | 23.37 | 23.80 | 22.75 | 25.57 | 30.57 |
| hsa-miR-886-5p | 24.04 | 26.85 | 24.51 | 26.60 | 27.02 | 25.53 | 24.47 | 25.70 | 24.49 | 26.63 | 25.40 | 30.92 |
| hsa-miR-887 | 40.00 | 40.00 | 40.00 | 40.00 | 40.00 | 40.00 | 40.00 | 40.00 | 40.00 | 40.00 | 40.00 | 40.00 |
| hsa-miR-888 | 34.14 | 27.65 | 39.57 | 31.07 | 33.64 | 32.99 | 31.56 | 30.02 | 30.13 | 31.54 | 31.33 | 34.33 |
| hsa-miR-888* | 40.00 | 40.00 | 40.00 | 40.00 | 40.00 | 36.49 | 40.00 | 40.00 | 40.00 | 38.89 | 40.00 | 40.00 |
| hsa-miR-889 | 30.69 | 27.79 | 40.00 | 29.11 | 40.00 | 29.31 | 27.74 | 30.57 | 40.00 | 39.79 | 33.74 | 28.24 |
| hsa-miR-890 | 40.00 | 40.00 | 40.00 | 40.00 | 40.00 | 40.00 | 40.00 | 40.00 | 40.00 | 40.00 | 40.00 | 40.00 |
| hsa-miR-891a | 40.00 | 40.00 | 40.00 | 40.00 | 40.00 | 40.00 | 40.00 | 40.00 | 39.39 | 34.08 | 40.00 | 40.00 |
| hsa-miR-891b | 40.00 | 36.73 | 40.00 | 40.00 | 40.00 | 40.00 | 40.00 | 40.00 | 40.00 | 40.00 | 40.00 | 40.00 |
| hsa-miR-892a | 40.00 | 33.66 | 40.00 | 40.00 | 40.00 | 40.00 | 40.00 | 40.00 | 40.00 | 40.00 | 40.00 | 40.00 |
| hsa-miR-892b | 32.74 | 40.00 | 32.47 | 33.72 | 34.27 | 29.81 | 28.84 | 32.30 | 35.31 | 32.07 | 34.80 | 34.28 |
| hsa-miR-9 | 27.90 | 24.68 | 28.48 | 27.74 | 26.27 | 30.29 | 20.73 | 25.87 | 35.81 | 24.14 | 26.61 | 25.46 |
| hsa-miR-9* | 29.33 | 28.82 | 30.64 | 40.00 | 32.13 | 31.74 | 26.28 | 35.35 | 40.00 | 28.29 | 29.73 | 30.50 |
| hsa-miR-920 | 40.00 | 40.00 | 40.00 | 34.45 | 40.00 | 40.00 | 40.00 | 40.00 | 40.00 | 40.00 | 40.00 | 40.00 |
| hsa-miR-920 | 40.00 | 40.00 | 40.00 | 40.00 | 40.00 | 40.00 | 40.00 | 40.00 | 40.00 | 40.00 | 40.00 | 40.00 |
| hsa-miR-921 | 40.00 | 40.00 | 40.00 | 40.00 | 40.00 | 36.15 | 40.00 | 40.00 | 40.00 | 40.00 | 38.88 | 40.00 |
| hsa-miR-921 | 40.00 | 40.00 | 33.96 | 40.00 | 40.00 | 36.52 | 33.68 | 40.00 | 34.58 | 38.17 | 40.00 | 40.00 |
| hsa-miR-922 | 40.00 | 40.00 | 40.00 | 40.00 | 40.00 | 40.00 | 30.66 | 40.00 | 40.00 | 40.00 | 40.00 | 35.02 |
| hsa-miR-922 | 40.00 | 40.00 | 31.87 | 40.00 | 40.00 | 40.00 | 31.24 | 40.00 | 35.33 | 30.82 | 40.00 | 34.61 |
| hsa-miR-923 | 22.93 | 21.68 | 21.56 | 23.09 | 23.17 | 22.24 | 19.97 | 40.00 | 21.65 | 24.03 | 22.94 | 25.26 |
| hsa-miR-923 | 22.52 | 21.15 | 21.37 | 22.88 | 23.10 | 22.29 | 19.71 | 40.00 | 21.14 | 24.42 | 22.45 | 24.84 |
| hsa-miR-924 | 40.00 | 35.33 | 40.00 | 40.00 | 40.00 | 40.00 | 40.00 | 40.00 | 40.00 | 40.00 | 40.00 | 40.00 |
| hsa-miR-924 | 40.00 | 40.00 | 40.00 | 40.00 | 40.00 | 40.00 | 35.35 | 40.00 | 40.00 | 40.00 | 40.00 | 36.24 |
| hsa-miR-92a | 22.16 | 20.32 | 21.87 | 20.99 | 20.86 | 21.38 | 19.23 | 20.24 | 24.10 | 22.12 | 20.15 | 19.33 |
| hsa-miR-92a-1* | 27.77 | 28.01 | 28.34 | 28.88 | 28.19 | 27.61 | 27.24 | 27.61 | 29.62 | 28.61 | 26.17 | 27.45 |
| hsa-miR-92a-2* | 40.00 | 40.00 | 40.00 | 40.00 | 40.00 | 36.29 | 40.00 | 40.00 | 40.00 | 26.31 | 40.00 | 40.00 |
| hsa-miR-92b* | 32.03 | 37.13 | 40.00 | 40.00 | 38.36 | 35.74 | 37.56 | 40.00 | 40.00 | 32.05 | 37.83 | 40.00 |
| hsa-miR-93 | 24.16 | 19.99 | 26.32 | 22.54 | 21.48 | 22.51 | 19.47 | 23.56 | 24.54 | 21.72 | 22.71 | 20.07 |
| hsa-miR-93* | 30.63 | 26.12 | 32.77 | 28.74 | 27.91 | 27.94 | 25.69 | 29.62 | 29.98 | 28.91 | 29.12 | 26.30 |
| hsa-miR-933 | 40.00 | 40.00 | 40.00 | 40.00 | 40.00 | 40.00 | 40.00 | 40.00 | 40.00 | 40.00 | 40.00 | 40.00 |
| hsa-miR-933 | 40.00 | 40.00 | 40.00 | 40.00 | 40.00 | 40.00 | 40.00 | 40.00 | 40.00 | 40.00 | 40.00 | 40.00 |
| hsa-miR-934 | 40.00 | 40.00 | 40.00 | 40.00 | 38.91 | 33.79 | 40.00 | 40.00 | 40.00 | 40.00 | 40.00 | 40.00 |
| hsa-miR-934 | 40.00 | 40.00 | 40.00 | 40.00 | 40.00 | 40.00 | 40.00 | 40.00 | 40.00 | 40.00 | 40.00 | 40.00 |
| hsa-miR-935 | 40.00 | 40.00 | 40.00 | 40.00 | 40.00 | 40.00 | 40.00 | 28.29 | 40.00 | 40.00 | 40.00 | 40.00 |
| hsa-miR-935 | 40.00 | 40.00 | 40.00 | 40.00 | 40.00 | 40.00 | 40.00 | 28.38 | 40.00 | 40.00 | 40.00 | 40.00 |
| hsa-miR-936 | 40.00 | 40.00 | 40.00 | 40.00 | 40.00 | 40.00 | 40.00 | 40.00 | 40.00 | 36.87 | 40.00 | 40.00 |
| hsa-miR-937 | 31.71 | 33.66 | 34.67 | 38.17 | 40.00 | 32.23 | 30.97 | 40.00 | 40.00 | 34.12 | 40.00 | 32.06 |
| hsa-miR-938 | 40.00 | 38.88 | 40.00 | 40.00 | 40.00 | 32.09 | 40.00 | 36.95 | 40.00 | 40.00 | 40.00 | 37.89 |
| hsa-miR-939 | 29.51 | 28.97 | 32.93 | 31.01 | 32.90 | 27.71 | 26.39 | 32.58 | 29.04 | 29.54 | 30.53 | 33.83 |
| hsa-miR-941 | 37.32 | 29.96 | 39.96 | 30.00 | 31.46 | 31.22 | 29.18 | 27.99 | 36.04 | 29.87 | 32.32 | 30.83 |
| hsa-miR-942 | 33.60 | 30.57 | 38.80 | 36.46 | 31.58 | 32.55 | 27.86 | 34.34 | 33.05 | 31.67 | 29.75 | 32.84 |
| hsa-miR-943 | 40.00 | 40.00 | 40.00 | 40.00 | 40.00 | 40.00 | 38.01 | 40.00 | 40.00 | 40.00 | 40.00 | 40.00 |
| hsa-miR-944 | 40.00 | 40.00 | 40.00 | 40.00 | 40.00 | 37.92 | 40.00 | 40.00 | 40.00 | 32.45 | 40.00 | 40.00 |
| hsa-miR-95 | 26.57 | 22.73 | 27.43 | 23.03 | 23.16 | 24.01 | 22.46 | 25.58 | 29.63 | 22.66 | 28.51 | 23.06 |
| hsa-miR-96 | 40.00 | 40.00 | 40.00 | 40.00 | 40.00 | 40.00 | 40.00 | 40.00 | 40.00 | 40.00 | 40.00 | 40.00 |
| hsa-miR-96* | 40.00 | 38.61 | 40.00 | 40.00 | 35.82 | 35.66 | 40.00 | 40.00 | 40.00 | 35.74 | 40.00 | 40.00 |
| hsa-miR-98 | 40.00 | 35.28 | 40.00 | 40.00 | 38.87 | 40.00 | 31.12 | 34.18 | 40.00 | 36.76 | 40.00 | 40.00 |
| hsa-miR-99a | 24.48 | 25.84 | 26.28 | 26.79 | 25.60 | 27.70 | 22.25 | 25.03 | 27.16 | 25.65 | 26.92 | 26.43 |
| hsa-miR-99a* | 28.76 | 27.97 | 30.56 | 29.41 | 27.85 | 26.64 | 26.82 | 30.77 | 33.24 | 27.06 | 30.94 | 30.86 |
| hsa-miR-99b | 23.37 | 23.37 | 25.84 | 23.00 | 22.13 | 22.46 | 22.06 | 23.45 | 23.94 | 22.83 | 26.08 | 23.00 |
| hsa-miR-99b* | 28.14 | 28.19 | 29.95 | 27.67 | 27.51 | 26.25 | 27.67 | 30.72 | 31.08 | 28.15 | 29.00 | 27.96 |
| MammU6 | 18.07 | 17.00 | 18.72 | 17.63 | 17.38 | 16.87 | 16.57 | 15.81 | 17.99 | 17.37 | 18.33 | 16.85 |
| MammU6 | 17.85 | 16.95 | 18.65 | 17.46 | 17.36 | 16.94 | 16.46 | 15.79 | 17.82 | 17.24 | 18.05 | 16.67 |
| MammU6 | 18.03 | 17.03 | 18.75 | 17.59 | 17.32 | 16.85 | 16.49 | 15.90 | 18.02 | 17.16 | 18.27 | 16.67 |
| MammU6 | 17.79 | 16.98 | 18.74 | 17.66 | 17.26 | 16.79 | 16.41 | 15.75 | 17.96 | 17.30 | 18.07 | 16.61 |
| MammU6 | 17.26 | 16.51 | 18.51 | 17.58 | 16.92 | 16.02 | 15.95 | 16.18 | 17.87 | 17.53 | 17.98 | 16.95 |
| MammU6 | 17.15 | 16.32 | 18.44 | 17.24 | 16.95 | 15.97 | 15.87 | 16.04 | 17.66 | 17.72 | 17.75 | 16.82 |
| MammU6 | 17.39 | 16.54 | 18.63 | 17.57 | 16.82 | 16.11 | 16.03 | 16.32 | 17.91 | 17.66 | 17.90 | 16.84 |
| MammU6 | 17.34 | 16.48 | 18.44 | 17.46 | 16.90 | 15.99 | 15.99 | 16.29 | 17.97 | 17.53 | 17.95 | 16.89 |
| RNU24 | 21.49 | 20.37 | 23.01 | 22.54 | 22.04 | 22.51 | 19.95 | 21.18 | 24.03 | 22.07 | 21.68 | 21.39 |
| RNU24 | 21.44 | 20.27 | 23.04 | 22.41 | 21.97 | 22.34 | 19.98 | 21.29 | 24.10 | 22.04 | 21.67 | 21.40 |
| RNU24 | 21.48 | 20.45 | 22.92 | 22.50 | 21.80 | 22.57 | 20.02 | 21.33 | 23.93 | 40.00 | 21.65 | 21.52 |
| RNU24 | 21.48 | 20.49 | 23.05 | 22.55 | 22.02 | 22.50 | 19.92 | 21.31 | 24.03 | 22.06 | 21.71 | 21.56 |
| RNU43 | 21.99 | 23.11 | 24.59 | 21.90 | 23.66 | 23.34 | 21.76 | 24.90 | 23.14 | 20.96 | 40.00 | 22.49 |
| RNU43 | 21.68 | 23.13 | 40.00 | 22.07 | 23.88 | 22.93 | 21.56 | 24.24 | 23.12 | 21.49 | 40.00 | 22.49 |
| RNU43 | 21.85 | 23.14 | 24.59 | 22.16 | 23.60 | 23.62 | 21.94 | 24.62 | 23.08 | 21.62 | 40.00 | 22.59 |
| RNU43 | 22.03 | 23.17 | 24.60 | 21.99 | 23.75 | 23.31 | 22.31 | 24.27 | 23.43 | 21.34 | 40.00 | 22.61 |
| RNU44 | 19.52 | 17.47 | 20.68 | 18.57 | 18.17 | 19.14 | 18.33 | 18.06 | 20.01 | 17.32 | 19.19 | 16.35 |
| RNU44 | 19.96 | 17.49 | 21.08 | 19.13 | 18.49 | 19.60 | 18.19 | 19.91 | 20.70 | 17.98 | 19.33 | 40.00 |
| RNU44 | 20.28 | 17.56 | 20.62 | 19.09 | 18.48 | 19.32 | 18.27 | 19.88 | 20.65 | 18.17 | 19.34 | 17.37 |
| RNU44 | 20.25 | 17.67 | 20.93 | 18.97 | 18.40 | 19.28 | 18.29 | 19.95 | 20.96 | 18.09 | 19.38 | 17.36 |
| RNU44 | 20.07 | 17.55 | 21.09 | 19.09 | 18.58 | 19.71 | 18.45 | 19.97 | 20.94 | 17.71 | 40.00 | 17.39 |
| RNU48 | 16.84 | 16.04 | 18.30 | 16.07 | 15.88 | 15.89 | 16.93 | 15.10 | 17.77 | 15.66 | 17.49 | 15.56 |
| RNU48 | 40.00 | 40.00 | 18.57 | 16.20 | 15.78 | 15.62 | 16.42 | 15.55 | 17.84 | 16.16 | 17.61 | 16.21 |
| RNU48 | 21.02 | 40.00 | 18.56 | 16.23 | 15.74 | 15.73 | 16.41 | 15.65 | 17.84 | 16.08 | 17.72 | 16.21 |
| RNU48 | 16.57 | 40.00 | 18.53 | 16.25 | 15.83 | 15.76 | 16.40 | 15.64 | 17.80 | 16.17 | 17.70 | 16.28 |
| RNU48 | 17.96 | 40.00 | 18.59 | 16.26 | 15.83 | 15.67 | 16.52 | 15.63 | 17.87 | 16.09 | 17.62 | 16.20 |
| RNU6B | 24.94 | 23.53 | 25.59 | 24.84 | 23.80 | 24.12 | 23.23 | 24.20 | 25.66 | 22.55 | 24.78 | 24.39 |
| RNU6B | 25.31 | 23.50 | 25.42 | 24.89 | 23.86 | 24.34 | 23.45 | 24.34 | 25.55 | 22.77 | 25.00 | 24.29 |
| RNU6B | 25.39 | 23.86 | 25.92 | 24.97 | 23.96 | 24.28 | 23.94 | 24.61 | 25.67 | 22.98 | 24.85 | 24.10 |
| RNU6B | 25.39 | 23.71 | 25.86 | 24.91 | 24.22 | 24.46 | 23.81 | 24.37 | 26.10 | 22.43 | 25.23 | 24.48 |
