# Supplementary material for: miRNA expression profile of bone marrow resident cells from children with neuroblastoma is not significantly different from that of healthy children
Source: Oncotarget. 2018 Apr 10;9(27):19014–25. doi: 10.18632/oncotarget.24874 (PMC5922374; doi:10.18632/oncotarget.24874)
Supplement: Supplementary file 6 [file oncotarget-09-19014-s006.docx]

**Supplementary Table 5:** **List of unique genes potentially targeted by miR-221 extracted from Targetscan, PicTar and MiRanda databases and list of the genes under-expressed by BM resident cells from children with NB, as compared with healthy children**

| **Genes potentially targeted by miR-221** | **Genes under-expressed by NB BM resident cells** |  |
| --- | --- | --- |
| A1A4G5_HUMAN  AADAC  ABCC11  ABCF3  ABHD12  ACCN5  ACOT1  ACOT11  ACOT2  ACTB  ACTR10  ADAM22  ADAMTS13  ADAMTS6  ADAMTSL1  ADARB1  ADD3  ADRA1A  ADSL  AFF4  AGTPBP1  AKAP9  ALDH1A1  ALKBH3  ALKBH8  ANGPT4  ANGPTL2  ANGPTL4  ANKHD1  ANKRD10  ANKRD17  ANKRD31  ANKRD55  ANXA1  ANXA3  AP2A1  AP3B2  APBB1IP  APEH  APOM  APOOL  ARF1  ARF4  ARHGAP19  ARHGEF17  ARID1A  ARIH2  ARMC6  ASPA  ATF2  ATN1  ATP13A2  ATP1A1  ATP1A4  ATP1B1  ATPAF2  ATPBD4  ATXN3L  AUTS2  AZGP1  B3GAT3  B3GNTL1  BBC3  BCAT1  BCHE  BCL2L14  BCOR  BLVRA  BLYM_HUMAN  BMF  BOLL  BRAP  BRI3  BRP44  BRSK2  BTG3  BXDC2  BZW1  C10orf112  C10orf59  C10orf82  C11orf2  C11orf39  C11orf41  C12orf38  C12orf50  C12orf54  C13orf3  C14orf104  C14orf115  C14orf50  C14orf68  C14orf83  C15orf26  C16orf45  C16orf57  C17orf75  C18orf17  C18orf22  C18orf34  C18orf56  C19orf59  C1orf34  C1orf65  C1orf80  C1orf87  C1orf89  C1QTNF9  C20orf116  C20orf119  C20orf19  C20orf23  C20orf72  C21orf58  C21orf82  C3orf10  C3orf37  C3orf49  C4A  C4B  C4orf22  C4orf33  C6  C6orf141  C6orf159  C6orf170  C6orf199  C6orf213  C6orf51  C6orf58  C8orf42  C9orf135  C9orf21  C9orf66  CABYR  CACNA1S  CALB2  CAPN9  CAPRIN2  CAPSL  CASK  CASKIN1  CB031_HUMAN  CBWD1  CBWD2  CBWD3  CBWD5  CBWD6  CBWD7  CCDC123  CCDC129  CCDC15  CCDC18  CCDC36  CCDC47  CCDC50  CCDC64  CCDC66  CCDC71  CCDC99  CCT5  CD180  CD300A  CD40  CD99  CDH23  CDH8  CDK2AP1  CDKN1C  CEACAM5  CENPQ  CENTA1  CEP110  CFLAR  CHD5  CHMP4B  CHRNB3  CLDN14  CLNS1A  CLPTM1  CLRN3  CMTM5  CNDP2  CNOT3  CPA3  CPE  CPO  CRELD2  CRISP3  CROP_HUMAN  CRSP3  CRYGB  CSF1  CSNK1A1  CSNK2A1P  CSNK2A2  CTNNBIP1  CTR9  CTSE  CXorf20  CXorf25  CXorf52  CXorf57  CXorf59  CYP2F1  CYP4A11  CYP4A22  CYP4X1  CYR61  DAOA  DAPK1  DARC  DCAKD  DCDC2  DCLRE1A  DCP2  DDX39  DDX43  DDX56  DEPDC1  DHDDS  DHTKD1  DIP2C  DIRAS3  DLG7  DMRT3  DNAH7  DNAH9  DNAJC14  DNHD2  DNMT1  DNTT  DNTTIP1  DOCK10  DOK3  DOK7  DSPP  DUSP22  DVL2  ECD  EFNA1  EGFLAM  EIF1  EIF3S1  ENOX1  ENTPD6  EPB41L1  EPB41L5  EPHA8  ERAL1  ERAS  EXOC3L  EXOC8  EXOSC8  EXOSC9  F13A1  FAHD1  FAM26A  FAM26B  FAM35A  FAM3B  FAM57A  FAM58B  FANCD2  FANK1  FASTKD2  FBX47_HUMAN  FBXL13  FBXL15  FBXO22  FBXO27  FBXW12  FCGRT  FCRLA  FEZ1  FFAR2  FGL1  FIGN  FOS  FOXA2  FOXJ1  FOXL1  FSHR  FUK  FVT1  FXYD4  FXYD5  FZD9  GABRA1  GABRR2  GALK1  GALNTL4  GAP43  GARNL1  GARNL3  GBGT1  GCKR  GDA  GDF10  GDF5OS  GDF9  GDI2  GDPD1  GJB6  GLDC  GLMN  GNA14  GNAI2  GNAS  GNB3  GNGT2  GNRHR2  GOLGA6  GOLPH3L  GON4L  GPR107  GPR119  GPR137B  GPR143  GPR174  GPRC5B  GRHL1  GRHL3  GRIP2  GRM1  GSDMDC1  GSTA1  GTF2H4  GTF3C5  GUK1  H1FNT  H1FX  HAS3  HAVCR1  HCRTR1  HDAC10  HEATR5B  HECTD2  HERC2P2  HIST1H3H  HIST2H2BA  HIST3H2BB  HLA-F  HMG2L1  HMGA2  HMGCL  HMOX1  HORMAD1  HOXB1  HOXC12  HPSE2  HSFY1  HSPA8  HSPB8  HTR3D  IFIT1L  IFRD1  IFRD2  IFT80  IGF2BP2  IGFBPL1  IGFL2  IGSF2  IKZF3  IL17RB  IL7R  ILF2  INA  INPP4B  IQCH  IRF2  IRX5  ISG20L2  ITGA6  ITGB1BP2  ITGB8  JAG2  JMJD4  KCMF1  KCNE2  KCNG4  KCNIP1  KCNK2  KCNV2  KHDRBS2  KIAA0040  KIAA0515  KIAA1245  KIAA1267  KIAA1324L  KIAA1333  KIAA1370  KIAA1411  KIAA1731  KIF1A  KIF20A  KIR3DL1  KLC1  KPNA2  KRT81  KRTAP4-4  KYNU  L1TD1  LACTB  LAMA4  LAMB3  LBXCOR1  LDHAL6B  LEPRE1  LEPREL2  LGI2  LHCGR  LLGL2  LMOD1  LOC347475  LOC644994  LOC648272  LOC653720  LOC728350  LRFN2  LRP10  LRP5L  LRRC3  LRRC50  LRRCC1  LRSAM1  LSM10  LY6D  LYN  MAGEB2  MAGI1_HUMAN  MAP3K10  MAP3K12  MAPK10  MARCH8  MAST2  MBD1  MBD2  MCM5  MCOLN3  MDM2  MDM4  MED12  MED19  MEGF8  MEPE  MESDC1  MFSD2  MIA3  MINA  MOBP  MORF4L1  MORN2  MOXD1  MPP6  MPZL1  MRAP  MRGPRX2  MRPS14  MRPS18B  MRPS35  MRPS5  MRPS7  MRPS9  MS4A1  MSH2  MSMB  MSN  MSRB2  MST1  MTAC2D1  MTAP  MTMR1  MTX2  MVK  MVP  MYBL2  MYL2  MYO1A  MYO1C  MYO7A  MYOD1  NAP1L2  NAPA  NBEAL2  NBPF20  NBPF3  NDE1  NDUFA1  NDUFA2  NDUFB8  NEFH  NELFB_HUMAN  NFYC  NGRN  NKX2-4  NLK  NM_018652.4  NM_207477  NOL6  NOSTRIN  NOX4  NP_001001343.1  NP_001001663.1  NP_001001665.2  NP_001007538.1  NP_001007552.1  NP_001008739.1  NP_001009993.2  NP_001011538.1  NP_001017370.1  NP_001017417.1  NP_001017421.1  NP_001017435.1  NP_001017438.1  NP_001019848.1  NP_001026789.1  NP_001030016.1  NP_001030177.1  NP_001067.2  NP_001070148.1  NP_056409.1  NP_056519.1  NP_060223.2  NP_060358.2  NP_060505.1  NP_060887.1  NP_061122.4  NP_061157.2  NP_064597.1  NP_112203.1  NP_115767.1  NP_116231.2  NP_640336.1  NP_775734.1  NP_775835.2  NP_775916.1  NP_775949.1  NP_777590.1  NP_847884.1  NP_849190.1  NP_877438.2  NP_945352.1  NP_996849.2  NP_997254.2  NP_997319.1  NR_002217.1  NR1I2  NR4A1  NRSN1  NSMCE4A  NSUN3  NSUN6  NTF3  NUDT5  NUTF2  O00431_HUMAN  OBFC2B  OCA2  OIT3  OR10H5  OR11K1P  OR2G2  OR4C3  OR4F6  OR4L1  OR52A1  OR5AR1  OR5AS1  OR5R1  OR6W1P  OR7E24  OR8U1  OVCH1  OXCT1  P2RY12  PAF1  PAK1  PARP4  PAX4  PBK  PCDHB14  PCMTD1  PCSK1  PDCD10  PDE3A  PDE6A  PDF  PDGFA  PDHB  PEBPL_HUMAN  PER1  PES1  PFDN6  PFKFB3  PGBD3  PGLYRP1  PIGC  PIGO  PIGT  PIK4CB  PKM2  PKMYT1  PLAUR  PLCL2  PLD5  PLEKHF1  PLS3  PMS2L2  PNPLA10P  POLB  POLR2G  PPIL1  PPM1L  PPP2R5E  PQLC3  PRDM2  PRKAR2A  PRKCD  PRKCDBP  PRKRA  PRMT2  PRPF18  PRPF31  PRPS1  PRR6  PRSS36  PRY_HUMAN  PSG2  PSG8  PSL2_HUMAN  PSMB5  PSMB7  PSMD13  PTPLAD1  PUM2  PWP2  PXMP4  Q2MV58-6  Q2T9F4_HUMAN  Q53R30_HUMAN  Q58FF2_HUMAN  Q59FN2_HUMAN  Q5JRB8_HUMAN  Q5T909_HUMAN  Q68DE7_HUMAN  Q68DH1_HUMAN  Q6SA06_HUMAN  Q6ZMW3_HUMAN  Q6ZN48_HUMAN  Q6ZP58_HUMAN  Q6ZRB1_HUMAN  Q6ZRZ6_HUMAN  Q6ZS71_HUMAN  Q6ZSA4_HUMAN  Q6ZSH4_HUMAN  Q6ZTB0_HUMAN  Q6ZTH0_HUMAN  Q6ZTW2_HUMAN  Q6ZTY5_HUMAN  Q6ZV23_HUMAN  Q6ZV49_HUMAN  Q8IYX2_HUMAN  Q8N2E2_HUMAN  Q8N2W8_HUMAN  Q8N4W5_HUMAN  Q8N7S6_HUMAN  Q8N8L5_HUMAN  Q8NBD0_HUMAN  Q8WYW5_HUMAN  Q96EQ7_HUMAN  Q96FU4_HUMAN  Q96M56_HUMAN  Q96MG3_HUMAN  Q96RH9_HUMAN  Q9H1T4_HUMAN  Q9H3B1_HUMAN  Q9H606_HUMAN  Q9H7S7_HUMAN  Q9H994_HUMAN  Q9P0D4_HUMAN  Q9UHU7_HUMAN  QARS  QPCT  RAB11FIP3  RAB17  RAD50  RAD51  RAD54L  RALGPS1  RALGPS2  RASAL2  RBCK1  RBM17  RBM24  RBM3  RBM5  RBP2  RBPJ  RGS13  RGS2  RHEBL1  RIMS2  RIN2  RNF215  RNUXA  RPA4  RPL12  RPL8  RPS20  RRP12  RSBN1L  RTTN  RUNDC2B  RWDD2  SAMD8  SBK1  SC5DL  SCAND2  SCG5  SCGB3A2  SCN9A  SEC24B  SEC24C  SECISBP2  SELE  SELO_HUMAN  SEMA3B  SEMA4B  SEMG1  SEP15_HUMAN  SERINC4  SERPINA4  SERPINA7  SERPINB2  SEZ6L  SF3B1  SF3B4  SFRS3  SFT2D3  SG493_HUMAN  SGCE  SH3BP2  SHB  SHPRH  SIGLECP3  SLC24A5  SLC25A11  SLC25A15  SLC25A37  SLC25A39  SLC27A3  SLC27A5  SLC28A1  SLC35A4  SLC35F5  SLC39A13  SLC4A1AP  SLC7A4  SLFNL1  SMARCA4  SMARCA5  SMPD2  SNAPC4  SNCB  SNRPD3  SNTB1  SNX27  SP140  SPAG11A  SPHK1  SPNS3  SPSB3  SPTBN1  SPTBN5  SRGN  SSRP1  SSTR4  ST6GAL1  STAC  STAMBP  STAMBPL1  STARD3NL  STMN1  SULT1E1  SUMF1  SYBU_HUMAN  SYCE2  SYCP1  TACR2  TAF11  TAF1A  TAF7L  TARS2  TBC1D7  TBK1  TBX1  TCEAL1  TCF7L2  TCHHL1  TCTA  TDRD10  TDRD7  TELO2  TFG  TFR2  THBS4  TIAM1  TICAM1  TIGD6  TLR1  TM2D3  TM4SF4  TM9SF2  TMEM118  TMEM126A  TMEM154  TMEM179  TMEM185A  TMEM41A  TMEM60  TMEM63C  TMEM85  TMEM87B  TMIGD1  TMPRSS9  TNFSF13  TP53BP2  TPD52L1  TPI1  TRAF2  TRAF3IP3  TRAV1-1  TRAV23/DV6  TRAV40  TRAV9-1  TRBV4-2  TREX1  TRGC2  TRIM3  TRIM47  TRIM73  TRIM74  TRIP11  TRIT1  TRPC3  TRPT1  TSEN54  TSKU  TTC15  TTC8  TTC9B  TTLL2  TUSC3  TXK  TYBN_HUMAN  TYR  U655_HUMAN  UBAP2L  UBE2D3  UBOX5  UFM1  UGT2B15  UHRF2  UMOD  UNC84A  UNC93A  URP2_HUMAN  USP18  USP27X  USP45  USP47  UTP14A  UTP18  VAMP8  VDAC3  VIT  VN1R1  VPS11  WBSCR22  WDR1  WDR4  WDR57  WDR6  WDR61  WDR89  WIPF1  WISP3  WRN  WSB2  WWTR1  XKRX  XR_015353.1  XR_016629.1  XR_016643.1  XR_017721.1  XR_017872.1  XRCC6  YEATS2  YLPM1  YPEL3  ZBTB26  ZBTB41  ZBTB48  ZCWPW1  ZDHHC11  ZFAT1  ZFPL1  ZFPM2  ZIM3  ZKSCAN4  ZNF136  ZNF167  ZNF181  ZNF251  ZNF253  ZNF286A  ZNF302  ZNF382  ZNF385  ZNF419  ZNF479  ZNF557  ZNF566  ZNF616  ZNF681  ZNF684  ZNF700  ZNF773  ZNF85  ZNHIT3  ZSWIM4  ABCC5  ADAM11  AIP1  AMPH  APIG1  ARNT  ASB7  ATXN1  BBX  BICD2  BMI1  BNIP3L  C10orf86  C1orf22  C6orf134  CBFB  CD4  CDK11  CDKNB1B  CNR1  COAS2  CPEB4  CPNE8  CTCF  DACH1  DBCCR1L  DBN1  DDR1  DHX15  DHX35  DKFZp667B0210  DLX1  DYRK1A  EFO1  EIF5A2  ESR1  ETS1  ETS2  FAT2  FDNC3  FLJ10159  FLJ105546  FLJ11011  FLJ13576  FLJ20366  FLJ25476  FLJ3089  FLJ31434  FLJ35954  FLJ39155  FLOT2  FOXJ3  FUSIP1  GNAI3  GNAO1  GPR51  H41  HDAC4  HIPK1  HMGCR  HOXB5  HOXC10  HRB  HRT7  HSHIN1  hsyn  HTLF  HTR7  IMP-2  INSIG1  KIAA0252  KIAA0553  KIAA0779  KIAA1036  KIAA1078  KIAA1598  KLF12  KNS2  LOC220594  LOC284058  LOC92558  MAT2A  MCSP  MEDSC1  MEIS1  MSCP  MYLIP  MYO10  NAP1L5  NBEA  NDEL1  NOTCH3  NRK  OGT  OSBPL3  OSBPL7  PAIP1  PAIP2  PBX3  PCDHA1  PCDHA10  PCDHA11  PCDHA12  PCDHA13  PCDHA2  PCDHA3  PCDHA4  PCDHA5  PCDHA6  PCDHA7  PCDHA8  PCDHA9  PELI1  PGGT1B  PHF2  PIGF  PLCG1  PLEKHC1  PLXND1  POGZ  POU3F2  PPARGC1A  PPP1R8  PPP6C  PRG3  PTEN  RAB1A  RAD9B  RALA  RAP1B  RBBP7  RBMS1  RFX3  RIMS3  RKHD3  RNF3  RP42  RSBN1  SAFB  SDC2  SEMA4G  SEMA6D  SFRP2  SHANK2  SLC1A2  SLC4A4  SMARCA1  SNAP25  SOX4  STK17B  STK4  TCF12  TEAD1  TIMP3  TMEM16A  TMEM25  TOX  TRPS1  UBE2E3  UBE2J1  UNC84B  USP21  USP32  USP6  VAPB  VGLL4  WDR40A  WHSC1  WNT1  YWHAG  ZBF278  ZF  ZFHX1B  ZNF278  Ago2  Ago4  1110002E22Rik  2510009E07Rik  2900011O08Rik  4930571K23Rik  8030462N17Rik  Aak1  Abhd3  Adipor1  Ado  Agfg1  Agps  AI314180  AI593442  Aida  Ajap1  Akap13  Akap5  Akap6  Amigo1  Ammecr1  Anapc16  Ank2  Ankib1  Ankrd52  Ap3m1  Appbp2  Arhgap42  Arhgef7  Asph  Asxl3  Atad2b  Atf7  Axin2  Azi2  Baalc  Baz2b  BC030336  Bcl11b  Bcl2l11  Bend4  Bmp2k  Bpgm  Braf  Brwd1  Brwd3  C8a  Cacnb4  Camk1d  Camkk1  Camta1  Cand1  Ccdc171  Ccdc88a  Cd164  Cd2ap  Cd47  Cdc5l  Cdh2  Cdk19  Cdk8  Cdkn1b  Cdon  Cdv3  Cdyl2  Cenpo  Chd7  Chfr  Chsy1  Cldn25  Clgn  Clint1  Clvs2  Cmip  Cmtm4  Cnot2  Cnot6  Cntln  Col4a4  Commd1  Cpeb3  Creb1  Crebzf  Crkl  Csk  Csnk2a1  Ctif  Cttn  Cux2  Cxadr  Cxcl11  Cyp7a1  Cyp7b1  Dbt  Dcaf12  Dcaf7  Dcun1d1  Dcun1d4  Dennd1b  Dgke  Dicer1  Dkk2  Dlg2  Dnajb14  Dnal1  Dpp8  Dusp3  Dync1li2  E130309F12Rik  E2f2  Efnb2  Egfr  Ehf  Eif3j1  Eif4e3  Elavl2  Elp4  Eml6  Emx2  Epb4.1l1  Erbb3  Erbb4  Ercc4  Esyt1  Etv3  Fam120a  Fam167a  Fam196b  Fam199x  Fam208a  Fam214a  Fam222b  Fbn2  Fbxo28  Fem1b  Fem1c  Fermt2  Fgf14  Fgf4  Fignl2  Fkbp9  Fmr1  Fndc3a  Fndc3b  Fnip2  Foxa1  Foxn2  Foxp1  Foxp2  Fpgt  Frat2  Frk  Frrs1l  Frs2  Fry  Fubp1  Gab1  Gab2  Galnt18  Galnt3  Gbx2  Gm10295  Gm4861  Gm7008  Gm996  Gpalpp1  Gpbp1  Gpm6a  Gpr12  Gpr165  Gpr22  Gpr68  Grb10  Gtf2a1  Gtf2b  Gtf2e1  Gucy1a2  H3f3a  Hcn4  Heg1  Higd1c  Hipk2  Hipk3  Hmbox1  Hnrnpa3  Hnrnpd  Hnrnph3  Htt  Ick  Igf1  Igsf3  Ikzf4  Il1rapl1  Il21  Il34  Ipo7  Iqsec1  Itga2  Itga3  Itgb3  Itgbl1  Kansl1  Kbtbd8  Kcnh7  Kcnq3  Kdr  Kdsr  Kif16b  Kif5c  Kit  Klf7  Klhl18  Klhl24  Kmt2a  Kmt2c  Kpna1  L3mbtl1  Lhfpl2  Lhx8  Lifr  Lmbrd2  Loxl3  Lrrc4c  Lrrtm2  Luc7l3  Lypla1  Lysmd1  Map3k2  Mapre1  Marcks  Mark1  Med1  Megf9  Mettl7a2Higd1c  Mex3a  Mfn1  Mfsd5  Midn  Mier3  Mios  Mitf  Mllt6  Mon2  Mprip  Mrpl39  Msantd2  Msl2  Mterfd3  Mtmr2  Mxd1  Mybl1  Myef2  Myt1l  Naa25  Nanos1  Nap1l1  Nbeal1  Ncam1  Ndfip1  Ndst3  Ndufab1  Nfatc3  Nfyb  Nipa1  Nipal4  Nkiras1  Nop9  Nova1  Nrg1  Nsun4  Nsun7  Nufip2  Nxph1  Nyap2  Onecut1  Ostm1  Oxa1l  P2ry4  Padi1  Pank3  Paqr9  Pcdh9  Pcgf3  Pde1c  Pdik1l  Pdzrn4  Peg3  Phc2  Phf12  Phip  Piezo2  Pik3r1  Pitpnm2  Pkdcc  Pkia  Plcxd3  Plekha2  Plekha6  Plxnc1  Pno1  Polr3e  Ppm1h  Ppp1r15b  Ppp2r2a  Ppp3r1  Prdm11  Prom2  Prr14l  Prrg3  Prss53  Prune  Psma2  Ptbp2  Ptbp3  Ptchd1  Ptchd4  Ptk2  Ptpn3  Ptprk  Pura  Pvrl1  Qk  Rab1  Rab18  Rab3gap2  Ralgapa1  Ranbp10  Ranbp2  Rap2b  Rapgef2  Rc3h2  Reck  Rfx7  Rfx8  Rgs17  Rilpl1  Rims4  Rnf165  Rnf4  Rnf44  Rnps1  Rph3a  Rreb1  Rsf1  Runx2  S100pbp  Scarf2  Scml4  Sdhaf2  Sec23ip  Sec62  Sesn2  Sesn3  Sfpq  Sh3bgrl  Sh3bp4  Sh3pxd2b  Shoc2  Skor1  Skp1a  Slc16a6  Slc2a13  Slc2a2  Slc30a1  Slc40a1  Slitrk5  Smarca2  Snx29  Snx4  Socs3  Socs7  Sox1  Sox10  Sox11  Spata2  Spats2l  Spon1  Spred2  Sptssa  Srpk2  Srsf2  St8sia1  Stk24  Stox2  Stx1b  Styx  Sugt1  Sun2  Swt1  Syn3  Syncrip  Syt10  Taf1  Tardbp  Tbc1d19  Tbc1d22b  Tcf4  Tet1  Tfap2a  Thbs1  Thrb  Timp2  Tiparp  Tle3  Tmcc1  Tmem106a  Tmem132b  Tmem2  Tnfrsf11b  Tnrc6b  Tnrc6c  Tomm40l  Trabd2b  Tram1  Tram2  Trp53inp1  Trp53inp2  Tspan13  Ttbk2  Tub  Tuba1a  Ubn2  Uri1  Usp49  Usp6nl  Vash1  Vezf1  Vkorc1l1  Vti1a  Wdr20  Wdr35  Wdr37  Wdr47  Wee1  Wls  Wnk3  Xirp2  Ythdc1  Zadh2  Zdhhc17  Zeb2  Zfand5  Zfhx3  Zfp113  Zfp142  Zfp326  Zfp36l2  Zfp385a  Zfp518b  Zfp618  Zfp652  Zfp9  Zfyve16  Zmym2  Zxdb  Zyx | A_23_P112957  A_23_P158868  A_23_P159163  A_23_P28743  A_23_P435390  A_23_P44053  A_23_P51966  A_23_P72252  A_23_P84791  A_24_P118422  A_24_P147849  A_24_P152315  A_24_P161853  A_24_P186354  A_24_P194954  A_24_P203886  A_24_P204144  A_24_P204574  A_24_P204604  A_24_P234871  A_24_P24806  A_24_P25020  A_24_P281504  A_24_P298179  A_24_P325533  A_24_P341126  A_24_P341376  A_24_P341408  A_24_P349869  A_24_P384119  A_24_P384604  A_24_P401150  A_24_P490109  A_24_P508946  A_24_P552987  A_24_P698376  A_24_P7330  A_24_P755069  A_24_P799580  A_24_P814246  A_24_P84711  A_32_P108592  A_32_P169353  A_32_P19460  A_32_P208713  A_32_P214565  A_32_P53670  A_32_P78285  A_32_P93894  A_32_P99804  A2M  AA420998  AA554330  AAAS  AADACL1  ABCB10  ABCB6  ABCC13  ABCF1  ABCF2  ABCG2  ABHD14A  ACAA2  ACAD9  ACADS  ACHE  ACO2  ACOT1  ACOT2  ACOT7  ACSBG1  ACSL6  ACY1  ADAT1  ADD1  ADD2  ADIPOR1  AF038185  AF063695  AF076205  AF086139  AF086448  AF267875  AF289562  AF332145  AF343666  AF471454  AFARP1  AFF1  AFG3L2  AGPAT3  AGPAT4  AIFM2  AJ009817  AJ319669  AJ399872  AJ519285  AK022030  AK023159  AK023559  AK024898  AK026372  AK026826  AK055981  AK057652  AK074614  AK090416  AK093617  AK095108  AK095167  AK095583  AK095707  AK098081  AK098422  AK1  AK123096  AK124299  AK125299  AK125361  AK127768  AK130930  AKAP7  AKR1C3  AKR7A3  AKT1S1  AL117621  AL522622  ALAD  ALAS2  ALDH16A1  ALDH18A1  ALDH4A1  ALDH5A1  ALG1  ALG2  ALG3  ALS2  ALS2CR2  AMMECR1  ANK1  ANKH  ANKRD25  ANKRD33  ANKRD41  ANKRD9  AP2A1  AP2A2  AP2B1  AP2M1  AP2S1  AP3D1  APEH  APEX2  APLN  APOBEC3C  APOBEC3F  APOL3  APOM  APRIN  AQP1  AQP3  ARHGAP19  ARHGAP23  ARHGEF12  ARL1  ARL2  ARL2BP  ARL4A  ARMC1  ARPC1A  ART4  ARVCF  ASCC2  ASF1A  ASNA1  ASXL2  ATG4A  ATP1A1  ATP1B1  ATP1B2  ATP5A1  ATP7B  ATRN  AW302758  AW804491  AY003763  AY062331  AY172962  AY358510  AY998685  B4GALT7  BACE2  BAD  BAG1  BAG5  BC000986  BC007606  BC012876  BC013077  BC014395  BC018095  BC022362  BC030813  BC031344  BC032451  BC035146  BC038512  BC039021  BC039479  BC040991  BC062753  BC063426  BC087732  BCL11B  BCL2  BCL2L1  BCL2L11  BE893137  BF965065  BG259864  BG547557  BIRC5  BLVRB  BM455859  BM479752  BMP2K  BNIP3L  BOLA3  BPGM  BQ017638  BRD3  BSCL2  BSG  BTBD7  BTG2  BTRC  BU732811  BU940040  BX105952  BX537432  C10orf12  C10orf61  C11orf77  C13orf8  C14orf1  C14orf130  C14orf139  C14orf169  C14orf32  C14orf45  C16orf35  C16orf58  C16orf68  C17orf71  C17orf76  C18orf10  C18orf24  C18orf56  C19orf43  C19orf48  C19orf52  C19orf57  C1orf109  C1orf128  C1orf142  C1orf163  C1orf164  C1orf166  C1orf198  C1orf26  C1orf77  C1orf93  C20orf108  C20orf11  C20orf121  C20orf141  C20orf175  C20orf29  C20orf4  C20orf55  C21orf45  C22orf13  C22orf25  C22orf28  C22orf9  C2orf24  C3orf39  C4B  C5orf30  C5orf4  C6orf129  C6orf25  C6orf59  C6orf85  C6orf89  C7orf41  C7orf49  C8orf55  C9orf114  C9orf125  C9orf40  C9orf5  C9orf58  C9orf78  CA1  CA2  CA3  CA314936  CALM3  CAPN1  CAT  CBX7  CCDC117  CCDC124  CCDC47  CCDC86  CCDC92  CCDC94  CCNA2  CCNB1  CCNB2  CCNDBP1  CCNE1  CCR2  CCR5  CCRL2  CCS  CCT2  CD242823  CD248  CD2AP  CD36  CD40  CD59  CD612636  CD709370  CD8A  CD8B  CD99  CD99L2  CDC20  CDC25A  CDC27  CDC34  CDC42BPA  CDC42BPB  CDCA4  CDCA8  CDH1  CDKN2C  CDYL  CENPF  CENPO  CENPP  CGI-115  CHAC2  CHCHD3  CHID1  CHST2  CIRH1A  CIT  CIZ1  CKAP2  CKAP2L  CKAP5  CLCN3  CLDN5  CLEC1B  CLN6  CLPB  CLTC  CMAS  CMTM5  COASY  COG8  COL4A2  COL6A1  COMT  COPE  COQ7  CPOX  CPVL  CR596214  CR601260  CR603982  CR605719  CR606637  CR611332  CR617018  CR617560  CRAT  CRSP8  CRY1  CRYL1  CSDA  CSTF2T  CTA-246H3.1  CTB-1048E9.5  CTCF  CTDSPL  CTNNA1  CTNNAL1  CTSB  CTSE  CTSL  CUL4A  CXCL12  CXCR3  CXCR6  CXorf6  CYB5A  CYB5R3  CYBASC3  CYBRD1  CYC1  D83692  DAAM1  DAB2  DAG1  DCK  DCLRE1A  DCN  DCUN1D1  DCXR  DDB1  DDHD2  DDOST  DDX28  DEAF1  DECR2  DENND4A  DEPDC1B  DERL3  DEXI  DGCR6  DGCR6L  DGCR8  DHFR  DHRS13  DIAPH3  DKFZP686E2158  DKFZP761M1511  DKFZp779O175  DLC1  DLEU2  DLG7  DNAJA4  DNAJB2  DNAJC11  DNAJC7  DNAJC9  DNASE2  DNM3  DOHH  DOLPP1  DPF3  DPM2  DPP3  DQ100840  DQ680071  DTL  DTYMK  DUSP14  DYRK3  E2F2  E2F4  E2F7  E2F8  EB386378  ECH1  EDG1  EEF2  EEF2K  EFNB3  EGFL8  EHD3  EI24  EIF2AK1  EIF3S1  EIF3S9  EIF4EBP2  EIF4G1  EIF5A  EIF5A2  ELAC2  ELOF1  ELOVL1  EMID1  ENDOD1  ENDOG  ENG  ENO2  ENST00000252134  ENST00000259219  ENST00000283657  ENST00000295339  ENST00000295410  ENST00000296873  ENST00000307840  ENST00000311061  ENST00000312946  ENST00000322032  ENST00000326261  ENST00000327926  ENST00000331195  ENST00000331696  ENST00000339367  ENST00000354689  ENST00000355691  ENST00000358618  ENST00000359488  ENST00000360102  ENST00000370857  ENST00000371189  ENST00000374390  ENST00000377221  ENST00000377226  ENST00000377233  ENST00000379877  ENST00000379879  ENST00000379895  ENST00000379913  ENST00000380344  ENST00000383048  EPB41  EPB42  EPB49  EPC2  EPN2  EPOR  EPPB9  EPRS  ERAF  ERGIC3  ERMAP  EXOD1  FAH  FAHD1  FAHD2A  FAM100A  FAM104A  FAM109B  FAM117A  FAM122A  FAM127B  FAM129B  FAM13A1  FAM20B  FAM33A  FAM46C  FAM82C  FAM83D  FANCC  FARSLA  FBL  FBXO18  FBXO21  FBXO30  FBXO31  FBXO34  FBXO7  FBXW11  FCER1A  FECH  FEM1A  FEN1  FGFR3  FGFRL1  FH  FHL2  FIS1  FKBP1B  FKBP8  FLJ12331  FLJ14981  FLJ20105  FLJ20489  FLJ22222  FLJ30092  FLJ36208  FLJ41603  FN3KRP  FNBP1L  FOXM1  FOXO3A  FOXRED2  FREQ  FRMD4A  FRZB  FSTL1  FTSJ2  FUNDC2  FUT1  FXN  FZD1  FZD5  FZR1  GABRP  GAD1  GALNT6  GAS2L1  GATA1  GBGT1  GCDH  GCLC  GCLM  GDPD5  GEMIN4  GFI1B  GINS3  GLG1  GLRX5  GMEB1  GMPPB  GMPR  GNA12  GNAZ  GNPDA1  GOLGA4  GOSR2  GOT1  GP1BA  GPAA1  GPAM  GPR132  GPR137B  GPR146  GPR175  GPX1  GRHPR  GRPEL2  GRRP1  GRWD1  GSG2  GSPT1  GSTT1  GTF3C4  GTPBP4  GTSE1  GUK1  GYPA  GYPB  GYPC  GYPE  GZMK  HAGH  HBA1  HBA2  HBD  HBLD2  HBM  HBQ1  HBZ  HCCA2  HCCS  HDAC4  HDGF  HDLBP  HEBP1  HEMGN  HES5  HEXA  HINT2  HIP2  HK1  HLA-DQA2  HLTF  HMBS  HMG2L1  HMGA1  HN1L  HNRPAB  HNRPUL1  HNRPUL2  HPS6  HSMPP8  HSPBP1  HSPC142  HTRA1  HTRA2  HYLS1  ICAM4  ICMT  IDH2  IFI6  IFRD2  IFT122  IGH@  IGHA1  IGHG1  IGJ  IGKC  IGKV1-5  IGKV2-24  IGLL1  IGLV6-57  IL15RA  IL18BP  IL2RB  IL32  IL8  ILVBL  IMP3  IMPDH2  INTS5  IQSEC2  IQWD1  ITGA2B  ITGB1  ITGB3  ITGB5  ITLN1  ITSN1  JAKMIP1  JAZF1  K-ALPHA-1  KATNAL1  KBTBD4  KCNH2  KCNJ12  KCNN4  KCTD5  KEAP1  KEL  KHSRP  KIAA0133  KIAA0323  KIAA0355  KIAA0406  KIAA0485  KIAA0652  KIAA1147  KIAA1155  KIAA1191  KIAA1303  KIAA1344  KIAA1450  KIAA1542  KIAA1727  KIAA1815  KIF11  KIF21A  KIF22  KIF26A  KIF4A  KIFC1  KLF1  KLF13  KLF3  KLHDC3  KLHDC8A  KLHDC8B  KREMEN1  KRT1  L38427  L3MBTL2  LAGE3  LANCL2  LBH  LCMT2  LDOC1L  LEPREL1  LEPROTL1  LGR4  LGR6  LHFPL2  LIG1  LIN9  LIPA  LMAN2  LMNA  LNX2  LOC134357  LOC146346  LOC152663  LOC158345  LOC200810  LOC253012  LOC283177  LOC283666  LOC340508  LOC343508  LOC388524  LOC388588  LOC391559  LOC442239  LOC442308  LOC494150  LOC642413  LOC643013  LOC643960  LOC643992  LOC644462  LOC645000  LOC652012  LOC652254  LOC731076  LOC91461  LPIN2  LRBA  LRRC20  LRRC8A  LRRN5  LTBP1  LY9  LYCAT  M87790  MAF  MAF1  MAL  MAN2A1  MAP2K3  MAP3K7IP3  MARCH2  MARCH3  MARCH8  MARVELD2  MAZ  MBNL2  MBOAT5  MBP  MCAT  MCM10  MCM2  MCM4  MCM5  MCM6  MCM7  MED9  METAP2  MFAP1  MFSD5  MGC11102  MGC11257  MGC12760  MGC13057  MGC14327  MGC15523  MGC17403  MGC27348  MGC29891  MGC4677  MGC5139  MGST3  MICAL2  MICALCL  MINPP1  MIPEP  MIS12  MKI67  MKRN1  MLLT7  MOBKL1A  MOBKL2C  MOSPD1  MPDU1  MPP1  MRFAP1  MRFAP1L1  MRPL37  MRPL46  MRPL49  MRPS2  MST1  MSTP9  MTCH2  MTHFD1  MTMR12  MTMR2  MXD4  MXI1  MXRA8  MYBL2  MYH10  MYL4  NAG  NAGPA  NAP1L4  NAP1L5  NAPA  NARF  NAT12  NCAPH  NCOA4  NCR3  NDUFC2  NDUFS2  NDUFV3  NEDD4L  NEK2  NELL2  NF2  NFIA  NFIX  NGRN  NIP7  NME4  NMNAT3  NOL9  NOLA1  NOLA2  NP  NP109842  NPAL3  NT5M  NUDT21  NUDT4  NUP133  NUP188  NUP93  NUS1  NUTF2  NYX  OAT  ODC1  OGFOD2  OLFML2B  OPA1  OPTN  OR2W3  ORC1L  OSBP2  OXNAD1  P4HB  PACSIN1  PAFAH1B1  PAGE2  PAGE5  PAICS  PAIP1  PAQR4  PAQR9  PAXIP1  PBX1  PC  PCAF  PCK2  PDCD1  PDCL  PDK2  PDXP  PDZK1IP1  PEPD  PES1  PET112L  PEX10  PFDN6  PFKM  PGM2L1  PGRMC2  PHOSPHO1  PHOSPHO2  PI4KII  PIGC  PIGQ  PIGW  PIM1  PIP5K1B  PIP5K2A  PIR  PITPNA  PKLR  PLEK2  PLEKHF1  PLOD3  PLTP  POLDIP2  POLE3  POLL  POLR1C  POLR3C  POP7  PPAP2B  PPBP  PPIA  PPME1  PPOX  PPP1R8  PPP2R1B  PPP2R4  PPT2  PQLC1  PRDX2  PRDX6  PRKAR2B  PRMT6  PROP1  PRPF19  PRPF8  PRPS1  PRR15  PRR5  PRRT3  PSKH1  PSMB5  PSMB6  PSMC3  PSMC4  PSMC5  PSMD1  PSMD12  PSMF1  PUS1  PVRL1  PYCR2  PYCRL  QARS  QDPR  QSCN6L1  QTRT1  RAB2  RAB3IL1  RAB6B  RAD23A  RAD51C  RAN  RANBP10  RANGAP1  RAP2A  RAP2B  RAPGEF2  RASGRP3  RASIP1  RBM13  RBM15B  RBM38  RBX1  RCCD1  RCL1  RFESD  RG9MTD1  RGC32  RGS10  RGS16  RHAG  RHBDD1  RHCE  RHD  RIC8A  RILP  RKHD1  RMND5A  RMND5B  RNASE1  RNF121  RNF123  RNF14  RNF182  RNF187  RNF26  RNF5  RNF6  RNH1  RNMTL1  RNPS1  RP11-11C5.2  RP11-529I10.4  RPA1  RPIA  RPL4  RPL7A  RPN2  RPP38  RPS3  RPUSD2  RRM1  RRM2  RSC1A1  RUVBL2  S76132  SAMD1  SAMM50  SAPS2  SAV1  SDC1  SDF2L1  SDF4  SDHC  SDSL  SEC14L4  SEH1L  SELENBP1  SELM  SELS  SEPHS1  SEPP1  SESN3  SF3B3  SFRS2B  SFT2D3  SH3BGRL2  SH3GLB2  SHARPIN  SHCBP1  SHMT1  SIAHBP1  SIGLECP3  SIPA1L1  SIVA1  SLAMF7  SLBP  SLC11A2  SLC12A7  SLC14A1  SLC1A5  SLC22A4  SLC25A15  SLC25A21  SLC25A37  SLC25A38  SLC25A39  SLC25A42  SLC29A1  SLC2A1  SLC2A4RG  SLC39A3  SLC39A8  SLC43A1  SLC43A3  SLC4A1  SLC6A10P  SLC6A8  SLC7A1  SLC7A5  SMC1A  SMC3  SMOX  SNCA  SND1  SNX22  SNX9  SOD1  SPBC24  SPECC1  SPN  SPRYD4  SPTB  SRD5A2L  SRM  SSBP3  ST7  STAU1  STCH  STEAP3  STK25  STOML2  SUPT16H  SUV39H1  TACC3  TAF4  TAF5L  TAL1  TBC1D14  TBCEL  TCEA1  TCEB3  TCF19  TCF20  TERF2IP  TFDP1  TFRC  TGM2  TGS1  THAP11  THC2478531  THC2487640  THC2497143  THC2500271  THC2506002  THC2516487  THC2522223  THC2527647  THC2527772  THC2530075  THC2532155  THC2541642  THC2551769  THC2588392  THC2609092  THC2613527  THC2618446  THC2633438  THC2636523  THC2638232  THC2649313  THC2654231  THC2656519  THC2660636  THC2663668  THC2664480  THC2669092  THC2671299  THC2672257  THC2685096  THC2685373  THC2688497  THC2689491  THC2707284  THC2730631  THOC6  TIGD5  TIGD6  TIMD4  TIMM22  TIMM23  TIMM44  TIMP3  TK1  TLN2  TLOC1  TMCC2  TMEM111  TMEM138  TMEM15  TMEM23  TMEM48  TMEM56  TMEM57  TMEM58  TMEM64  TMEM85  TMEM86B  TMEM9B  TMEPAI  TMOD1  TMPRSS5  TMPRSS9  TNS1  TNXB  TOE1  TOP1P2  TRAF3IP1  TRAK2  TRAM2  TRAP1  TREML1  TRIM10  TRIM58  TRIM59  TSFM  TSPAN17  TSPAN4  TSPAN5  TSPYL1  TTC25  TTC4  TUBA3  TUBA6  TUBA8  TUBB  TUBB1  TUBB2A  TUBB3  TUBB4  TUBB6  TUBG1  TUBG2  TUSC1  TXN2  TXNDC5  TXNRD2  UBADC1  UBB  UBE2H  UBE2O  UBL4A  UBXD1  UBXD3  UCP2  UHMK1  UMPS  UNQ1887  UROS  USP12  USP14  USP31  USP5  VANGL1  VCAM1  VCP  VKORC1  VPS37C  VPS41  VTI1B  VWCE  VWF  WASF2  WBSCR16  WBSCR22  WDR13  WDR23  WDR32  WDR34  WDR40A  WDR76  WDR81  WDR89  WHSC1  WIPI2  WNK1  WRN  WSB2  X01147  X57802  XK  XPO4  XPO7  YBX1  YIF1A  YIPF6  YOD1  YWHAG  Z18824  ZAK  ZBTB32  ZBTB38  ZCD2  ZDHHC14  ZDHHC5  ZFYVE21  ZHX1  ZNF134  ZNF16  ZNF175  ZNF23  ZNF264  ZNF268  ZNF289  ZNF364  ZNF526  ZNF543  ZNF551  ZNF557  ZNF584  ZNF652  ZNF672  ZNF689  ZNF696  ZSCAN21  ZSCAN5 |  |
|  |  |  |
